# Barriers and Facilitators to Accessing Addiction Treatment Services for Sexual Minority (LGBQ+) People: A Systematic Review

**DOI:** 10.1192/bjp.2026.10589

**Published:** 2026-03-30

**Authors:** Miriam Hillyard, Beatrice Cockbain, Colin Drummond, Katharine Rimes, Emmert Roberts

**Affiliations:** 1 National Addiction Centre, Institute for Psychiatry, Psychology and Neuroscience, 4 Windsor Walk, London, SE5 8BB, UK; 2 Chelsea and Westminster NHS Foundation Trust, UK; 3 Imperial College London, Exhibition Rd, South Kensington, London, SW7 2AZ, UK; 4 School of Psychology, Portland Square, University of Plymouth, Devon, PL4 8AA; 5 South London and Maudsley NHS Foundation Trust, UK

## Abstract

**Background:**

Sexual minority (lesbian, gay, bisexual, queer, and other non-heterosexual) people experience significant disparities in addiction problems compared with heterosexual people.

**Aims:**

We aimed to answer: *what are the barriers and facilitators to accessing drug/alcohol addiction treatment services for sexual minority adults?*

**Methods:**

A systematic review (PROSPERO: CRD42021244006) was conducted by searching Medline, PsychINFO, CINAHL, Web of Science, and Sociological Abstracts for any English-language primary research article (qualitative, quantitative, or mixed-methods) relevant to the study aims, from inception up to 18/04/2025. Quality of included studies was assessed using the Mixed-Methods Appraisal Tool (MMAT-2018). Barriers and facilitators were categorised into ‘service-related’ and ‘patient-related’ dimensions of accessibility and synthesised narratively.

**Results:**

3282 abstracts were screened, with full-text review of 238 articles. 62 studies met the inclusion criteria. Common service-related barriers included explicit harassment, discrimination, violence, or abuse towards sexual minority people in services, and lack of expertise or ‘culturally competent’ provision for their specific addiction problems. Facilitators included affirming, non-judgemental staff attitudes and sexual minority-specific treatment or outreach services. Patient-related barriers included ambivalence around drug/alcohol use and a fear of stigma (e.g. around sexualised drug use). Facilitators included signposting to services via community networks or peer advocates, and allowing patients to set their own treatment goals.

**Conclusion:**

Although barriers and facilitators vary across global contexts and time periods, both qualitative and quantitative research highlighted similar key issues. Implementing practical changes to address these may improve sexual minority people’s access to addiction services, reducing the burden of addiction-related health inequity for this community.

## 1 Introduction

**Terminology Note:** Studies in this field use a variety of terms to refer to sexual and gender minoritised people, who are often described under the umbrella term ‘LGBTQ+’ (lesbian, gay, bisexual, trans, queer/questioning, and others). Here, where studies are cited, the terminology from the original paper has been preserved (e.g. LGB, LGBT, or LGBTQ+ people). For clarity we have opted to use the term ‘patient(s)’ throughout this paper, although acknowledge other terms (e.g. ‘service user’, ‘client’) may be preferred depending on context.

### 1.1 Rationale

Sexual minority (SM) individuals include lesbian, gay, bisexual, queer, and other people whose sexual attractions, identities, and behaviours are non-heterosexual. Among the range of health inequities faced by this group^1–3^, there are significant disparities in addiction problems. SM people have a higher prevalence of drug and alcohol use, and drug and alcohol use disorders, compared with heterosexual people^4–10^. They are more likely to have their addiction problems untreated^11^, or if they do enter treatment services, they often enter with more severe substance use disorders than their heterosexual counterparts^12^. Within-group inequities also exist: bisexual people tend to have more severe addiction problems than gay and lesbian people^12,13^, and may also struggle more with accessing drug treatment services^14^. Minority stress theory is the leading explanatory mechanism for these disparities^15–18^, and experiences of discrimination potentiate drug and alcohol use, and use disorders^15,19,20^.

Addiction treatment services can significantly impact drug- and alcohol-related morbidity and mortality. However, engaging and retaining SM people in treatment may be challenging. In general, SM people frequently experience negative healthcare encounters^21–24^, and may fear homophobic/biphobic discrimination or abuse within services^22,23^. They tend to receive poorer-quality care than heterosexual people^25–28^, which may result from heterosexist bias among healthcare staff^29^. Concerns about services lacking SM-specific knowledge and ‘cultural competence’ may also be barriers to access^30–32^.

For SM people, therefore, their increased health needs are often not matched by their rates of health consultation. There is significant unmet need, or a ‘treatment gap’, as a result of barriers to healthcare access^33,34^. In the addiction context, SM-targeted or specialised treatment services are one response to this, and may have better outcomes than non-targeted services^35,36^. There is a body of both qualitative and quantitative literature addressing addiction treatment access by SM people, and to our knowledge, this is the first review to examine it systematically.

### 1.2 Objectives

We aimed to answer the question ‘*What are the barriers and facilitators to accessing drug and alcohol addiction treatment services for sexual minority people?’*. We expected that SM people would experience unique barriers and facilitators to accessing addiction treatment based on their SM status (e.g. homophobia from treatment providers). There may also be general barriers and facilitators, (e.g. around health insurance status), which may be more likely to affect SM people than heterosexual people due to socio-economic disparities driven by their minority status.

## 2 Methods

This review was prospectively registered in PROSPERO (CRD42021244006), and reported according to the Preferred Reporting Items for Systematic Reviews and Meta-Analyses (PRISMA) statement^37^.

### 2.1 Eligibility Criteria

The target population was adults who:

- Identify as gay, lesbian, bisexual, queer, or other SM
- Have accessed addiction treatment services, have tried to access addiction treatment services, want to access addiction treatment services, or would benefit from accessing addiction treatment services. Services could include statutory health services, private services, and third-sector providers which provide specialist, structured interventions for drug and/or alcohol addiction.

Studies were included if they were published in English, reported on adults (>80% of participants in study ≥18 years), reported on the population defined above (or included findings from health professionals or service providers, which were relevant to the study aims), and reported on barriers and/or facilitators to access for addiction treatment services. We included qualitative, quantitative, and mixed-methods primary research studies. We excluded unpublished studies, conference abstracts, editorials, reviews, case reports, comment pieces, news articles, and books. A list of the inclusion and exclusion criteria is provided in Supplementary Table 1.

#### Information sources

A systematic search was performed using the MEDLINE, PsychINFO (via Ovid), CINAHL (via EBSCOHost), Web of Science, and Sociological Abstracts (via ProQuest) databases (searched since inception). Reference lists of included studies were also searched to identify any additional relevant studies that were not captured by the search strategy.

### 2.2 Search strategy

The search strategy (A+B+C+D) combined MeSH terms/subject headings and keywords relating to the following constructs:

A: sexual minorities

B: addiction

C: treatment service

D: barriers and facilitators

The full syntax is given in Supplementary Table 2. The initial search and data extraction was performed on 25/04/2021, and searches were re-run on 20/10/2024 prior to the final analysis to identify and extract data from any additional studies published since the original search. A final search was run on 18/04/2025 prior to submission for publication.

### 2.3 Selection process

EPPI Reviewer Web software was used for initial screening, which allowed automated de-duplication. Initial screening of titles and abstracts was performed by the first author^38^, with 10% independently checked by the second author to ensure reliability. Screening decisions were recorded in EPPI Reviewer Web. All articles that appeared to meet the inclusion criteria were included for full-text screening. The first and second authors then independently screened full-text articles against the inclusion criteria and Mixed Methods Appraisal Tool 2018 version (MMAT-2018) screening questions^39^. Decisions were recorded in a Microsoft Excel file. Any decisions where agreement could not be reached were discussed with the other authors for arbitration.

### 2.4 Data collection process

For included studies, the first and second authors independently extracted key variables and rated the quality of the included studies using the MMAT-2018. The data items extracted are presented in Supplementary Table 3.

### 2.5 Study risk of bias assessment

This systematic review used the MMAT-2018 which allows a unified scoring of qualitative, quantitative, and mixed-methods studies, resulting in a six-point rating of each study’s quality (zero ‘yes’ answers = poor quality, to five ‘yes’ answers = very good quality). The first and second authors independently rated studies and recorded decisions in a Microsoft Excel file. In the case of any rating discrepancies that could not be resolved by discussion, arbitration was sought from the other authors.

### 2.6 Effect measures

Barriers and facilitators to accessing addiction treatment services were classified by the first and second authors into dimensions of accessibility according to the model from Levesque *et al*.^40^. This model defines access as “the opportunity to reach and obtain appropriate health care services in situations of perceived need for care”. It includes five ‘service side’ factors (approachability, acceptability, availability and accommodation, affordability, and appropriateness) and five corresponding ‘patient side’ factors (ability to perceive, ability to seek, ability to reach, ability to pay, and ability to engage).

### 2.7 Synthesis methods

Individual barriers and facilitators were summarised by the first and second authors, in discussion with the other authors, into overarching themes for presentation. For example, the specific barriers ‘could not afford to pay the medical bill’ and ‘having no money to access detox services’ were summarised into the theme of ‘unable to afford medical bills/pay for addiction services’. A narrative synthesis was conducted in line with the guidance found in ‘*Guidance on the Conduct of Narrative Synthesis in Systematic Reviews’*^41^.

## 3 Results

**Figure 1:**
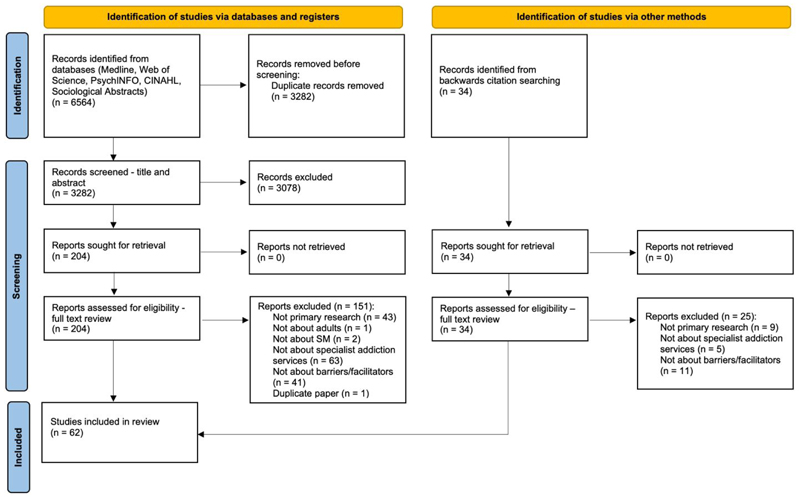
PRISMA flow diagram.

The PRISMA flow diagram (Figure 1) summarises the results of the search and screening process.

### 3.1 Study Characteristics

Of the 62 included studies, the majority (n=38) were from the USA^29,42–78^, with 8 from Canada^79–86^, 5 from the UK^87–91^, 3 from Australia^92–94^, 2 from Singapore^95,96^ and one each from Peru^97^, South Africa^98^, France^99^, Germany^100^, and Ireland^101^. There was also one global survey^102^. Supplementary Table 4 summarises the characteristics of the included studies, and Supplementary Table 5 summarises the detailed results of the MMAT-2018 quality assessment. Quality of the included papers (32 qualitative, 26 quantitative, and 4 mixed-methods) was variable. Many of the quantitative studies relied on convenience sampling with a high risk of non-response bias, and failed to account for potential confounders of the findings. Qualitative studies were typically rated as higher-quality, reflecting the utility of qualitative approaches for answering the research question, although some did not justify findings with reference to the collected data. All of the mixed-methods studies had some quality issues. Excluded studies are summarised in Supplementary Table 6.

### 3.2 Results: Service-Related Barriers to Access

A summary table of results can be seen in Supplementary Table 7.

#### 3.2.1 Approachability

Approachability in Levesque *et al*.’s model is the idea that a service “exists, can be reached, and [can] have an impact on the health of the individual”. Several studies identified services lacking specific expertise and provision around LGBTQ+ addiction issues (for example, not providing for SM patients’ specific sexual/mental health needs, or having no available services which could address sexualised drug use or methamphetamine use)^67,68,74,81,86–88,91,93,94,100^. Services failing to provide information to patients about how drug support services work and how they can help was reported by two studies^89,95^. In some health systems, fragmentation of services means patients end up ‘knocking on several doors’, ‘falling between the gaps’, or being inefficiently ‘cycled’ between organisations or service pathways before they are able to access the specific treatment they need^81,90,100^. One study reported that due to no available inpatient facilities for methamphetamine detoxification, methamphetamine-using men who have sex with men (MSM) were deliberately consuming excessive alcohol to render them eligible for a detox admission^68^. Several studies in this review focused on chemsex, which is the use of specific drugs (‘chems’, usually crystal methamphetamine, gamma-hydroxybutyrate/gamma-butyrolactone, and synthetic cathinones such as mephedrone) by MSM or LGBTQ+ people to facilitate or enhance sexual activity^103^. Chemsex participants felt that addiction services were set up for alcohol or non-chemsex drug users rather than for chemsex^88,94^, a finding echoed in a study about drug service staff experiences^91^.

#### 3.2.2 Acceptability

Acceptability relates to “the cultural and social factors determining the possibility for people to accept aspects of the service”. We have chosen to classify studies that reported on explicit harm to SM patients here, acknowledging that such harm is unacceptable. Multiple studies reported explicit harassment, discrimination, bullying, threats, violence, or abuse towards SM people within services^43,48,54,57,67,69,73,75,80,104^. Examples of this range from a lesbian patient being raped by a male staff member, claiming he would make her heterosexual^80^, to patients being denied treatment because of their sexual identity^54,67,69^. In addition, several studies reported staff failing to protect SM patients from hostility, overt homophobia, attacks, sexual violence, or abuse from non-SM patients in treatment settings^43,48,69,75,80,81^. Explicit negative or stigmatising attitudes of service staff towards SM patients were reported by twelve studies^46,48,57,58,69,75,80,85,86,91,94,105^, for example perceiving drug-using MSM as “difficult” patients^46^ or staff explaining that they did not “condone” homosexuality^80^. Inadequate training/clinical supervision of staff around LGBTQ+ issues, or staff holding inaccurate information/myths/stereotypes about LGBTQ+ people (for example, that homosexuality is caused by sexual abuse^80^), was reported by nine studies^46,48,68,69,74,80,81,91,104^. One study reported that drug services having a ‘poor reputation’ was a potential barrier^66^.

Six studies described that disclosure of SM status whilst accessing or already in treatment negatively affected subsequent service provision^48,54,67,69,75,80^. Other barriers included heteronormative assumptions made on intake forms and during treatment (e.g. assuming that a woman must have a husband, or only talking about heterosexual relationships in treatment groups)^48,69,73,80,93^. Three studies reported that services had a lack of insight into the unique needs of SM mothers^48,73,93^, and one study reported services not including the partners of gay/lesbian people in ‘family’ programmes^73^. Two studies reported that the abstinence-only focus of services was a barrier^46,67^, for example not taking into account the importance of sexualised drug use^46^. For people involved with chemsex, stigma around accessing needle exchanges and services (because they are associated with opiate drug users) was reported as a barrier^88^. One study also described organisational tensions between non-specialist and specialist LGBT programmes within the same service^53^.

#### 3.2.3 Availability and Accommodation

This dimension of access relates to factors such as geographic location, opening hours, appointment mechanisms, models of care, and staff capacity. Here, studies reported on specific barriers perceived by SM patients such as inconvenient opening times (e.g. only 9am-5pm)^42,66,88,90^, geographical unavailability of services (e.g. outside main city areas)^56,66,78,94^, and problems with childcare provision at services^48,86^. Two studies reported waiting lists as a barrier (e.g. six months for a GBL/GHB detox)^66,101^, and two studies reported that low numbers of SM patients led to isolation within treatment programmes, or restricted provisions of SM-only services^53,54^. One study reported that the finite/limited resources of services were evident (e.g. not giving naloxone to non-injecting drug users)^79^. Staff feeling unable to accommodate the specific needs of SM patients^54^, and the type of treatment SM patients wanted not being offered^49^ were also reported as barriers. The effect of rurality vs. urbanicity on unmet alcohol and substance use disorder treatment need was examined by one paper, with mixed findings depending on different subgroups and no clear overall trend^62^.

#### 3.2.4 Affordability

Affordability-related barriers included health insurance not covering the cost of addiction treatment^49^, a lack of economic resources preventing access to appropriate addiction-related medical services^46^, low cost or free addiction treatment programmes not being available in certain regions^102^, and under-funded services being unable to pay for additional staff, rooms, or specialist training^53^.

#### 3.2.5 Appropriateness

The ‘appropriateness’ dimension of access relates to the fit between services and patients’ needs. Here, ten studies reported missed opportunities by services and staff to address or explore important LGBTQ+ issues (such as trauma, coming out, sexual health, sexualised drug use, and sexual violence)^48,54,57,67,69,80,81,91,94,101^. Several studies reported service staff were negative/awkward about, or lacking knowledge about, the topic of sexuality and sexual practices^48,54,57,69,74,81,86,91,93,94^ and that there was an inappropriate under-focus on sexuality (e.g. never mentioning it, or deflecting the topic even when patients explicitly linked substance use to the stresses associated with being SM)^48,54,69,80,86,94^. Some examples of specific negative experiences include: staff ‘outing’ patients without consent and breaching confidentiality^69,80^; instructing SM patients to hide their sexuality for the sake of non-SM patients in groups^48,54^; paternalistic, confrontational, or coercive consultation styles^48,69^; silencing patients who brought up chemsex-related topics^95^; staff stating that bisexuality did not exist^80^; staff not understanding the topic of sexual identity and motherhood^93^; and staff believing that sexuality-conversion therapy was effective or practicing conversion therapy to try and make patients heterosexual^54,80^. However, an inappropriate over-focus on sexuality could also be problematic, and make patients feel misunderstood or “different” (e.g. insisting homosexuality must be the cause of addiction problems)^48,54,69,80^. One study reported that services did not acknowledge or address intersectionality (e.g. race/ethnicity and sexuality)^48^, and one study reported that services were only targeting a middle-class clientele rather than patients with higher needs^43^.

### 3.3 Results: Service-Related Facilitators to Access

#### 3.3.1 Approachability

A variety of facilitators to access were reported. Firstly, multiple studies reported on the creation, provision, or importance of SM-specific services or treatment pathways, including specific needs assessments^53,54,67,69,73,76,78,86,88,93,94,99^. For existing services, several studies reported on outreach to the SM community to facilitate access (e.g. via smartphone hook-up apps, pop-up services, discussion groups, social networks, gay club nights/fetish venues, and commercial sex areas)^43,50,51,88,89,100^. Several studies mentioned clear referral pathways into addiction services (e.g. following crisis hospitalisation, from sexual health services, and from public assistance services)^43,88,100,101^, and for the provision of referral pathway options beyond the criminal justice system^88^. Six studies mentioned the creation of visibly LGBTQ+ safe/friendly environments (e.g. having LGBTQ+ positive waiting-room literature, or displaying Pride flags or rainbow stickers)^54,69,76,85,91,93^. Some study participants reported that they would prefer to access specialist drug services within sexual health clinics (e.g. because they were trusted as gay-friendly)^87,89^, or endorsed the idea of holistic ‘one-stop shop’ services which could address addiction, sexual health, and mental health concerns in an integrated way^81^. One study advocated for training medical staff (particularly in sexual health and HIV care) in identification and referral of chemsex participants into services^88^. Three studies recommended or reported on addiction service staff having knowledge of other local support services to facilitate onward referrals^76,81,91^. Partnership working (e.g. between addiction, sexual health, and emergency services, criminal justice agencies, housing providers, and LGBTQ+ organisations) is one way to achieve this^82,88,100^. Publicity around drug support services directed at MSM was mentioned by one study^89^, and one study on pharmacotherapy for alcohol use disorder (AUD) recommended improved information provision around this using the language of harm reduction^50^.

#### 3.3.2 Acceptability

Seeing an openly LGBTQ+ healthcare professional, managerial and organisational support of diversity within drug services, and services recruiting LGBTQ+ staff^47,53,54,69,73,74,76,82,91,94^ were reported facilitators that enhance the acceptability of services. Fourteen studies mentioned the importance of positive, caring, affirmative, or non-judgemental attitudes from service providers (e.g. staff empathising with the difficulties of homophobia, taking time to ensure patients felt comfortable, being able to openly discuss gay sexuality, or acting as LGBTQ+ allies)^54,69,73,75,76,81,82,85,87,89,90,94,100,102^, which can be facilitated through staff training (including for non-clinical staff) and policies about SM patients^54,69,73,93^. Three studies reported on the importance of ‘cultural humility’ or ‘horizontal approaches’ from service providers, involving critical self-reflection and positioning the patient as the expert in their lives^69,81,90^. Two studies recommended intake forms/processes using inclusive, open-ended language regarding sexuality and gender, and allowing non-disclosure of SM status^69,93^. Services that can be used anonymously may facilitate access in countries with sanctions around drug use or SM status^96^. Two studies also reported on staff at needle exchange programmes providing informal referrals to less LGBT-hostile service environments, or acting as sources of solidarity^53,69^. One study recommended the inclusion of LGBTQ+ people in service development (e.g. formulating non-discrimination or staff vetting policies)^69^, and another recommended staff explicitly let patients know that their service is LGB-positive at their first contact^85^. Positive LGBTQ+ community feedback can enhance the reputability of services^94^.

#### 3.3.3 Availability and Accommodation

Practical measures such as the establishment of evening and weekend drug clinics, offering alcohol treatment groups in non-clinical settings, and improved service provision in city centres^55,88^ were reported. Three studies also reported on locating services strategically in LGBTQ+ community areas or areas of high need (e.g. a needle exchange in the gay village, drop-in recovery services near where alcohol is sold)^55,88,89^. For existing services, gender neutral bathrooms may be a facilitator to access^53^. One study on factors associated with high access to substance abuse treatment programmes for MSM found these typically correlated with high access to HIV risk reduction and education; medical care; mental health services; and a high education level^102^, which can all function as facilitators. When asked to rate the relative importance of drug service design characteristics, the availability of one-to-one support in addition to counsellor-led group counselling; long-term, open-ended support; and support located close to home were rated highly by methamphetamine-using MSM^82^. One study noted the importance of having a separate treatment facility or separate unit/area within a service for SM patients^76^.

#### 3.3.4 Affordability

One study reported on drop-in community recovery centres offering cheap or sliding scale payments as a facilitator to access^55^.

#### 3.3.5 Appropriateness

Nine studies reported on the importance of holistic, intersectional, or ‘whole person’ approaches to treatment (e.g. incorporating sexuality, substance abuse, housing and social support, subsistence needs, mental health, culture, and spirituality)^48,54,57,69,73,76,81,82,90^ to enhance the appropriateness of services. Providers “meeting the client where they are at” (e.g. reflecting patients’ own language and identity labels, or offering flexibly tailored treatment)^45,69,76^ can also help, in addition to professionals having specific knowledge, education, and ‘cultural competence’ about issues relevant to SM healthcare (e.g. methamphetamine use, coming out challenges, societal homophobia, links between drug use and sexual practices)^46,53,54,57,69,73,76,78,81,82,85,94^. Open, non-judgemental questioning about gender and sexual preference during intake processes for new patients was reported as a facilitator^53,54,69,73,93^, with service providers acknowledging sexuality as an important component of identity whilst not assuming it is the cause of a patient’s problems^69,73,76,93^. Matching patients to therapists with their preferred sexual and/or gender identity^53^, and staff using a warm, hopeful consultation style which preserves the patient’s decision-making power^48,76,81^ were also reported facilitators. Two studies mentioned the importance of approaches which are trauma-informed and sex-positive^57,69^, one mentioned services being inclusive of HIV education and advocacy^57^, and two others reported on the ability to link patients to wider support programmes (e.g. a SM women’s group)^53,81^ as facilitators to access. Provision of relevant harm reduction supplies (e.g. clean needles) can also be a facilitator^69,82,100^.

### 3.4 Results: Patient-Related Barriers to Access

#### 3.4.1 Ability to Perceive

Complementary to the dimension of access of approachability, ‘ability to perceive’ includes patient-related factors such as health literacy, health beliefs, trust, and expectations. Included studies reported barriers such as ambivalence around drug/alcohol use or reluctance to change^42,47,50,58,67,98,101^, not seeing drug/alcohol use as a problem or serious problem^42,79,89,94,98,101^, and negative beliefs around addiction or its treatment (e.g. feeling ‘I should be strong enough to handle this alone’, not believing that ‘alcoholism’ is a treatable condition, or feeling that GBL/GHB users were treated inadequately by services)^42,66,97,98,101^. Several studies reported that patients did not know that addiction treatment was available/existed^42,50,66,79,94,97^, did not know where or how to access services^49,66,79,98^, were not sure if they would be eligible for treatment^66^, or felt that the type of treatment they wanted was not offered from services^49^. Two studies reported participants not believing addiction treatment was appropriate to their needs^48,79^, and three studies reported on concerns about other drug users, or rehabilitation itself, triggering relapse^67,79,98^. One study of MSM noted that greater frequency of methamphetamine use, and greater frequency of sexualised use, was associated with an increased perceived difficulty of accessing support^84^. Normalisation of drug/alcohol use among SM people/peers can also be a barrier to individuals perceiving the need to access services^101^. For lesbians specifically, a sense of being ‘different and isolated’ can lead to them feeling that conventional healthcare is not designed for their needs^48^.

#### 3.4.2 Ability to Seek

Studies reported barriers such previous unsuccessful attempts to access or utilise treatment^42,98,101^, fears or embarrassment about discussing addiction or treatment^42,66,73^, apprehension about discussing sexualised drug or alcohol use^75,79,87,89^, and fears of others’ opinions on learning about a person’s addiction problems^42,73^. Fear of stigma due to SM status, alcohol/drug use (particularly injecting), or addiction treatment itself was also reported^48,50,56,69,70,73,75,76,79,85,86,94–96^. People are worried about involuntary admission to hospital if services are accessed^42^, and for lesbian women particularly, there are fears that children will be taken into care if they enter treatment services^48^. Three studies reported on concerns that being given pathologising labels (e.g. ‘alcoholic’), might compound the social stigma already experienced by SM patients^45,48,86^. For Black non-gay identified men who have sex with men and women, there may be specific barriers to seeking treatment related to cultural constructions of masculinity^45^. One study described barriers to methamphetamine treatment for Black MSM as a racialised problem: caused by exposure to poverty, inadequate education, and few resources compared with white MSM^68^.

Women experiencing lifetime high risk drug use were more likely than moderate or low risk users to report that they had wanted but had not received professional support^44^. For services that are difficult to physically access, an increased commitment is needed to attend^56^. In the Singaporean context, fear of incarceration/criminal sanctions for drug use or gay sex, or being reported by staff to drug law enforcement agencies, were barriers to service access^95,96^. One USA study also reported not accessing services due to fears of legal ramifications^94^. There were also several studies reporting on differences in barriers to access comparing women vs. men, or SM subgroups. Compared with gay men, bisexual men were reported to have both a decreased^63,65^ and increased^83^ likelihood of drug treatment utilisation, and an increased likelihood of alcohol treatment utilisation^71^. Compared with a matched cohort of heterosexual men, SM men ≥50 years old had a lower rate of inpatient drug treatment utilisation, and compared with heterosexual women, SM women ≥50 years old had a higher rate of inpatient drug treatment utilisation^72^. Black bisexual women had higher odds of reporting a treatment gap for both specialty drug, and alcohol services (compared with Black bisexual men, gay men, or lesbian women), and Black gay men had worse odds of experiencing a drug specialty treatment gap than Black bisexual men^59^. Latina SM women were less likely to access substance use treatment services compared with white SM women^77^. Compared with SM men, SM women with probable AUD were less likely to have this diagnosed by a professional, and less likely to attend substance use-related appointments^60^. For gay/lesbian-specific treatment units, a focus on white men can mean that they are perceived as less welcoming for women and non-white people^76^.

#### 3.4.3 Ability to Reach

One study reported that Black lesbian and bisexual women had less social support to help with accessing alcohol treatment than for heterosexual women^52^, and another study noted that services may only be accessible by car, meaning that patients may need to pay others to drive them^56^. One study of MSM reported that family members could be a barrier to treatment entry, for example by trying to force them to do so, creating resistance to the idea^67^.

#### 3.4.4 Ability to Pay

Included studies reported barriers such as SM patients being unable to afford medical bills/pay for addiction services, or concerns about the potential costs^42,46,56,66^. For some specific treatments, such as medications for AUD, there may be concerns around cost to benefits ratio of medications^50^. Two USA studies also noted problems with obtaining health insurance, which may be dependent on employment^56,78^.

#### 3.4.5 Ability to Engage

Studies reported factors such as SM patients not wanting to engage with services, or wanting to continue alcohol/drug use^42^, not having time to attend a service^66^, difficulty in keeping appointments during periods of heavy substance use^56^, and difficulty staying sober because of being unable to enjoy sex without drugs^46^. Feelings of isolation or alienation (i.e. as a minority within predominantly heterosexual) services or therapeutic groups limited their success^67,75,76^. For specific treatments, such as medication for AUD, studies reported concerns around the burden of treatment, and fears that it might negatively impact enjoyment of alcohol^50,97^. Three studies reported concerns with confidentiality when accessing services^66,73,94^. Mistrust in healthcare providers was reported (e.g. patients feeling that healthcare providers have negative/pathologising attitudes or inadequate knowledge, or they have to educate their healthcare providers about LGBTQ+ related issues)^48,69,85,94^. Perhaps consequently, SM people feel the need to present in a heteronormative manner^80^, or censor/not disclose their sexuality^75,85,94^. This led to difficulties discussing sexual practices or identity in therapeutic relationships, or fearing that staff members cannot ‘relate’ to SM patients^54,69,75,85,89,94^. Drug and alcohol treatment may be unsuccessful or less successful if someone feels unable to disclose their sexuality or sexual practices^75,85,94^. Patients fearing homophobia, shaming, rejection, or abuse based on their sexual identity within treatment settings is a significant barrier to engagement^54,69,75,76,86,94^.

### 3.5 Results: Patient-Related Facilitators to Access

#### Ability to Perceive

Patient-related facilitators to access included knowledge of addiction support services and treatment options (e.g. pharmacological treatment of AUD)^50,101^, and peer advocates and LGBTQ+ networks helping patients to understand their addiction problems and promoting treatment^43,58,67^. Patients involved in chemsex were motivated to access services when they perceived it to be detrimental, or to avoid related financial, housing, or employment crises^94^.

#### Ability to Seek

No reported facilitators.

#### Ability to Reach

Signposting and referral to services through LGBTQ+ community contacts, networks, peer advocates, family and friends can act as facilitators to access^43,58,67,101^ and at least for MSM, use of substance abuse treatment programmes is positively associated with connection to the gay community^102^. Family support was viewed as important for successful treatment^97^, and gay/lesbian people being able to bring ‘whoever you want to be considered family’ into family meetings as part of treatment was a facilitator^73^. SM individuals with a history of criminal legal system involvement were also more likely to utilise drug treatment than SM individuals without this history^64^.

#### Ability to Pay

One study noted that providing AUD treatment for a nominal fee seems to allow for more buy-in with the idea of treatment^97^. A Canadian study reported on patients using private services (paid for directly or by insurance) even when free public healthcare was available, because of faster access or needing expertise not available in the public health sector^81^. For MSM, it was important that other participants in drug treatment be in similar financial situations to themselves^82^. One USA study reported that state addiction treatment funding could be used to access an LGBTQ-specific treatment centre^78^.

#### Ability to Engage

For SM women, one study reported that having specialist women-only services is associated with greater likelihood of completing treatment^92^. In addition, having specialist female-only or lesbian-only groups allows SM women to discuss specific relevant issues (e.g. sexual harassment and gender roles)^55,86^. For gay and lesbian patients, being able to ‘work though shame to self-acceptance’ was a facilitator to engagement and successful treatment^73^. Five studies reported on the focus of treatment being use reduction or harm reduction rather than abstinence, or allowing patients to set their own goals^55,67,69,82,97^, as a specific facilitator. For individuals with mild or moderate alcohol problems, they may be more likely to engage in a group in a non-clinical setting^55^, and peer support around GHB/GBL use was particularly helpful for MSM^101^. Building opportunities for socialising, self-expression, community connection, and mutual support (particularly with other SM people) within treatment facilitates engagement^67,73,75,76,82^. Two studies reported on the importance of SM role models who were in recovery from addiction themselves; both other patients and staff^73,94^. In one study, both SM and heterosexual participants reported that their top addiction recovery facilitators were friends, community and network; family and children; and 12-step programmes such as Alcoholics Anonymous. More SM than heterosexual participants cited spirituality, education, and loss (e.g. of identity, loved ones, agency) as facilitators to recovery^61^. In five studies, having other treatment participants who identify as SM or LGBTQ+ was helpful^69,73,75,76,82^. Three studies cited the importance of being able to be honest and open about the specific context of sexuality and addiction for treatment success^73,75,76^.

## 4 Discussion

### 4.1 Interpretation of Findings

This review highlighted a disparate range of potential, perceived, and enacted barriers and facilitators to accessing addiction services. As we hypothesised, both barriers specific to the SM community and generalised barriers were identified. Although not seeking to quantify the prevalence of different barriers/facilitators, nearly a third of included papers reported explicit physical and sexual violence, threats, abuse, and discrimination towards SM patients, or negative attitudes from staff members. These findings accord with a 2016 systematic review about LGBT access to health services^106^. Stressors such as these ‘get under the skin’ of SM people^16^, and may lead to identity concealment, expectations of rejection, hypervigilance to potential threats, and internalised stigma^15^. These in turn result in negative psychological and physical outcomes^107–112^. Discrimination is also likely to compound the effects of traumatic life events, which are experienced by SM people at higher rates^113,114^ and are associated with addiction^115^.

Abuse of patients in health settings is in stark opposition to principles of compassionate, equitable, and trauma-informed healthcare^116^. In this context, fearing stigma from services, as reported by fourteen of the included studies, may not be unjustified. These findings may reflect the fact that most of the included studies were conducted in the USA, where public attitudes towards SM may be more negative than in Western Europe^117^, and where there are variable (and worsening) legal and policy protections between states^118,119^. Despite reporting across five decades, findings from older papers are repeated in newer studies. For example, both a study from 1989^74^ and one from 2024^69^ reported treatment staff lacking specific LGBTQ+ expertise, and highlighted the benefits of having openly LGBTQ+ staff; and papers reporting on explicit harassment, discrimination, bullying, threats, violence, or abuse towards SM patients had publication dates fairly evenly spread between 1994 and 2024. We could not identify clear temporal trends in identified barriers and facilitators.

The results of this review suggest that staff demonstrating caring, non-judgemental acceptance of SM patients may be the most helpful facilitator to service access. However, tackling homophobic/biphobic or prejudiced attitudes and behaviours may be a necessary first step. Evidence-based approaches to this include intergroup contact (e.g., a session for heterosexual staff run by LGBTQ+ people), experiential learning approaches, and exercises to encourage empathy and perspective-taking^120–122^. Even when healthcare workers are not prejudiced, they may have limited education on LGBTQ+-related issues^123,124^. There is evidence that ‘cultural competency’ training can improve knowledge, clinical and interpersonal skills, and attitudes towards LGBTQ+ people^125^.

Several studies reported ambivalence around or reluctance to address addiction problems, or that they were not perceived as a serious issue. This finding may speak to the relative importance and positive role of drug and alcohol use for the SM community. Historically, gay bars may have been the only safe spaces to meet, and drug/alcohol use may be linked to identity formation, community connection, sexual fulfilment, or active resistance to (heterosexual) norms^126–129^. The increased prevalence of addiction problems among SM people may also mean that these are ‘normalised’ within this community^130^. Treatments or public health interventions should acknowledge that reducing or discontinuing drugs/alcohol may therefore be more complex for SM compared to heterosexual patients.

### 4.2 Strengths and Limitations

The search criteria were deliberately broad to ensure maximal retrieval and inclusion of studies, and synthesising quantitative and qualitative findings is a strength. However, the vast majority of included studies are from high-income countries, limiting the generalisability of findings to low- and middle-income countries. Findings around affordability and the practical aspects of access also need to be contextualised within differing health systems, which vary widely between countries in terms of policy and financial support (e.g. from public health systems free at the point of access, to private or insurance-based systems). This review only includes studies published in English, and unpublished or grey-literature results were not included, limiting its comprehensiveness. There is a large, related body of literature around the attitudes of treatment providers towards SM patients^131^, which we did not include if these were not specifically conceptualised as barriers. It is likely that negative attitudes of addiction healthcare practitioners contribute to the negative experiences of SM people.

A further limitation may be varying technical or cultural terminologies around SM. For example, in non-Western countries, sexual and gender minorities may be considered less separately than in Western countries^132^, and included papers used differing definitions of SM (e.g. sexual behaviour vs. self-defined identity). This review did not explicitly address the experiences of gender minorities, although many of the identified studies included trans or other gender minority participants. We also chose to focus on specialist addiction services. SM people are likely to seek support with addiction problems from other services. These may include primary care, general mental health services, and third-sector or mutual aid organisations; experiences of accessing these were not addressed by this review, and may differ from our findings.

Of the included studies, around half reported entirely or mostly on men, around a quarter reported entirely or mostly on women, and around a quarter were a more evenly mixed sample. The majority of studies focused on gay and lesbian participants, with a smaller number of bisexual participants, and only a very few studies that explicitly included queer or ‘other’ non-heterosexual SM people. Of the articles that reported racial/ethnic identity, white participants were the largest group in the majority of studies. Individuals with multiple marginalised identities may face intersecting health and social challenges^133,134^, which may potentiate barriers to treatment, and so this is a limitation of the applicability of the published research.

### 4.3 Conclusion and Future Implications

Although there is significant between-country variation, people with addiction problems tend to be underserved by available treatment services^135^. Global attention to reducing treatment gaps in addiction problems may be expected to reduce generalised barriers, although it is still possible that for any given generalised barrier, its effect may be more significant on SM than on heterosexual patients due to their increased marginalisation. SM people are also a heterogenous group with diverse needs, and differing access barriers and facilitators for different sub-groups (e.g. gay men vs. queer women). The findings imply a range of potential solutions to tackle the identified barriers. These vary from simple interventions (e.g. creating a visibly LGBTQ+ welcoming environment, staff training to address knowledge and attitudes), to more complex organisational interventions (e.g. establishing SM-specific services, or service provider competency frameworks), to wider policy and social changes (e.g. legal equalities protections for SM people, improved funding of public addiction services).

Future research, ideally using representative rather than convenience samples, may wish to focus on the relative importance of different identified barriers for SM individuals, and understand how these vary by orientation, gender identity, racial/ethnic identity, and country of origin. Implementation or intervention studies may wish to identify what works in practice to address barriers and facilitate access, with attention to local cultural and healthcare contexts.

## Supplementary Material

Online Supplementary Material

## Data Availability

Search syntaxes are provided in the online supplementary material.
