# Supplementary material for: Barriers and Facilitators to Accessing Addiction Treatment Services for Sexual Minority (LGBQ+) People: A Systematic Review
Source: Br J Psychiatry. Author manuscript; Available in PMC 2026 Jun 15. (PMC7619179; doi:10.1192/bjp.2026.10589)
Supplement: Online Supplementary Material [file EMS212401-supplement-Online_Supplementary_Material.pdf]

Online Supplementary Material

Online Supplementary Material....1
Supplementary Table 1: Inclusion and Exclusion Criteria....1
Supplementary Table 2: Search Syntaxes....2
Supplementary Table 3: Data Items Extracted....5
Supplementary Table 4: Included Studies....6
Supplementary Table 5: MMAT-2018 Responses for Included Studies....20
Supplementary Table 6: Excluded Studies....24
Supplementary Table 7: Summary Table of Results....38
Supplementary Table 8: PRISMA Checklist....45

Supplementary Table 1: Inclusion and Exclusion Criteria

	INCLUSION	EXCLUSION
**COVERAGE**	Articles reporting on adults (>80% of participants in study are ≥18 years)<br>AND<br>Studies published in English<br>AND<br>Published in any country	Articles reporting solely on patients <18 years<br>OR<br>Studies not published in English
**POPULATION**	Studies that report on the population defined above<br>OR<br>Studies that include findings from health and social work professionals, or service providers, involved in the care of the above population, which are relevant to the study aims	Studies that report only on heterosexual participants<br>OR<br>Studies where barriers/facilitators to treatment cannot be disaggregated for sexual minorities and heterosexual participants<br>OR<br>Studies reporting solely on gender minorities (e.g. trans or non-binary individuals)

**OUTCOMES**	Studies reporting on barriers to access for addiction treatment services (i.e. that are delivering specialist addiction-related healthcare such as medical assessment, pharmacotherapy, psychological support, detox, needle exchange, facilitated treatment groups) for the above population<br>OR<br>Studies reporting on facilitators to access for addiction treatment services for the above population	Studies reporting only on barriers/facilitators to access for peer-support or community-organised groups such as Alcoholics Anonymous<br>OR<br>Studies reporting on access barriers/facilitators to general mental health or medical services or primary care services (not specifically addiction services)<br>OR<br>Studies based exclusively on clinical management or outcomes for this population
**STUDY DESIGN/TYPE**	Qualitative, quantitative, or mixed-methods primary research studies	Editorials, reviews, case reports, comment articles, news articles, books, or conference abstracts<br>OR<br>Papers reporting solely on methods/instruments, with no research findings relevant to the study question
**QUALITY**	Passed MMAT-2018 screening questions	Did not pass MMAT-2018 screening questions
**OTHER**	Full-text available	Full-text not available

Supplementary Table 2: Search Syntaxes

DATABASE	SEARCH SYNTAX OR STRATEGY
MEDLINE	((("gay"[Title/Abstract] OR "lesbian*"[Title/Abstract] OR "LGB"[Title/Abstract] OR "queer*"[Title/Abstract] OR "msm"[Title/Abstract] OR "wsw"[Title/Abstract] OR "homosex*"[Title/Abstract] OR "pansex*"[Title/Abstract] OR "GLB"[Title/Abstract] OR "sexual minorit*"[Title/Abstract] OR "same sex attract*"[Title/Abstract] OR "non heterosexual*"[Title/Abstract] OR "men who have sex with men"[Title/Abstract] OR "women who have sex with women"[Title/Abstract] OR ("homosexuality"[MeSH Terms] OR "bisexuality"[MeSH Terms])) AND ("addict*"[Title/Abstract] OR "alcohol*"[Title/Abstract] OR "ivdu*"[Title/Abstract] OR "heroin"[Title/Abstract] OR "opioid"[Title/Abstract] OR "cocaine"[Title/Abstract] OR "methamphetamine"[Title/Abstract] OR "MDMA"[Title/Abstract] OR "ecstasy"[Title/Abstract] OR "GHB"[Title/Abstract] OR "GBL"[Title/Abstract] OR "hallucinogen*"[Title/Abstract] OR "cannabis"[Title/Abstract] OR "marijuana"[Title/Abstract] OR "drinking"[Title/Abstract] OR "injecting"[Title/Abstract] OR "substance misuse"[Title/Abstract] OR "substance use"[Title/Abstract] OR "substance abuse"[Title/Abstract] OR "illicit drug"[Title/Abstract] OR "illegal drug"[Title/Abstract] OR "drug misuse"[Title/Abstract] OR "drug abuse"[Title/Abstract] OR "drug use"[Title/Abstract] OR ("alcoholism"[MeSH Terms] OR "substance related disorders"[MeSH Terms] OR "drug seeking behavior"[MeSH Terms])) AND ("rehab*"[Title/Abstract] OR "service*"[Title/Abstract] OR "psychiatr*"[Title/Abstract] OR "CMHT"[Title/Abstract] OR "counsel*"[Title/Abstract] OR "GP"[Title/Abstract] OR "psychotherap*"[Title/Abstract] OR "detox*"[Title/Abstract] OR "treatment*"[Title/Abstract] OR "intervention*"[Title/Abstract] OR "therap*"[Title/Abstract] OR "mental health*"[Title/Abstract] OR "primary care"[Title/Abstract] OR "health team*"[Title/Abstract] OR ("substance abuse treatment centers"[MeSH Terms] OR "needle exchange programs"[MeSH Terms] OR "mental health services"[MeSH Terms] OR "addiction medicine"[MeSH Terms])) AND ("accepta*"[Title/Abstract] OR "access*"[Title/Abstract] OR

	"barrier*"[Title/Abstract] OR "challenge*"[Title/Abstract] OR "engage*"[Title/Abstract] OR "exclu*"[Title/Abstract] OR "hindrance*"[Title/Abstract] OR "assist*"[Title/Abstract] OR "limitation*"[Title/Abstract] OR "obstacle*"[Title/Abstract] OR "pathway*"[Title/Abstract] OR "utilisation*"[Title/Abstract] OR "utilization*"[Title/Abstract] OR "helpseeking"[Title/Abstract] OR "uptake*"[Title/Abstract] OR "facilitat*"[Title/Abstract] OR "furtherance"[Title/Abstract] OR "disparit*"[Title/Abstract] OR "treatment gap"[Title/Abstract] OR "help-seeking"[Title/Abstract] OR "help seeking"[Title/Abstract] OR "support-seeking"[Title/Abstract] OR "support seeking"[Title/Abstract] OR "seeking help"[Title/Abstract] OR "seeking support"[Title/Abstract] OR ("patient acceptance of health care"[MeSH Terms] OR "health services accessibility"[MeSH Terms] OR "help seeking behavior"[MeSH Terms])) AND "English"[Language] AND "humans"[MeSH Terms]) NOT ("Comment"[Publication Type] OR "Editorial"[Publication Type] OR "meta analysis"[Publication Type] OR "News"[Publication Type] OR "Review"[Publication Type] OR "Systematic Review"[Publication Type] OR "cochrane"[Title/Abstract] OR "literature review"[Title] OR "Systematic Review"[Title])) AND (alladult[Filter])
PSYCHInfo (via Ovid)	#1 gay.ti,ab. or lesbian*.ti,ab. or LGB.ti,ab. or queer*.ti,ab. or msm.ti,ab. or wsw.ti,ab. or homosex*.ti,ab. or pansex*.ti,ab. or GLB.ti,ab. or sexual minorit*.ti,ab. or same sex attract*.ti,ab. or non heterosexual*.ti,ab. or men who have sex with men.ti,ab. or women who have sex with women.ti,ab. or (exp homosexuality/ or exp bisexuality/)<br><br>#2 addict*.ti,ab. or alcohol*.ti,ab. or ivdu*.ti,ab. or heroin.ti,ab. or opioid.ti,ab. or cocaine.ti,ab. or methamphetamine.ti,ab. or MDMA.ti,ab. or ecstasy.ti,ab. or GHB.ti,ab. or GBL.ti,ab. or hallucinogen*.ti,ab. or cannabis.ti,ab. or marijuana.ti,ab. or drinking.ti,ab. or injecting.ti,ab. or substance misuse.ti,ab. or substance use.ti,ab. or substance abuse.ti,ab. or illicit drug.ti,ab. or illegal drug.ti,ab. or drug misuse.ti,ab. or drug abuse.ti,ab. or drug use.ti,ab. or (exp alcoholism/ or exp substance related disorders/ or exp drug seeking behavior/)<br><br>#3 rehab*.ti,ab. or service*.ti,ab. or psychiatr*.ti,ab. or CMHT.ti,ab. or counsel*.ti,ab. or GP.ti,ab. or psychotherap*.ti,ab. or detox*.ti,ab. or treatment*.ti,ab. or intervention*.ti,ab. or therap*.ti,ab. or mental health*.ti,ab. or primary care.ti,ab. or health team*.ti,ab. or (exp substance abuse treatment centers/ or exp needle exchange programs/ or exp mental health services/ or exp addiction medicine/)<br><br>#4 accepta*.ti,ab. or access*.ti,ab. or barrier*.ti,ab. or challenge*.ti,ab. or engage*.ti,ab. or exclu*.ti,ab. or hindrance*.ti,ab. or assist*.ti,ab. or limitation*.ti,ab. or obstacle*.ti,ab. or pathway*.ti,ab. or utilisation*.ti,ab. or utilization*.ti,ab. or helpseeking.ti,ab. or uptake*.ti,ab. or facilitat*.ti,ab. or furtherance.ti,ab. or disparit*.ti,ab. or treatment gap.ti,ab. or help-seeking.ti,ab. or help seeking.ti,ab. or support-seeking.ti,ab. or support seeking.ti,ab. or seeking help.ti,ab. or seeking support.ti,ab. or (exp patient acceptance of health care/ or exp health services accessibility/ or exp help seeking behavior/)<br><br>#5 exp Comment/ or exp Editorial/ or exp meta analysis/ or exp News/ or exp Review/ or exp Systematic Review/ or cochrane.ti,ab. or literature review.ti,bt. or Systematic Review.ti,bt.<br><br>(1 AND 2 AND 3 AND 4) NOT 5 with limits set of ‘English’, ‘Adult’
CINAHL (via EBSCO*Host*)	#1 TI (gay or lesbian* or LGB or queer* or msm or wsw or homosex* or pansex* or GLB or "sexual minorit*" or "same sex attract*" or "non heterosexual" or "men who have sex with men" or "women who have sex with women") or AB (gay or lesbian* or LGB or queer* or msm or wsw or

	homosex* or pansex* or GLB or "sexual minorit*" or "same sex attract*" or "non heterosexual" or "men who have sex with men" or "women who have sex with women") or (MH "Gay Persons+") or (MM "Bisexuals") or (MM "Gay Men") or (MM "Bisexuality")<br><br>#2 TI (addict* or alcohol* or ivdu* or heroin or opioid or cocaine or methamphetamine or MDMA or ecstasy or GHB or GBL or hallucinogen* or cannabis or marijuana or drinking or injecting or "substance misuse" or "substance use" or "substance abuse" or "illicit drug" or "illegal drug" or "drug misuse" or "drug abuse" or "drug use") or AB (addict* or alcohol* or ivdu* or heroin or opioid or cocaine or methamphetamine or MDMA or ecstasy or GHB or GBL or hallucinogen* or cannabis or marijuana or drinking or injecting or "substance misuse" or "substance use" or "substance abuse" or "illicit drug" or "illegal drug" or "drug misuse" or "drug abuse" or "drug use") or (MH "Substance Use Disorders+") or (MH "Alcohol Abuse+") or (MH "Alcoholism") or (MH "Drug-Seeking Behavior")<br><br>#3 TI (rehab* or service* or psychiatr* or CMHT or counsel* or GP or psychotherap* or detox* or treatment* or intervention* or therap* or "mental health*" or "primary care" or "health team*" or "needle exchange*" or AB (rehab* or service* or psychiatr* or CMHT or counsel* or GP or psychotherap* or detox* or treatment* or intervention* or therap* or "mental health*" or "primary care" or "health team*" or "needle exchange*") or (MH "Substance Use Rehabilitation Programs+") or (MM "Needle Exchange Programs") or (MH "Mental Health Services+") or (MM "Addictions Nursing")<br><br>#4 TI (accepta* or access* or barrier* or challenge* or engage* or exclu* or hindrance* or assist* or limitation* or obstacle* or pathway* or utilisation* or utilization* or helpseeking or uptake* or facilitat* or furtherance or disparit* or "treatment gap" or "help-seeking" or "help seeking" or "support-seeking" or "support seeking" or "seeking help" or "seeking support") or AB (accepta* or access* or barrier* or challenge* or engage* or exclu* or hindrance* or assist* or limitation* or obstacle* or pathway* or utilisation* or utilization* or helpseeking or uptake* or facilitat* or furtherance or disparit* or "treatment gap" or "help-seeking" or "help seeking" or "support-seeking" or "support seeking" or "seeking help" or "seeking support") or (MH "Health Services Accessibility+") or (MM "Help Seeking Behavior")<br><br>#5 TI (Cochrane or "literature review" or "systematic review" or "meta analysis") or AB (Cochrane or "literature review" or "systematic review" or "meta analysis")<br><br>(1 AND 2 AND 3 AND 4) NOT 5 with limits set of 'English', 'Humans', 'All Adult', 'Journal Article'
Web of Science	(((TI=(gay or lesbian* or LGB or queer* or msm or wsw or homosex* or pansex* or GLB or "sexual minorit*" or "same sex attract*" or "non heterosexual" or "men who have sex with men" or "women who have sex with women")) or (AB=(gay or lesbian* or LGB or queer* or msm or wsw or homosex* or pansex* or GLB or "sexual minorit*" or "same sex attract*" or "non heterosexual" or "men who have sex with men" or "women who have sex with women"))) AND ((TI=(addict* or alcohol* or ivdu* or heroin or opioid or cocaine or methamphetamine or MDMA or ecstasy or GHB or GBL or hallucinogen* or cannabis or marijuana or drinking or injecting or "substance misuse" or "substance use" or "substance abuse" or "illicit drug" or "illegal drug" or "drug misuse" or "drug abuse" or "drug use")) or (AB=(addict* or alcohol* or ivdu* or heroin or opioid or cocaine or methamphetamine or MDMA or ecstasy or GHB or GBL or hallucinogen* or cannabis or marijuana or drinking or injecting or "substance misuse" or "substance use" or "substance abuse" or "illicit drug" or "illegal drug" or "drug misuse" or "drug abuse" or "drug use"))) AND ((TI=(rehab* or service* or psychiatr* or CMHT or counsel* or GP or psychotherap* or detox* or treatment* or intervention* or therap* or "mental health*" or "primary care"

	or "health team*" or "needle exchange*")) or (AB=(rehab* or service* or psychiatr* or CMHT or counsel* or GP or psychotherap* or detox* or treatment* or intervention* or therap* or "mental health*" or "primary care" or "health team*" or "needle exchange*"))) AND ((TI=(accepta* or access* or barrier* or challenge* or engage* or exclu* or hindrance* or assist* or limitation* or obstacle* or pathway* or utilisation* or utilization* or helpseeking or uptake* or facilitat* or furtherance or disparit* or "treatment gap" or "help-seeking" or "help seeking" or "support-seeking" or "support seeking" or "seeking help" or "seeking support")) or (AB=(accepta* or access* or barrier* or challenge* or engage* or exclu* or hindrance* or assist* or limitation* or obstacle* or pathway* or utilisation* or utilization* or helpseeking or uptake* or facilitat* or furtherance or disparit* or "treatment gap" or "help-seeking" or "help seeking" or "support-seeking" or "support seeking" or "seeking help" or "seeking support")))) NOT ((TI=(Cochrane or "literature review" or "systematic review" or "meta analysis")) or (AB=(Cochrane or "literature review" or "systematic review" or "meta analysis"))) with limits set for 'English' and 'Journal Article'
Sociological Abstracts (via ProQuest)	#1 TITLE,ABSTRACT(gay or lesbian* or LGB or queer* or msm or wsw or homosex* or pansex* or GLB or "sexual minorit*" or "same sex attract*" or "non heterosexual" or "men who have sex with men" or "women who have sex with women") or MAINSUBJECT.EXACT.EXPLODE("Homosexuality") or MAINSUBJECT.EXACT.EXPLODE("Bisexuality")<br><br>#2 TITLE,ABSTRACT(addict* or alcohol* or ivdu* or heroin or opioid or cocaine or methamphetamine or MDMA or ecstasy or GHB or GBL or hallucinogen* or cannabis or marijuana or drinking or injecting or "substance misuse" or "substance use" or "substance abuse" or "illicit drug" or "illegal drug" or "drug misuse" or "drug abuse" or "drug use") or MAINSUBJECT.EXACT.EXPLODE("Drug Abuse") or MAINSUBJECT.EXACT.EXPLODE("Alcoholism") or MAINSUBJECT.EXACT.EXPLODE("Drug Addiction") or MAINSUBJECT.EXACT.EXPLODE("Substance Abuse")<br><br>#3 TITLE,ABSTRACT(rehab* or service* or psychiatr* or CMHT or counsel* or GP or psychotherap* or detox* or treatment* or intervention* or therap* or "mental health*" or "primary care" or "health team*" or "needle exchange*") or MAINSUBJECT.EXACT.EXPLODE("Needle Exchange Programs") or MAINSUBJECT.EXACT.EXPLODE("Mental Health Services")<br><br>#4 TITLE,ABSTRACT(accepta* or access* or barrier* or challenge* or engage* or exclu* or hindrance* or assist* or limitation* or obstacle* or pathway* or utilisation* or utilization* or helpseeking or uptake* or facilitat* or furtherance or disparit* or "treatment gap" or "help-seeking" or "help seeking" or "support-seeking" or "support seeking" or "seeking help" or "seeking support") or MAINSUBJECT.EXACT.EXPLODE("Help Seeking Behavior")<br><br>#5 TITLE,ABSTRACT(Cochrane or "literature review" or "systematic review" or "meta analysis")<br><br>(#1 AND #2 AND #3 AND #4) NOT #5 with limits set of 'Article' and 'English'

Supplementary Table 3: Data Items Extracted

The following key variables were extracted from identified full-text studies into Microsoft Excel using a standardised form:

Data Item	Description
Study identifier	First author and year of publication, study title
Type of study	Qualitative, quantitative, or mixed-methods
Country/countries that study is reporting data from	Name of country
Research methods and objectives of the study	Brief summary description of study indicating key objectives and methods
Total number of study patients/participants reported on	Number
Type of addiction treatment service reported on (e.g. statutory health services, private health services, third-sector providers, residential vs. non-residential settings)	Description of type of service
Barriers to access	Categorised into the dimensions of accessibility according to the model from Levesque et al., 'service side' factors (approachability, acceptability, availability & accommodation, affordability, appropriateness) and 'patient side' factors (ability to perceive, ability to seek, ability to reach, ability to pay, ability to engage)
Facilitators to access	Categorised into the dimensions of accessibility according to the model from Levesque et al., 'service side' factors (approachability, acceptability, availability & accommodation, affordability, appropriateness) and 'patient side' factors (ability to perceive, ability to seek, ability to reach, ability to pay, ability to engage)
(If data available) mean age of sample	Years
(If data available) numbers of gay vs. lesbian vs. bisexual vs. other SM participants in the sample	Reported using language of each paper
(If data available) gender identity of participants	Reported using language of each paper
(If data available) ethnic/racial identity of participants	Reported using language of each paper

Supplementary Table 4: Included Studies

**First author and year of publication**	**Full title**	**Country the data are from**	**Overall number of participants the study**	**Number of sexual minority participants in the study relevant to questions of the review**	**Study description and methodology**	**Type of addiction treatment service reported on (e.g. statutory health services, private health services, third-sector providers, residential vs. non-residential settings)**	**Mean age of SM people within sample (years)**	**Sexual identity of SM people in sample (number of participants)**	**Gender identity of SM people in sample (number of participants)**	**Race/ethnicity of SM people within sample (number of participants)**
Allen (2016)	Sexual orientation, treatment utilization, and barriers for alcohol related problems: Findings from a nationally	USA	11,182	53	Secondary analysis of representative population survey (NESARC) on alcohol-related conditions, comparing heterosexual and sexual minority respondents' ratings of	Alcohol or drug detox, 12-step meetings including AA/NA, family/social service, inpatient wards, outpatient clinics, rehab programmes, A&E, crisis	Not reported	Gay or lesbian 31, bisexual 22	Gay/lesbian: 60.3% male and 39.7% female<br><br>Bisexual: 32.3% male and 67.7% female	Gay/lesbian: White non-Hispanic 73.2%, Black/African American 6.9%, American Indian/Alaskan

	representative sample				barriers to treatment access	centres, employee assistance programmes, clergyman/priest/rabbi, private professional, other agencies				Native 5.7%, Asian/Pacific Islander 3.5%, Hispanic (any race) 10.8%<br><br>Bisexual: White non-Hispanic 76.8%, Black/African American 12.3%, American Indian/Alaskan Native 2.0%, Asian/Pacific Islander 0.9%, Hispanic (any race) 7.9%
Aslan (2024)	Harm reduction and multidisciplinary consultations for gay, bisexual, and other men who have sex with men practising chemsex based in a French infectious disease unit: patients' characteristics and perceptions	France	172	96	Cross-sectional survey assessing sociodemographic and clinical characteristics of gay, bisexual, and other men who have sex with men (MSM) involved in chemsex who attended a specific chemsex harm reduction programme	Establishment of a specific chemsex treatment/harm reduction programme within an infectious diseases unit	41	Gay, bisexual, or other men who have sex with men 96	Men 96	Unknown
Barbara (2002)	Substance abuse treatment with lesbian, gay and bisexual people: A qualitative study of service providers	Canada	26	18 (study of service providers)	Qualitative study of service providers' knowledge, involving focus groups and individual interviews with clinicians experienced in treating LGB clients with substance abuse problems	All participants in the study delivering LGB substance abuse treatment via addiction centres, counselling centres or private practice	Not reported specifically for SM, of all participants: 43 (range 30-64)	Gay or lesbian 18	Not reported specifically for SM, of all participants: female 17, male 9	Unknown
Batchelder (2021)	Mental Health and Substance Use Diagnoses and Treatment Disparities by Sexual Orientation and Gender in a Community Health Center Sample	USA	24,325	13,648	Quantitative analysis of data from electronic medical records at health centre for sexual and gender minority patients, comparing mental health and addiction diagnoses, and treatment attendance, between sexual orientation and gender categories	Substance abuse treatment services at a community health centre	32.1	Gay men 8652, gay/lesbian women 1718, bisexual men 771, bisexual women 1189, other men 640, other women 678	Cisgender men 9141, cisgender women 2745, transgender men 615, transgender women 742	Race: Native American, Pacific Islander or Other 382, Asian or Asian American 687, Black or African American 786, Multiracial 743, White 10,321<br><br>Ethnicity: Latinx 754
Bernier (2024)	Examining sexual minority engagement	USA	337	68	Mixed methods study: quantitative analysis comparing heterosexual	Addiction recovery community centres offering e.g. relapse	40.34	Non-sexual or asexual 7, homosexual, gay or lesbian 22, bisexual 30,	Cisgender male 24, cisgender female 41,	Race: White or Caucasian 49, Black or African

	in recovery community centers				and sexual minority participants' utilisation of addiction recovery community centres, qualitative analysis of recovery facilitators	prevention, case management, employment assistance, support groups		questioning, curious or not sure 8, other 3	transgender male 2, other 1	American 15, Native Indian or Alaskan Native 1<br><br>Ethnicity: Hispanic or Latino 9, Not Hispanic 59
Blanchette (2024)	Sexualized substance use among gbMSM: Their perspectives on their intervention needs and counsellor competencies	Canada	20	20	Qualitative semi-structured interviews with gay, bisexual, or other MSM with addiction treatment experience, aiming to identify sexualised substance use intervention needs, analysed using descriptive/interpretive approach within frameworks of sexual health and addiction counsellor competencies	Substance use treatment programs	37.6 (range 23-60)	Gay 17, bisexual 1, pansexual 1, queer 1	Cisgender male 19, trans male 1	Ethnocultural background: Canadian 18, Latino 2
Bourne (2015)	"Chemsex" and harm reduction need among gay men in South London	UK	30	30	Qualitative interviews with gay men involved in the chemsex scene in South London, analysed using thematic analysis	Specialist drug services	36 (range 21-53)	Gay men 30	Men 30	White British 16, White other 8, White Irish 3, Black Caribbean 1, Other 2
Braine (2014)	Sexual Minority Women Who Use Drugs: Prejudice, Poverty, and Access to Care	USA	34	34	Qualitative semi-structured interviews with sexual minority female heroin or crack cocaine users in New York City, analysed using grounded theory	Inpatient drug treatment programs, syringe/needle exchange programmes, methadone maintenance programmes	Not reported (range 20-60+)	Lesbian/gay 19, women who have sex with women (WSW) 5, bisexual 6, primarily heterosexual WSW 4	Female 34, of which 1 assigned male at birth<br><br>Gender conformity: 'normative gender identity' 23, 'non conforming gender identity' 34	African American 17, Latina (Puerto Rico and Dominican Republic) 5, White 7, Mixed 5
Brown (2017)	Men Who Have Sex With Men in Peru: Acceptability of Medication-Assisted Therapy for Treating Alcohol Use Disorders	Peru	26	20 (sexual identity of remaining participants unspecified)	Qualitative study using three focus groups about acceptability of medication assisted therapy for alcohol use disorder in Lima, analysed using thematic analysis	Medication assisted therapy for AUD	28.3 (range 20-40)	Male: homosexual 8, bisexual 5, heterosexual MSM 4, Unspecified 3<br><br>Transgender women: 6 (sexual identity unspecified)	Men 20, trans women 6	Unknown
Card (2021)	Perceived difficulty of getting help to reduce or abstain from substances among sexual and gender minority men	Canada	376	376	Quantitative online survey among methamphetamine-using SGMSM examining demographic, clinical, and social predictors of	Addiction treatment, counselling, or harm reduction services	42.00	Men, trans men, or non-binary people who have sex with men 376, of which non-gay identified 88	Cisgender men 352, trans or non-binary 24	White 248, Person of Colour 104

	who have sex with men (SGMSM) and use methamphetamine during the early period of the COVID-19 pandemic				perceived difficulty in getting help with substance use during the initial phases of the COVID-19 pandemic					
Card (2022)	Does Treatment Readiness Shape Service-Design Preferences of Gay, Bisexual, and Other Men Who Have Sex with Men Who Use Crystal Methamphetamine? A Cross Sectional Study	Canada	291	291	Cross-sectional survey of gay, bisexual, and other MSM who use methamphetamine, examining the association between readiness-to-change (assessed using SOCRATES-8D scale) and relative importance of different addiction service design characteristics	Study assessing theoretical addiction service design characteristics; participants asked about 'professional help to control, cut down, or stop using crystal methamphetamine'	Median 41 (Q1-Q3 34-51)	MSM 291, of which gay 231, bisexual 34, other 26	Cisgender men 263, trans or non-binary 22	Person of Colour 83, Not Person of Colour 208
Cochran (2007)	Substance abuse treatment providers' explicit and implicit attitudes regarding sexual minorities	USA	46	11	Survey of substance abuse treatment providers' implicit and explicit attitudes regarding LGBT clients (measures of heterosexist/homophobic bias), quantitative analysis of measures of implicit and explicit bias	All specialist substance abuse treatment counsellors	Not reported specifically for SM, of all participants: 46.3	Gay/lesbian 7, bisexual 3, other 1	Not reported specifically for SM, of all participants: 52% female	Not reported specifically for SM, of all participants: Caucasian 22, Latino/a 9, African American 5, Asian American 4, Multi-racial 4, Other 2
Conner (2023)	Access and Barriers to Health Services Among Sexual and Gender Minority College Students	USA	1892	406	Online survey comparing cisgender, heterosexual with sexual and gender minority college students, assessing demographics, mental health and addiction characteristics, service utilisation, and barriers to service access	Substance use services	Not reported, but majority (68.9%) of all participants in age range 18-20	Gay or lesbian 63, bisexual or pansexual 246, questioning or bicurious 54, asexual or different identity 43	Female sex assigned at birth 325	Minority race or ethnicity 91
Copeland (1992)	A comparison of women seeking drug and alcohol treatment in a specialist women's and two traditional mixed-sex treatment services	Australia	320	27 lesbian; bisexual women also included in study but numbers aggregated with heterosexual women	Characteristics of 160 women who left specialist addiction services <5 days from admission compared to 160 women who stayed in specialist addiction services 5 or more days from admission; specialist addiction services were comprised of a specialist women's centre with residential childcare and a traditional mixed sex treatment service	Specialist drug treatment services	Not reported specifically for SM, of all participants: 29.7 (left services) and 30.8 (continued in services), range 17-66	Lesbian 27; bisexual women also included in study but numbers aggregated with heterosexual women	Women 27	Unknown

Corliss (2006)	Drug use, drug severity, and help-seeking behaviors of lesbian and bisexual women	USA	2011	2011	Self-administered cross-sectional questionnaire of sexual minority women in the San Francisco Bay Area and Los Angeles County, assessing demographics, drug use information, social/emotional problems, and help-seeking behaviours	Professional substance use services compared to self-help services	Not reported (range 18-55+)	Lesbian 82.8%, bisexual 11.2%, other non-heterosexual 6.0%	Women 2011	White 66.7%, African American 10.2%, Latina 13.2%, Asian/Pacific Islander 6.7%, Other 3.1%
Dyar (2023)	Rural and urban differences in disparities in substance use and substance use disorders affecting sexual minority populations	USA	210392	15007	Quantitative analysis of data from 2015-2019 National Survey of Drug Use and Health (NSDUH), comparing sexual minority to heterosexual disparities in substance use, substance use disorders, and treatment need as a function of urbanicity/rurality	Substance use treatment or counselling (for alcohol and/or drugs)	Not reported	Lesbian female 2292, bisexual female 8017, gay male 2348, bisexual male 2350	Female 10309, male 4698	Lesbian female: Non-Hispanic White (NHW) 63.0%, Non-Hispanic Black (NHB) 15.7%, Hispanic (H) 15.0%, Non-Hispanic Asian (NHA) 2.7%, Non-Hispanic Other Race (NHOR) 3.6%<br><br>Bisexual female: NHW 60.8%, NHB 13.8%, H 6.2%, NHA 44.4%, NHOR 4.8%<br><br>Gay male: NHW 61.5%, NHB 11.3%, H 19.4%, NHA 4.6%, NHOR 3.1%<br><br>Bisexual male: NHW 59.8%, NHB 10.1%, H 20.0%, NHA 7.0%, NHOR 3.1%
Flores (2017)	Availability and Use of Substance Abuse Treatment Programs Among Substance-Using Men Who Have Sex With Men Worldwide	Global	3478	3478	Global, cross-sectional online survey of substance-using MSM aiming to explore substance abuse treatment programme availability and use, and behavioural and demographic correlates of treatment programme use	Free or low-cost substance abuse treatment programmes	35	MSM 3478	Men 3478	Minority racial/ethnic category within country in which they reside 891, not a minority racial/ethnic category within country in which they reside 2640

Forenza (2016)	Exploring Service Provider Perceptions of Treatment Barriers Facing Black, Non-Gay-Identified MSMW	USA	18	Not reported (study of service providers)	Qualitative study involving two focus groups in New Jersey and New York City of 18 individuals from HIV/AIDS and substance abuse service providers, focusing on Black Non-Gay-Identified men who have sex with men and women (NGI-MSMW), analysed using grounded theory methods	Outpatient counselling, residential treatment, harm reduction services, detox and medical treatment for substance use disorders	Not reported (study of service providers)	Not reported (study of service providers)	Not reported (study of service providers)	Not reported (study of service providers)
Freestone (2025)	Exploring experiences of talk therapies among gay and bisexual men seeking to reduce or abstain from using crystal methamphetamine in the context of chemsex	Australia	24	24	Qualitative interview study with gay and bisexual MSM and non-binary people with chemsex experience, focusing on access to counselling or therapy around drug use, analysed using qualitative descriptive methodology	Therapeutic and psychological support for drug use, including an outpatient hospital stimulant treatment programme, LGBTQ+ drug counselling programme, drug support from a psychologist or counsellor, drug counselling via sexual health clinics or LGBTQ+ organisations	Not reported	Gay 21, bisexual 3, queer 1	Cisgender male 22, trans male 1, non-binary person 1	Caucasian 21, Asian 3, Latinx 1
Goodyear (2020)	"Am I gonna get in trouble for acknowledging my will to be safe?": Identifying the experiences of young sexual minority men and substance use in the context of an opioid overdose crisis	Canada	50	50	Semi-structured interviews with 50 sexual minority men who use substances in Vancouver, Canada, explicitly exploring how patterns and contexts of substance use are linked to the opioid overdose crisis	Harm reduction and drug use treatment services	24.18 (range 18-30)	Gay 29, bisexual 14, pansexual 6, queer 4, straight 2, asexual 1, unsure 1, other 2<br><br>(Note some participants selected more than one sexual identity)	Cisgender man 44, transgender man 4, queer 4, genderqueer 2, non-binary 1<br><br>(Note some participants selected more than one gender identity)	White 26, Aboriginal 10, Latin American 7, South Asian 5, South East Asian 2, Chinese 2, Korean 2, Filipino 2, Arab/West Asian 2, Black 1, Japanese 1, Other 2<br><br>(Note some participants selected more than one racial identity)
Gorman (1997)	Speed, Sex, Gay Men, and HIV: Ecological and Community Perspectives	USA	30	30	Exploratory ethnographic study of MSM who use drugs in the Seattle area using ethnographic observations, focus groups and individual interviews	Drug treatment programme	Not reported (range 20-56)	MSM or trans women having sex with men 30, of which majority gay, about 1/6 bisexual, 3 trans women, some declined to specify sexual orientation	Not fully reported but all MSM or trans women	Approx 80% White, 20% included Native American, Latino, African American and Mixed-Race individuals
Graf (2018)	Chemsex among men who have sex with	Germany	116	89	Qualitative study of drug-using MSM and health	Multidisciplinary chemsex support	Not reported (range 22-64)	MSM 89	Men 89	Not specified, though study

	men in Germany: motives, consequences and the response of the support system				professionals' perspectives on chemsex, using individual interviews and analysed using content analysis	services (including medical care, psychosocial and drug advice, sexual therapy, inpatient withdrawal treatment, harm reduction)				notes 'nearly none of them [participants] had a migratory background'
Green (2011)	Barriers and treatment preferences reported by worried drinkers of various sexual orientations	USA	218	62	Online convenience survey of worried alcohol drinkers, comparing heterosexual vs. LGB respondents, asking about barriers to treatment access and addiction treatment preferences	Professional inpatient treatment, professional outpatient treatment, self-help groups, computerised treatment, self-help books	Not reported	Lesbian women 34, bisexual women 12, bisexual men 2, gay men 14	Male 16, female 46	Unknown
Hall (1994)	Lesbians recovering from alcohol problems: an ethnographic study of health care experiences	USA	35	35	Qualitative semi-structured ethnographic interviews with lesbian problem alcohol drinkers, analysed using grounded theory and critical feminist approaches	Outpatient specialist addiction services and barriers to accessing formal addiction treatments	37 (range 24-54)	Lesbian 35	Female 35	Euro-American 24, African American 6, Latina 3, Asian/Pacific 1, Native American 1
Haney (2021)	Sexual Orientation, Social Determinants of Health, and Unmet Substance Use Treatment Need: Findings from a National Survey	USA	988	175	Analysis of data from representative population survey (National Survey of Drug Use and Health) comparing heterosexual with gay/lesbian/bisexual respondents regarding perceived reasons for unmet substance use treatment need	Speciality treatment facilities for illicit drug/alcohol use, including inpatient hospitals, drug/alcohol rehabilitation facilities (in/outpatient) and mental health centres	Not reported	Lesbian, gay, bisexual 175 (no disaggregated data)	Unknown	Unknown
Hellman (1989)	Treatment of homosexual alcoholics in government-funded agencies: provider training and attitudes	USA	164	13 (study of service providers)	Descriptive quantitative survey of service providers in government-funded agencies in New York City, focusing on their training experiences, competencies, and attitudes towards treating homosexual alcoholics	Government-funded alcohol treatment agencies	Not reported specifically for SM, of all participants: mean not reported, age ranges 18-24 = 4, 25-34 = 39, 35-44 = 60, 45-54 = 42, 55-64 = 12, 65+ = 2	Bisexual 3, homosexual 10 (service providers)	Not reported specifically for SM, of all participants: male 64, female 94	Not reported specifically for SM, of all participants: Asian 6, Black 48, Hispanic 26, White 73, Other 3
Hodges (2023)	Sexual minorities and substance use treatment utilization: New evidence from a national sample	USA	21,926	2871	Quantitative study using data from the 2015-2019 National Survey on Drug Use and Health (NSDUH) to examine the association between sexual identity, gender, and substance use	Substance use disorder treatment for alcohol, illicit drugs, and prescription medication misuse (including residential,	Not reported in detail, though age included as potential confounder	Gay men 487, bisexual men 453, lesbian women 342, bisexual women 1589	Male 940, female 1931	Not reported in detail, though race/ethnicity included as potential confounder

					treatment utilisation, controlling for demographic covariates	inpatient, outpatient, and self-help groups)				
Hsiang (2018)	Acceptability of pharmacotherapy for hazardous alcohol use among men who have sex with men: Findings from a qualitative study	USA	39	39	Qualitative focus group discussions with MSM from San Francisco Bay area who had consumed alcohol in the last year, analysed using content analysis	Pharmacological treatment for hazardous drinking	39.1 (range 23-66)	MSM 39	Male 39	Asian/Pacific Islander 10, Black/African American 9, Latino 6, Mixed 4, White 10
Hussen (2021)	Exploring influences on methamphetamine use among Black gay, bisexual and other men who have sex with men in Atlanta: A focus group study	USA	54	36	Qualitative study using thematic analysis of eight focus groups of key informants from community-based and healthcare organisations working with Black gay, bisexual, and other MSM, exploring trends in methamphetamine use and the associated risk environment	Substance use treatment, including harm reduction facilities	Not reported specifically for SM, of all participants: mean not specified, age ranges 20-30 = 22, 31-40 = 15, 41-50 = 8, 51+ = 7<br><br>(Note two participants missing demographic data)	Gay/same-gender loving 29, bisexual 4, other 3	Not reported specifically for SM, of all participants: cisgender man 35, cisgender woman 14, transgender woman 2, gender non-conforming person 1<br><br>(Note two participants missing demographic data)	Not reported specifically for SM, of all participants: Black 45, White 3, Other 4<br><br>(Note two participants missing demographic data)
Israelstam (1988)	Knowledge and opinions of alcohol intervention workers in Ontario, Canada, regarding issues affecting male gays and lesbians: Parts I and II	Canada	85	Not reported (study of service providers)	Quantitative mailed, anonymous survey to assess the knowledge, attitudes, and experiences of alcohol intervention workers, with a focus on the treatment of gay and lesbian clients	Alcohol treatment or intervention services	Not reported specifically for SM, of all participants: median 40, range 25-63	Not reported	Not reported specifically for SM, of all participants: male 39, female 44, unknown 2	Not reported
Jeong (2016)	Racial/ethnic differences in unmet needs for mental health and substance use treatment in a community-based sample of sexual minority women	USA	699	699	Quantitative analysis of cross-sectional data from the Chicago Health and Life Experiences of Women (CHLEW) study, employing logistic regression to examine associations between sexual identity, race/ethnicity, and use of mental health and substance use treatment while controlling for various demographic factors	Substance use treatment	Not reported, however age ranges: ≤30 = 225, 31-40 = 149, 41-50 = 141, ≥51 = 184	Lesbian 517, bisexual 182	Women 699	Latina 162, White 261, African American 252

Joyce (2018)	The Experience and Meaning of Problematic 'G' (GHB/GBL) Use in an Irish Context: an Interpretative Phenomenological Analysis	Ireland	7	5	Qualitative semi-structured interviews with participants attending a community drug team for support with problematic GHB/GBL use, analysed using interpretative phenomenological analysis	Specialist drug service for problematic GHB/GBL use	36.2 (range 31-39)	Gay 5	Male 5	White 5
Kanouse (2005)	Recruiting drug-using men who have sex with men into behavioral interventions: a two-stage approach	USA	83	83	Quantitative study comparing methods of recruiting drug-using MSM into behavioural interventions (such as motivational interviewing or contingency management) - 2 stage (recruiting into discussion groups then offering intervention) vs. 1 stage (offering intervention)	Drug use behavioural interventions	Not reported	Gay 53, bisexual 25, other 5	Male 83	African-American 23, White 32, Hispanic 17, Asian/Pacific Islander 2, unspecified 9
Krasnova (2021)	Disparities in substance use disorder treatment use and perceived need by sexual identity and gender among adults in the United States	USA	21,926	2871	Quantitative study of data from the 2015-2019 National Survey on Drug Use and Health (NSDUH) using weighted multivariable logistic regression models to examine sexual orientation differences in treatment utilisation and perceived need for substance use treatment, adjusting for socio-demographic characteristics	Study examined both 'any substance use disorder (SUD) treatment' (any service utilisation in outpatient and inpatient settings, such as a hospital, rehabilitation facility, emergency room, mental health centre, doctor's office, prison or jail, self-help services such as Alcoholics Anonymous and Narcotics Anonymous, or "some other place") and 'specialty SUD treatment', (treatment in an inpatient hospital, drug or alcohol rehabilitation centre (inpatient or outpatient), or mental health centre offering specialty treatments)	Not reported	Gay men 487, bisexual men 453, gay/lesbian women 342, bisexual women 1589	Men 940, women 1931	Gay men: Non-Hispanic white (NHW) 59.2%, Non-Hispanic Black (NHB) 15.5%, Hispanic (H) 20.1%, Other (O) 5.3%<br><br>Bisexual men: NHW 63.3%, NHB 9.7%, H 20.6%, O 6.5%<br><br>Gay/lesbian women: NHW 57.1%, NHB 17.0%, H 18.2%, O 7.6%<br><br>Bisexual women: NHW 61.6%, NHB 15.1%, H 15.7%, O 7.7%
Matthews (2006)	Treatment Experiences of Gays and Lesbians In Recovery from Addiction: A Qualitative Inquiry	USA	10	10	Qualitative interview study of gay men and lesbians' experiences of treatment and recovery from drug and/or alcohol addiction,	Addiction treatment programs including inpatient, outpatient, partial hospitalisation, and 12-step programs	46.9 (range 31-69)	Lesbian/dyke 6, gay male/homosexual 4	Women 6, men 4	European American 9, African American 1

					analysed using thematic analysis					
Mays (1994)	Perceived social support for help-seeking behaviors of Black heterosexual and homosexually active women alcoholics	USA	70 (of whom 59 specified their sexuality)	21	Cross-sectional self-administered questionnaire of African American women in alcoholism treatment centres asking about social support and help seeking behaviours	Inpatient alcoholism treatment centres	31 (range 20-68)	Lesbian 12, bisexual 9	Female 21	African-American 21
McGaughey (2023)	Harm reduction services for people engaging in chemsex in Brighton, UK: A pilot qualitative study	UK	10 (service providers)	Not reported	Qualitative semi-structured interview study of commissioners, managers, or providers of chemsex harm reduction services, analysed using thematic analysis	Harm reduction services for chemsex	Not reported	Not reported	Not reported	Not reported
Mericle (2018)	Delivering LGBT-sensitive substance use treatment to sexual minority women	USA	11	Not reported (study of service providers)	Qualitative semi-structured elite interviews with directors of substance use treatment programmes in the San Francisco Bay area, focusing on how they address the needs of sexual minority women, analysed using qualitative framework approach	Residential and outpatient specialist substance use addiction services	Not reported (study of service providers)	Not reported (study of service providers)	Female 9, male 2	Not reported (study of service providers)
Micale (2024)	Examining alcohol use disorder treatment utilization in lesbian, gay, and bisexual populations through the Behavioral Model for Vulnerable Populations	USA	1152	1152	Quantitative analysis of data from 2012-2013 National Epidemiologic Survey on Alcohol and Related Conditions-III (NESARC-III), analysed using logistic regression based on the Behavioral Model for Vulnerable Populations, to assess the impact of predisposing factors, enabling factors, and treatment need on alcohol treatment use among LGB participants	Professional alcohol use disorder services (including family services or other social service agencies, alcohol/drug detoxification wards/clinics, inpatient wards of psychiatric/general hospital or community mental health programs, outpatient clinics, alcohol/drug rehabilitation programs, halfway houses/therapeutic communities, private physicians, psychiatrists, psychologists, or social workers, any other professional or agency)	37.56	Gay male 321, lesbian woman 265, bisexual man 144, bisexual woman 422	Female 687, male 465	White, non-Hispanic 622, Black, non-Hispanic 266, American Indian/Alaska Native, non-Hispanic 20, Asian/Native Hawaiian/Other Pacific Islander, non-Hispanic 28, Hispanic (any race) 216

Nieder (2025)	Minority Stress, Substance Use, and Resilience: Examining Long-Term Sobriety in the LGBTQ Community	USA	16	14	Qualitative interview study with LGBTQ people in long-term recovery (2-24 years) from substance use disorders, focusing on their paths to recovery, analysed using thematic analysis	Professional drug support services including inpatient and outpatient substance use programs, sober living programs, therapy, LGBTQ-specific drug treatment programs	35.38 (range 20-69)	Gay 7, lesbian 2, bisexual 1, pansexual 2, queer 2	Cisgender male 6, cisgender female 5, non-binary 2, genderfluid 1	White 7, Latinx 3, Black 1, Black/White 1, Latinx/White 1, Native American/White 1
Parry (2008)	Rapid assessment of drug-related HIV risk among men who have sex with men in three South African cities	South Africa	78	78	Qualitative study using rapid assessment methodology including mapping, observations, key informant interviews and focus groups with 78 drug-using MSM in Pretoria, Durban and Cape Town	Drug treatment centres and drug rehabilitation centres	For key informant interviews: 28.9, (range 18-50)<br>For focus group: not reported (range 19-55)	MSM 78	Men 78	African Black 13, Coloured 23, Indian 3, White 39
Paschen-Wolff (2024)	Experiences of and recommendations for LGBTQ plus - affirming substance use services: an exploratory qualitative descriptive study with LGBTQ plus people who use opioids and other drugs	USA	23	23	Qualitative study using individual interviews with LGBTQ+ people, exploring experiences of substance use treatments and generating recommendations for affirming care, analysed using thematic analysis	Substance use treatment services and related services (e.g. syringe exchange services and 12-step programs)	27.5 (range 21-38)	Bisexual 5, gay 4, lesbian 4, prefer not to specify 1, pansexual 2, queer 7	Cisgender man 4, cisgender woman 8, non-binary 4 (two of whom also identified as transgender), transgender man 6, transgender woman 1	Black/African American (non-Hispanic) 7, Latinx (any race) 4, Multiracial (non-Hispanic) 3, White (non-Hispanic) 9
Penn (2013)	LGBTQ persons with co-occurring conditions: Perspectives on treatment	USA	10	10	Qualitative semi-structured focus group exploring barriers, needs and experiences of LGBTQ people living with substance abuse and mental illness, analysed using grounded theory methods	Therapeutic programme to address substance abuse and mental health conditions	Not reported (range 22-51)	Lesbian 2, gay 3, bisexual 2, transgendered 1, lesbian/bisexual 1, gay/bisexual 1	Male 5, female 5	White 5, Hispanic 2, Native American 2, Black 1
Pennay (2018)	Improving alcohol and mental health treatment for lesbian, bisexual and queer women: Identity matters	Australia	25	25	Qualitative semi-structured interviews with sexual minority women about alcohol and mental health treatment, analysed thematically using a grounded theory approach	Alcohol and substance use treatment services	40.1 (range 19-71)	Lesbian 11, bisexual 5, gay 2, queer 2, pansexual 2, did not wish to be labelled 3	Female 22, transgender 1, intersex 1, genderqueer 1	Race/ethnicity not described but country of birth provided: Australia 18 (1 Aboriginal), UK 3, New Zealand 1, USA 1, Russia 1, Singapore 1
Ralphs (2018)	New psychoactive substances: new service provider challenges	UK	84	15 chemsex users - all assumed to be LGBTQ, no information given about	Qualitative semi-structured face to face interviews with 53 users of new psychoactive substances (including 15 chemsex users) and 31 key	Specialist addiction services for problematic users of new psychoactive substances	Not reported	MSM 15	Assumed to be men 15	Not reported

				sexual identity of other participants	stakeholders (e.g. drug service providers) in Manchester, no information provided on the qualitative analysis method					
Ramakrishnan (2023)	Sexual Orientation, Mental Illness, and Substance Use Disorders Among Criminal Legal System-Involved Individuals	USA	195,239	14,995	Quantitative study using data from the 2015-2019 National Survey of Drug Use and Health, comparing sexual minority and heterosexual adults with and without criminal legal system involvement, using multivariable logistic regression, and controlling for sociodemographic characteristics	Substance use disorder treatment services	Not reported	Criminal legal involvement: bisexual 68.5%, gay or lesbian 31.5%<br>No criminal legal involvement: bisexual 61.5%, gay or lesbian 38.5%	Criminal legal involvement: male 39.0%, female 61.0%<br>No criminal legal involvement: male 39.2%, female 60.8%	Criminal legal involvement: Non-Hispanic White (NHW) 51.1%, Non-Hispanic Black (NHB) 22.6%, Non-Hispanic Other (NHO) 6.4%, Hispanic (H) 19.9%<br>No criminal legal involvement: NHW 64.9%, NHB 13.0%, NHO 4.0%, H 18.1%
Rice (2024)	Risk factors of substance use treatment gaps among a nationally representative sample of Black American adults in relation to sexual minority status and health insurance coverage	USA	36,098	Not specifically reported, but 5.9% of the sample gay/lesbian or bisexual	Quantitative analysis of cross-sectional data from 2015-2019 National Survey on Drug Use and Health, examining self-reported substance use treatment gaps for Black Americans	Speciality treatment facilities for illicit drug/alcohol use, including inpatient hospitals, drug/alcohol rehabilitation facilities (in/outpatient) and mental health centres	Not reported (59.9% of total sample ≥35)	Of total sample: bisexual men 0.8%, bisexual women 2.6%, gay men 1.0%, lesbian women 1.1%	Of total sample: 45.8% men, 54.2% women	All of sample Black
Roth (2021)	Longitudinal Analysis of HIV Risk and Substance Use Patterns for Men Who Have Sex With Men and Women and Men Who Have Sex With Men Only	Canada	771	771	Quantitative analysis of data from prospective cohort study in British Columbia, comparing HIV risk and substance use between men who have sex with men only (MSMO) and men who have sex with men and women (MSMW), using multivariable logistic regression	Treatment for substance abuse	MSMO: median 34 (Q1-Q3 26-47)<br>MSMW: median 31 (Q1-Q3 23-40)	Gay 655, bisexual 73, queer/other 46	Male 759, female to male transgender 14, male to female transgender 1	MSMO: White 537, Asian 74, Indigenous 45, Latino/Other 57<br>MSMW: White 47, Asian 0, Indigenous 3, Latino/Other 8
Rowan (2013)	What Is Valued in Gay and Lesbian Specific Alcohol and Other Drug Treatment	USA	40	39	Qualitative study using individual interviews with 24 'alumni' (former clients) and 16 clinical staff of gay-specific addiction treatment programs, analysed using thematic	Gay-specific alcohol and other drug treatment programs	Alumni: 42 (range 33-68)<br>Clinical staff: 44 (range 26-60)	Alumni: gay male 23, lesbian 1<br>Clinical staff: gay male 10, bisexual male 1, lesbian 3, bisexual female 1	Alumni: male 23, female 1<br>Clinical staff: male 11, female 4	Alumni: Caucasian 17, Black 2, Hispanic 1, East Indian 1, Multiriacial 1<br>Clinical staff: Caucasian 15

					analysis and the recovery capital framework					
Rowan (2022)	Health Disparities Between Older Lesbian, Gay, and Bisexual Adults and Heterosexual Adults in the Public Mental Health System	USA	3118	1659	Quantitative study of electronic health records, comparing a matched sample of lesbian, gay, and bisexual adults ≥50 years with a heterosexual sample, examining disparities in health and service use	Inpatient stays related to substance use (including detoxification services, rehabilitation services, and other substance use-related stays)	Not reported (all study participants ≥50)	Gay and bisexual men 844, lesbian and bisexual women 815	Men 844, women 815	Men: White Non-Hispanic 457, Black Non-Hispanic 176, Hispanic 181 Women: White Non-Hispanic 332, Black Non-Hispanic 269, Hispanic 186
Saulnier (1997)	Alcohol problems and marginalization: Social group work with lesbians	USA	12	12	Mixed-methods evaluation of a social work intervention group for lesbian women with alcohol problems, including quantitative assessment of alcohol use and qualitative analysis of the 12 group sessions and individual interviews with each participant, analysed using feminist theory	Group work intervention for problematic alcohol use	34 (range 22-44)	Lesbian 12	Female 12	White 12
Senreich (2009)	A comparison of perceptions, reported abstinence, and completion rates of gay, lesbian, bisexual, and heterosexual clients in substance abuse treatment	USA	227	120	Mixed-methods study comparing heterosexual and LGB former clients of substance abuse treatment programs, examining treatment experiences and outcomes, and reasons for leaving treatment	Substance abuse treatment programs (outpatient programs, residential programs, inpatient rehabs, and methadone maintenance programs)	41.6	Bisexual women 9, bisexual men 13, lesbian women 30, gay men 68	Men 81, women 39	Not reported specifically for SM, of all participants: White 50%, Black 30%, Hispanic 19%, Other 1%
Smiles (2023)	Needle exchange practitioners accounts of delivering harm reduction advice for chemsex: implications for policy and practice	UK	17	Not reported (study of service providers)	Qualitative realist study using interviews with 17 harm reduction practitioners working at needle exchanges, focusing on their understandings of chemsex and its treatment, and analysed using thematic analysis	One integrated substance misuse service with a needle exchange program, one stand-alone harm-reduction service	Not reported (study of service providers)	Not reported (study of service providers)	Male 9, female 8	Not reported (study of service providers)
Sucaldito (2023)	Exploring Individual and Contextual Factors Associated With Sexual Risk and Substance Use Among Underserved GBQMSM and Transgender and	USA	16	16	Qualitative study using interviews with 16 gay, bisexual, queer, and other men who have sex with men in rural Appalachia about risks for HIV, STI and HCV transmission and access to care, analysed	Drug harm reduction programs, including syringe exchange services	33.3 (range 24-50)	Gay/homosexual 6, queer 5, heterosexual MSM 2, pansexual 1, bisexual 1, queer/bisexual/pansexual 1	Cisgender male 5, nonbinary 4, transgender woman 3, genderfluid nonbinary 1, genderfluid transmasculine	White 9, Latino 2, Black 1, Hispanic and Native American 1, Native American/Eastern Band of Cherokee Indians 1, African American and

	Nonbinary Persons in South Central Appalachia				using grounded theory methods				1, transgender man 1, two-spirit 1	Caucasian 1, Mixed, Puerto Rican and Italian 1
Tan (2018)	Chemsex among gay, bisexual, and other men who have sex with men in Singapore and the challenges ahead: A qualitative study	Singapore	30	30	Qualitative study using interviews with 30 gay, bisexual, and other men involved in chemsex, focusing on sexual risk behaviours and chemsex experiences, analysed using applied thematic analysis methods and a socio-ecological framework	Addiction management services	Not reported, range 18-39	Gay, bisexual, and other men who have sex with men 30	Men 30	Not reported
Tan (2021)	Exploring the role of trauma in underpinning sexualised drug use ('chemsex') among gay, bisexual and other men who have sex with men in Singapore	Singapore	33	33	Qualitative study using interviews with 33 gay, bisexual, and other men who have sex with men with a history of sexualised drug use, focusing on experiences of chemsex, treatment-seeking, trauma, and resilience, analysed using thematic analysis methods	Substance use treatment services	Not reported (most participants age 31 to 40)	Gay, bisexual, and other men who have sex with men 33	Men 33	Chinese 25, Malay 3, Indian 3, Others 2
Tomkins (2018)	How can those engaging in chemsex best be supported? An online survey to gain intelligence in Greater Manchester	UK	52	52	Quantitative study using an online convenience survey of MSM involved in chemsex in Greater Manchester, focusing on their sexual health and perceived barriers and facilitators to accessing chemsex support services	Chemsex support services	Not reported (range 25-64)	Men who have sex with men 52	Men 52	Unknown
Travers (1996)	Barriers to accessibility for lesbian and gay youth needing addictions services	Canada	17	17	Qualitative interviews with lesbian and gay 17 to 24 year-olds with experience of using addiction services, exploring barriers to service accessibility, using feminist sociology and existential-phenomenological psychology approaches and analysed thematically	All specialist addiction services, such as residential treatment, addiction counselling programmes, day treatment programmes and/or detox facilities	Not reported (range 17-24)	Gay men 14, lesbian women 3	Male 14, female 3	Caucasian 12, People of Colour (non-Native Canadians) 2, Native Canadians 2
Underhill (2014)	Access to healthcare, HIV/STI testing, and preferred pre-exposure prophylaxis providers among men who have sex with men and men	USA	94	94	Multi-stage qualitative study using focus groups and interviews with men who have sex with men, some of whom were sex workers, asking about access to healthcare, STI	Drug rehabilitation services	Not reported (range 21-70)	MSM 94, of which 7 identified as 'straight/heterosexual'	Male 94	Race: White 72, African American 18, Native American 2, Asian 1, refused 1

	who engage in street-based sex work in the US				testing and PrEP, analysed thematically					Ethnicity: Hispanic/Latino identity 13
Viera (2022)	Substance Use Treatment Engagement among Men Who Have Sex with Men Who Use Stimulants in the Northeastern United States	USA	21	21	Qualitative study using interviews with 21 stimulant-using men who have sex with men, exploring perceptions of and experiences with substance use treatment, analysed using thematic analysis	Substance use treatment services	39.3	Gay 11, straight MSM 3, bisexual 4, other 3	Men 21	White/Caucasian 12, Black/African American 6, Other 3
Washington (2011)	Exploring the perspectives of substance abusing Black men who have sex with men and women in addiction treatment programs: a need for a human sexuality educational model for addiction professionals	USA	105	105	Qualitative study utilising focus groups of Black male sex workers who use injecting drugs, all of whom identified as men who have sex with both women and men, exploring their perspectives on useful topics for addiction professionals to address, analysed using grounded theory	Substance use/addiction treatment services	31.6	Men who have sex with women and men 105	Men 105	Black 105 (of which African American not of Hispanic origin 94, African American Hispanic origin 11)
Willging (2018)	A Mixed-Method Assessment of a Pilot Peer Advocate Intervention for Rural Gender and Sexual Minorities	USA	47	47 sexual and gender minorities	Mixed-methods assessment of a pilot peer advocate intervention for rural gender and sexual minorities in New Mexico; qualitative semi-structured interviews analysed using grounded theory, and quantitative questionnaires analysed descriptively	Specialist mental health/substance use disorder services	35.1 (range 18-61)	Sexual and gender minorities 47, of which: bisexual 19, lesbian/gay 19, questioning 4, other 3, heterosexual 2	Women/girl 23, man/boy 18, transgender 8<br><br>(Note some participants selected more than one category)	American Indian 21, African American 3, Asian American or Asian 1, Non-Hispanic white 14, Hispanic/Latina/o 14<br><br>(Note some participants selected more than one category)

Supplementary Table 5: MMAT-2018 Responses for Included Studies

Note: Mixed methods studies advise rating questions using quality criteria 5, plus 1 and then either 2, 3, or 4.

**First author and year of publication**	**Screening: S1**	**Screening: S2**	**Type of study, MMAT questions used**	**Quality: Q1**	**Quality: Q2**	**Quality: Q3**	**Quality: Q4**	**Quality: Q5**	**Comments**
Allen (2016)	Yes	Yes	Quantitative non-randomised: 3	Yes	Yes	Yes	Yes	Yes	

Aslan (2024)	Yes	Yes	Quantitative descriptive: 4	Yes	Yes	Yes	No	Yes	Response rate 55.2%; likely non-response bias
Barbara (2002)	Yes	Yes	Qualitative: 1	Yes	Yes	Yes	Yes	Yes	
Batchelder (2021)	Yes	Yes	Quantitative non-randomised: 3	Yes	Yes	Yes	No	Yes	No analysis of potential confounders
Bernier (2024)	Yes	Yes	Mixed-methods: 5	No	Yes	Yes	Yes	No	No explicit rationale for mixed methods approach, however derived qualitative themes subsequently analysed quantitatively<br>Qualitative component clearly described and executed; some problems with quantitative components as below
"			+ Qualitative: 1	Yes	Yes	Yes	Yes	Yes	
"			+ Quantitative non-randomised: 3	Can't tell	Yes	Yes	No	Yes	Small sample of SM people, not representatively selected, no reporting of number of people who were approached but declined to participate (i.e. potential non-response bias)<br>No analysis of potential confounders, however exploratory study with small number of SM participants
Blanchette (2024)	Yes	Yes	Qualitative: 1	Yes	Yes	Yes	Yes	Yes	
Bourne (2015)	Yes	Yes	Qualitative: 1	Yes	Yes	Yes	Yes	Yes	
Braine (2014)	Yes	Yes	Qualitative: 1	Yes	Yes	Yes	Yes	Yes	
Brown (2017)	Yes	Yes	Qualitative: 1	Yes	Yes	Yes	Yes	Yes	
Card (2021)	Yes	Yes	Quantitative non-randomised: 3	No	Yes	Yes	Yes	Yes	46.8% of those consented completed the (convenience) survey, likely non-response bias (though included vs excluded participants broadly similar in demographic characteristics)
Card (2022)	Yes	Yes	Quantitative non-randomised: 3	No	Yes	Yes	No	Yes	36.2% of those consented fully completed the (convenience) survey, likely non-response bias (included vs excluded participants broadly similar in demographic characteristics but differ in sexual orientation and readiness to change)<br>No explicit analysis of potential confounders
Cochran (2007)	Yes	Yes	Quantitative descriptive: 4	Yes	Can't tell	Yes	No	Yes	No information reported about non-responders to survey<br>Response rate 23.4%; high risk of non-response bias
Conner (2023)	Yes	Yes	Quantitative non-randomised: 3	Can't tell	Yes	Yes	No	Yes	Although high completion rate of survey, no information reported about potential size of eligible population for survey; convenience sampling<br>Potential confounders not explicitly addressed in analysis
Copeland (1992)	Yes	Yes	Quantitative non-randomised: 3	Can't tell	Yes	Yes	Yes	Yes	Minimal information reported about recruitment processes and no information on response bias (i.e. how many women were eligible/approached but declined to participate)

Corliss (2006)	Yes	Yes	Quantitative descriptive: 4	Yes	Yes	Yes	Can't tell	Yes	Non-response rate not reported (although a number of recruitment methods used to obtain a more representative sample)
Dyar (2023)	Yes	Yes	Quantitative non-randomised: 3	Yes	Yes	Yes	Yes	Yes	
Flores (2017)	Yes	Yes	Quantitative descriptive: 4	Yes	Yes	Yes	No	Yes	Global convenience sample of MSM recruited via online-based methods - high risk of non-response bias
Forenza (2016)	Yes	Yes	Qualitative: 1	Yes	Yes	Yes	Yes	Yes	
Freestone (2025)	Yes	Yes	Qualitative: 1	Yes	Yes	Yes	Yes	Yes	
Goodyear (2020)	Yes	Yes	Qualitative: 1	Yes	Yes	Yes	Yes	Yes	
Gorman (1997)	Yes	Yes	Qualitative: 1	Yes	Yes	Yes	No	Can't tell	Minimal analysis and contextualisation within existing theory, as preliminary report/exploratory study
Graf (2018)	Yes	Yes	Qualitative: 1	Yes	Yes	Yes	No	No	No quotes provided to justify the themes in final results section, 'support system's response to chemsex' Although this is an 'explorative' paper, does not coherently draw on collected data throughout results and discussion sections
Green (2011)	Yes	Yes	Quantitative non-randomised: 3	No	Yes	Yes	No	Yes	29.9% of those who initiated the survey answered one or more questions – high risk of non-response bias Potential confounders not explicitly addressed in analysis
Hall (1994)	Yes	Yes	Qualitative: 1	Yes	Yes	Yes	Yes	Yes	
Haney (2021)	Yes	Yes	Quantitative non-randomised: 3	Yes	Yes	Yes	Yes	Yes	
Hellman (1989)	Yes	Yes	Quantitative descriptive: 4	Yes	Can't tell	Yes	No	Yes	Unable to judge whether respondents differed from target population of service providers 41% response rate, likely non-response bias
Hodges (2023)	Yes	Yes	Quantitative non-randomised: 3	Yes	Yes	Yes	Yes	Yes	
Hsiang (2018)	Yes	Yes	Qualitative: 1	Yes	Yes	Yes	Yes	Yes	
Hussen (2021)	Yes	Yes	Qualitative: 1	Yes	Yes	Yes	Yes	Yes	
Israelstam (1988)	Yes	Yes	Quantitative descriptive: 4	Yes	Yes	Yes	Yes	Yes	
Jeong (2016)	Yes	Yes	Quantitative non-randomised: 3	Yes	Yes	Yes	Yes	Yes	
Joyce (2018)	Yes	Yes	Qualitative: 1	Yes	Yes	Yes	Yes	Yes	
Kanouse (2005)	Yes	Yes	Quantitative non-randomised: 3	Can't tell	Yes	Yes	No	Yes	Small to medium-size convenience sample of hard-to reach population, likely non-response bias Potential confounders not explicitly addressed in analysis
Krasnova (2021)	Yes	Yes	Quantitative non-randomised: 3	Yes	Yes	Yes	Yes	Yes	
Matthews (2006)	Yes	Yes	Qualitative: 1	Yes	No	Yes	No	Yes	Small sample size of 10 participants Mostly short quotations used to illustrate themes, some themes not substantiated by quotations

Mays (1994)	Yes	Yes	Quantitative descriptive: 4	Yes	Can't tell	Yes	No	Yes	Small to medium size convenience sample, unable to ascertain how representative of target population<br>Unable to ascertain number of eligible women who declined to participate; high risk of non-response bias
McGaughey (2023)	Yes	Yes	Qualitative: 1	Yes	No	Yes	No	Yes	Small sample size of 10 participants<br>Mostly short quotations used to illustrate themes, some themes not substantiated by quotations, however note pilot study
Mericle (2018)	Yes	Yes	Qualitative: 1	Yes	Can't tell	Yes	Yes	Yes	Small sample size of 11 participants, however all elite interviews
Micale (2024)	Yes	Yes	Quantitative descriptive: 4	Yes	Yes	Yes	Yes	Yes	
Nieder (2025)	Yes	Yes	Qualitative: 1	Yes	Yes	Yes	Yes	Yes	
Parry (2008)	Yes	Yes	Qualitative: 1	Yes	Yes	Yes	Yes	Yes	
Paschen-Wolff (2024)	Yes	Yes	Qualitative: 1	Yes	Yes	Yes	Yes	Yes	
Penn (2013)	Yes	Yes	Qualitative: 1	Yes	No	Yes	No	Yes	Small sample size of 10 participants<br>Mostly short quotations used to illustrate themes, some themes not substantiated by quotations
Pennay (2018)	Yes	Yes	Qualitative: 1	Yes	Yes	Yes	Yes	Yes	
Ralphs (2018)	Yes	Yes	Qualitative: 1	Yes	Yes	Can't tell	Yes	Can't tell	Qualitative data analysis method not explicitly described or stated (appears to be thematic analysis)
Ramakrishnan (2023)	Yes	Yes	Quantitative non-randomised: 3	Yes	Yes	Yes	Yes	Yes	
Rice (2024)	Yes	Yes	Quantitative non-randomised: 3	Yes	Yes	Yes	Yes	Yes	
Roth (2021)	Yes	Yes	Quantitative non-randomised: 3	Yes	Yes	Yes	Yes	Yes	
Rowan (2013)	Yes	Yes	Qualitative: 1	Yes	Yes	Yes	Yes	Yes	
Rowan (2022)	Yes	Yes	Quantitative non-randomised: 3	Yes	Yes	Yes	Yes	Yes	
Saulnier (1997)	Yes	Yes	Mixed-methods: 5	Can't tell	Yes	Yes	Yes	No	Some rationale for including qualitative and quantitative components, however not fully elaborated<br>Quality issues with component studies
"			+ Qualitative: 1	Yes	Can't tell	Can't tell	Yes	Yes	Small to medium sample size of 12 participants<br>Qualitative analysis method described but not cited, and rationale for choosing that method not provided
"			+ Quantitative descriptive: 4	No	No	Yes	No	Yes	Small to medium size convenience sample unlikely to be representative of target population<br>Inclusion and exclusion criteria not clear<br>High risk of non-response bias
Senreich (2009)	Yes	Yes	Mixed-methods: 5	Can't tell	Yes	Yes	Yes	No	Although mixed-methods design appropriate for research question, no explicit rationale provided in methods<br>Quality issues with component studies

“			+ Qualitative: 1	Yes	No	No	Yes	No	Small qualitative component to larger study, not seeking in-depth qualitative data<br>Data analysis method not described or cited<br>Limited coherence due to above, limited interpretation and integration of qualitative findings
“			+ Quantitative non-randomised: 3	Yes	Yes	Yes	Yes	Yes	
Smiles (2023)	Yes	Yes	Qualitative: 1	Yes	Yes	Yes	Yes	Yes	
Sucaldito (2023)	Yes	Yes	Qualitative: 1	Yes	Yes	Yes	Yes	Yes	
Tan (2018)	Yes	Yes	Qualitative: 1	Yes	Yes	Yes	Yes	Yes	
Tan (2021)	Yes	Yes	Qualitative: 1	Yes	Yes	Yes	Yes	Yes	
Tomkins (2018)	Yes	Yes	Quantitative descriptive: 4	Yes	Can’t tell	Yes	No	Yes	Relatively small convenience sample, unclear how representative of the target population<br>High risk of non-response bias
Travers (1996)	Yes	Yes	Qualitative: 1	Yes	Yes	Can’t tell	Yes	Yes	Analysis method described but not explicitly cited
Underhill (2014)	Yes	Yes	Qualitative: 1	Yes	Yes	Yes	Yes	Yes	
Viera (2022)	Yes	Yes	Qualitative: 1	Yes	Yes	Yes	Yes	Yes	
Washington (2011)	Yes	Yes	Qualitative: 1	Yes	Yes	Yes	Yes	Yes	
Willging (2018)	Yes	Yes	Mixed methods: 5	Yes	Yes	Yes	Yes	No	Some issues with quantitative component study
“			+ Qualitative: 1	Yes	Yes	Yes	Yes	Yes	
“			+ Quantitative descriptive: 4	Yes	Can’t tell	Yes	No	Yes	Medium sample size but hard to ascertain representativeness, potential number of eligible participants – convenience sampling<br>High risk of non-response bias

Supplementary Table 6: Excluded Studies

**Study Title**	**Full Citation**	**Exclusion Reason**	**Additional Notes**
A community-led, harm-reduction approach to chemsex: case study from Australia's largest gay city	Stardust Z, Kolstee J, Joksic S, Gray J, Hannan S. A community-led, harm-reduction approach to chemsex: case study from Australia's largest gay city. Sex Health. 2018;15(2):179-181.	Not a primary research article	Case study
A model for the treatment of lesbian and gay alcohol abusers	Ratner E. A Model for the Treatment of Lesbian and Gay Alcohol Abusers. Alcoholism Treatment Quarterly. 1988; 5(1–2), 25–46.	Not a primary research article	Editorial/practice paper
Alcohol interventions for LGBTQ+ adults: A systematic review	Dimova ED, Elliott L, Frankis J, Drabble L, Wiencierz S, Emslie C. Alcohol interventions for LGBTQ+ adults: A systematic review. Drug Alcohol Rev. 2022;41(1):43-53.	Not a primary research article	Review
Alcohol Use and Alcohol-Related Problems Among Sexual Minority Women	Hughes T. Alcohol Use and Alcohol-Related Problems among Sexual Minority Women. Alcohol Treat Q. 2011;29(4):403-435.	Not a primary research article	Review

Anova Health Institute's harm reduction initiatives for people who use drugs	Hugo JM, Rebe KB, Tsouroulis E, Manion A, de Swart G, Struthers H, McIntyre JA. Anova Health Institute's harm reduction initiatives for people who use drugs. Sex Health. 2018;15(2):176-178.	Not a primary research article	Case report
Chemical Dependency and Depression in Lesbians and Gay Men: What Helps?	Finnegan DG & McNally EB. Chemical Dependency and Depression in Lesbians and Gay Men: What Helps? Journal of Gay & Lesbian Social Services. 1996;4(2):115–129.	Not a primary research article	Review
Comprehensive clinical care for men who have sex with men: an integrated approach	Mayer KH, Bekker LG, Stall R, Grulich AE, Colfax G, Lama JR. Comprehensive clinical care for men who have sex with men: an integrated approach. Lancet. 2012;380(9839):378-87.	Not a primary research article	Review
Correction to: The relationship between sexual and gender stigma and difficulty accessing primary and mental healthcare services among LGBTQI+ populations in Thailand: Findings from a national survey	Moallef S, Salway T, Phanuphak N, Kivioja K, Pongruengphant S and Hayashi K. Correction to: The Relationship Between Sexual and Gender Stigma and Difficulty Accessing Primary and Mental Healthcare Services Among LGBTQI + Populations in Thailand: Findings from a National Survey. Int J Ment Health Addiction. 2024;22: 799.	Not a primary research article	Correction
Counseling the homosexual alcoholic	Colcher RW. Counseling the homosexual alcoholic. J Homosex. 1982;7(4):43-52.	Not a primary research article	Editorial/practice paper
Culturally (in)competent? Dismantling health care barriers for sexual minority women	Dearing RL, Hequembourg AL. Culturally (in)competent? Dismantling health care barriers for sexual minority women. Soc Work Health Care. 2014;53(8):739-61.	Not a primary research article	Review
Developing and testing a brief alcohol intervention for lesbian, gay, bisexual, transgender, and queer populations	Mirabito LA. Developing and Testing a Brief Alcohol Intervention for Lesbian, Gay, Bisexual, Transgender, and Queer Populations. Dissertations. 2021;1058.	Not a primary research article	Dissertation/thesis
Developing and testing of an interactive internet-based intervention to reduce sexual harm of sexualised drug use ('chemsex') among men who have sex with men in Hong Kong: a study protocol for a randomised controlled trial	Choi EPH, Chau PH, Wong WCW, Kowk JYY, Choi KWY, Chow EPF. Developing and testing of an interactive internet-based intervention to reduce sexual harm of sexualised drug use (‘chemsex’) among men who have sex with men in Hong Kong: a study protocol for a randomised controlled trial. BMC Public Health. 2021;21:713.	Not a primary research article	Protocol only
Drug use, dependence and mental health among gay, lesbian and bisexual people reporting regular methamphetamine use: Sydney Australia	Matheson A, Roxburgh A, Degenhardt L, Howard J, Down I. Drug use, dependence and mental health among gay, lesbian and bisexual people reporting regular methamphetamine use: Sydney Australia. 2019.	Not a primary research article	Report
Dual diagnosis issues with homosexual persons	Hellman RE. Dual Diagnosis Issues with Homosexual Persons. Journal of Chemical Dependency Treatment. 1993;5(1):105–117.	Not a primary research article	Case series
Effective intervention and treatment for lesbians	Drabble L & Underhill BL. Effective intervention and treatment for lesbians. In S.L.A. Straussner & S. Brown (Eds.), The Handbook of Addiction Treatment for Women (pp. 399–422). 2002. Jossey-Bass/Wiley.	Not a primary research article	Book chapter
Effectiveness of a culturally tailored SMS alcohol intervention for same-sex attracted women: protocol for an RCT	Bush R, Brown R, McNair R, Orellana L, Lubman DI, Staiger PK. Effectiveness of a culturally tailored SMS alcohol intervention for same-sex attracted women: protocol for an RCT. BMC Womens Health. 2019;19(1):29.	Not a primary research article	Protocol only
Elevated substance use among lesbian and bisexual women: Possible explanations and intervention implications for an urgent public health concern	Rosario M. Elevated substance use among lesbian and bisexual women: possible explanations and intervention implications for an urgent public health concern. Subst Use Misuse. 2008;43(8-9):1268-70.	Not a primary research article	Review/opinion article
Gay and alcoholic: Epidemiologic and clinical issues	Paul JP, Stall R, Bloomfield KA. Gay and alcoholic: Epidemiologic and clinical issues. Alcohol Health & Research World. 1991;15(2):151–160.	Not a primary research article	Review

Impact of perceived public stigma on mental health treatment utilization: differences amongst various demographics	Patel T. Impact of Perceived Public Stigma on Mental Health Treatment Utilization: Differences Amongst Various Demographics. The Chicago School of Professional Psychology ProQuest Dissertations & Theses, 2023. 29993575.	Not a primary research article	Dissertation/thesis
Implementing group therapy for adults with substance use disorders: What research-based evidence?	Lo Coco G, Graffeo MT & Albano G. Implementing group therapy for adults with substance use disorders: What research-based evidence? Group Dynamics: Theory, Research, and Practice. 2024;28(3):199–215.	Not a primary research article	Review
Increasing Cultural Awareness and Sensitivity: Effective Substance Treatment in the Adult Lesbian Population	Taliaferro JD, Lutz B, Moore AK & Scipien K. Increasing Cultural Awareness and Sensitivity: Effective Substance Treatment in the Adult Lesbian Population. Journal of Human Behavior in the Social Environment. 2014;24(5):582–588.	Not a primary research article	Review article
Integrating SMART Recovery and Mental Health Services to Meet the Needs and Goals of LGBTQ Individuals Experiencing Substance Use–Related Problems	McGeough BL, Greenwood EM, Cohen NL & Wootton AR. Integrating SMART Recovery and Mental Health Services to Meet the Needs and Goals of LGBTQ Individuals Experiencing Substance Use–Related Problems. Families in Society. 2022;104(2): 222-233.	Not a primary research article	Editorial
Interventions, Barriers, and Facilitators to Address the Sexual Problems of Gay, Bisexual and Other Men Who Have Sex with Men Living with HIV: A Rapid Scoping Review	Avallone F, Engler K, Cox J, Hickson F, Lebouché B. Interventions, Barriers, and Facilitators to Address the Sexual Problems of Gay, Bisexual and Other Men Who Have Sex with Men Living with HIV: A Rapid Scoping Review. AIDS Behav. 2024;28(2):450-472.	Not a primary research article	Scoping review
Issues and standards in counseling lesbians and gay men with substance abuse concerns	Cheng Z. Issues and Standards in Counseling Lesbians and Gay Men with Substance Abuse Concerns. Journal of Mental Health Counseling. 2003;25(4):323–336.	Not a primary research article	Review
Lesbian alcohol and substance abuse	Mosbacher D. Lesbian Alcohol and Substance Abuse. Psychiatric Annals. 1988;18(1):47-50.	Not a primary research article	Commentary
Meeting the Needs of the Gay and Lesbian Community: Outcomes in the Human Services	Maccio EM & Doueck HJ. Meeting the Needs of the Gay and Lesbian Community: Outcomes in the Human Services. Journal of Gay & Lesbian Social Services. 2002;14(4): 55–73.	Not a primary research article	Review
Men who have sex with men: Health risks, methamphetamine use, and approaches to preventive care	Espejo JB. Men who have sex with men: Health risks, methamphetamine use, and approaches to preventive care. Journal of Men's Health. 2013;10(1):8–13.	Not a primary research article	Case study
Mental health disparities in sexual minority and transgender women: Implications and considerations for treatment	Matouk KM, Schulman JK, Case JAC. Mental Health Disparities in Sexual Minority and Transgender Women: Implications and Considerations for Treatment. Psychiatr Clin North Am. 2023;46(3):583-595.	Not a primary research article	Editorial/review
Methamphetamine abuse: Issues for special populations	Freese TE, Obert J, Dickow A, Cohen J, Lord RH. Methamphetamine abuse: issues for special populations. J Psychoactive Drugs. 2000;32(2):177-82.	Not a primary research article	Case series/review
Mobile-Enhanced Prevention Support Study for Men Who Have Sex With Men and Transgender Women Leaving Jail: Protocol for a Randomized Controlled Trial	Edwards GG, Reback CJ, Cunningham WE, Hilliard CL, McWells C, Mukherjee S, Weiss RE, Harawa NT. Mobile-Enhanced Prevention Support Study for Men Who Have Sex With Men and Transgender Women Leaving Jail: Protocol for a Randomized Controlled Trial. JMIR Res Protoc. 2020;9(9):e18106.	Not a primary research article	Protocol only
Online intervention as strategy to reach men who have sex with other men and who use substances in a sexual context. Development of the MONBUZZ.ca project	Flores-Aranda J, Goyette M, Larose-Osterrath C. Online Intervention as Strategy to Reach Men Who Have Sex With Other Men and Who Use Substances in a Sexual Context. Development of the MONBUZZ.ca Project. Front Psychiatry. 2019;10:183.	Not a primary research article	Literature review and service evaluation

Optimizing Contingency Management With Methamphetamine-Using Men Who Have Sex With Men	Gómez W, Olem D, Andrews R, Discepola MV, Ambrose P, Dilworth SE, Carrico AW. Optimizing Contingency Management with Methamphetamine-Using Men who Have Sex with Men. Cogn Behav Pract. 2018;25(2):286-295.	Not a primary research article	Program evaluation
Providing care for lesbian, gay, bisexual, and transgender immigrants at health centers and clinics	Keuroghlian AS, McDowell MJ, Stern TA. Providing Care for Lesbian, Gay, Bisexual, and Transgender Immigrants at Health Centers and Clinics. Psychosomatics. 2018;59(2):193-198.	Not a primary research article	Case report
Providing Unique Support for Health Study Among Young Black and Latinx Men Who Have Sex With Men and Young Black and Latinx Transgender Women Living in 3 Urban Cities in the United States: Protocol for a Coach-Based Mobile-Enhanced Randomized Control Trial	Arrington-Sanders R, Hailey-Fair K, Wirtz A, Cos T, Galai N, Brooks D, Castillo M, Dowshen N, Trexler C, D'Angelo LJ, Kwait J, Beyrer C, Morgan A, Celentano D; PUSH Study. Providing Unique Support for Health Study Among Young Black and Latinx Men Who Have Sex With Men and Young Black and Latinx Transgender Women Living in 3 Urban Cities in the United States: Protocol for a Coach-Based Mobile-Enhanced Randomized Control Trial. JMIR Res Protoc. 2020;9(9):e17269.	Not a primary research article	Protocol only
Queering recovery: A proposed model for LGBTQ+-affirmative relapse prevention	Chaney MP, Mason N. Queering recovery: A proposed model for LGBTQ+-affirmative relapse prevention. Journal of Addictions and Offender Counseling. 2024;45(1):98-110.	Not a primary research article	Editorial/comment
Service Engagement with High-Risk Men Who Have Sex with Men: Challenges and Implications for Social Work Practice	Natale AP, Moxley DP. Service engagement with high-risk men who have sex with men: challenges and implications for social work practice. Soc Work Health Care. 2009;48(1):38-56.	Not a primary research article	Review
Sex like you can't even imagine: "Crystal," crack and gay men	Guss JR. Sex Like You Can’t Even Imagine: “Crystal,” Crack and Gay Men. Journal of Gay & Lesbian Psychotherapy. 2000;3(3–4):105–122.	Not a primary research article	Comment/clinical practice article
Sexualised drug use among LGBT people: A mixed methods study of reasons for engagement and associations with physical and psychological wellbeing	Hibbert M. Sexualised drug use among LGBT people: a mixed methods study of reasons for engagement and associations with physical and psychological wellbeing. Doctoral thesis, Liverpool John Moores University. 2020.	Not a primary research article	Thesis/dissertation
Specific approaches and techniques in the treatment of gay male alcohol abusers	Smith TM. Specific Approaches and Techniques in the Treatment of Gay Male Alcohol Abusers. Journal of Homosexuality. 1982;7(4):53–69.	Not a primary research article	Review
Substance Abuse and Dependency in Gay Men and Lesbians	Anderson SC. Substance Abuse and Dependency in Gay Men and Lesbians. Journal of Gay & Lesbian Social Services. 1996;5(1):59–76.	Not a primary research article	Review
Substance Abuse Treatment as HIV Prevention for Men Who Have Sex with Men	Shoptaw S, Frosch D. Substance Abuse Treatment as HIV Prevention for Men Who Have Sex with Men. AIDS Behav. 2000;4:193–203.	Not a primary research article	Review
Substance abuse, internalized homophobia, and gay men and lesbians: Psychodynamic issues and clinical implications	Cabaj RP. Substance abuse, internalized homophobia, and gay men and lesbians: Psychodynamic issues and clinical implications. In J.R. Guss & J. Drescher (Eds.), Addictions in the Gay and Lesbian Community. (pp. 5–24). 2000. Haworth Press.	Not a primary research article	Book chapter
Substance Use in Lesbian, Gay, and Bisexual Populations: An Update on Empirical Research and Implications for Treatment	Green KE, Feinstein BA. Substance use in lesbian, gay, and bisexual populations: an update on empirical research and implications for treatment. Psychol Addict Behav. 2012;26(2):265-78.	Not a primary research article	Review
The check-up: In-person, computerized, and telephone adaptations of motivational enhancement treatment to elicit voluntary participation by the contemplator	Walker DD, Roffman RA, Picciano JF, Stephens RS. The check-up: in-person, computerized, and telephone adaptations of motivational enhancement treatment to elicit voluntary participation by the contemplator. Subst Abuse Treat Prev Policy. 2007;2:2.	Not a primary research article	Review/clinical practice paper

The experiences of LGBQ clients accessing substance use treatment: Understanding barriers and facilitators through minority stress theory	Spalding A. The experiences of LGBQ clients accessing substance use treatment: Understanding barriers and facilitators through minority stress theory. Alliant International University ProQuest Dissertations & Theses, 2023.	Not a primary research article	Thesis/dissertation
The impacts of internalized homonegativity, shame and self-compassion on quality of recovery in gay, bisexual and queer males in addiction recovery	Campagna DL. The impacts of internalized homonegativity, shame and self-compassion on quality of recovery in gay, bisexual and queer males in addiction recovery. Alliant International University ProQuest Dissertations & Theses, 2022.	Not a primary research article	Thesis/dissertation
Treating gay, lesbian, bisexual and transgender professionals with addictive disease	Ziegler PP. Treating Gay, Lesbian, Bisexual and Transgender Professionals with Addictive Disease. Journal of Gay & Lesbian Psychotherapy. 2000;3(3–4):59–68.	Not a primary research article	Review/clinical practice paper
Understanding the experiences of gay men in addiction treatment: a phenomenological study	Cullen J. Understanding the experiences of gay men in addiction treatment: a phenomenological study. Thesis (Ph.D.)-University of Toronto, 2004.	Not a primary research article	Thesis/dissertation
Understanding the Impact of Childhood Sexual Abuse on Men's Risk Behavior: Protocol for a Mixed-Methods Study	Downing MJ Jr, Brown D, Steen J, Benoit E. Understanding the Impact of Childhood Sexual Abuse on Men's Risk Behavior: Protocol for a Mixed-Methods Study. JMIR Res Protoc. 2018;7(2):e62.	Not a primary research article	Protocol only
What really worked? A case analysis and discussion of confrontational intervention for substance abuse in marginalized women	Hall JM. What really worked? A case analysis and discussion of confrontational intervention for substance abuse in marginalized women. Arch Psychiatr Nurs. 1993;7(6):322-7.	Not a primary research article	Case study
Who should be doing what about the gay alcoholic?	Zigrang TA. Who Should Be Doing What about the Gay Alcoholic? Journal of Homosexuality. 1982;7(4):27–35.	Not a primary research article	Review/commentary
Staying Negative -- It's Not Automatic: A Harm-Reduction Approach to Substance Use and Sex	Elovich R. Staying negative--it's not automatic: a harm-reduction approach to substance use and sex. AIDS Public Policy J. 1996;11(2):66-77.	Not in adults	
Failure to get into substance abuse treatment	Fisher DG, Reynolds GL, D'Anna LH, Hosmer DW, Hardan-Khalil K. Failure to get into substance abuse treatment. J Subst Abuse Treat. 2017;73:55-62.	Not about sexual minority people	Data aggregated between heterosexual and SM people for the barriers to access, so unable to draw conclusions
Structural determinants of tailored behavioral health services for sexual and gender minorities in the United States, 2010 to 2020: a panel analysis	Cascalheira CJ, Helminen EC, Shaw TJ, Scheer JR. Structural determinants of tailored behavioral health services for sexual and gender minorities in the United States, 2010 to 2020: a panel analysis. BMC Public Health. 2022;22(1):1908.	Not about sexual minority people	
"What if I get sick, where shall I go?": a qualitative investigation of healthcare engagement among young gay and bisexual men in Nairobi, Kenya	Mwaniki SW, Kaberia PM, Mugo PM, Palanee-Phillips T. "What if I get sick, where shall I go?": a qualitative investigation of healthcare engagement among young gay and bisexual men in Nairobi, Kenya. BMC Public Health. 2024;24(1):52.	Not about specialist addiction services	
'It kills the freedom or the spirit of people being who they are': impact of sexuality-based stigma and discrimination on the lives of gay and bisexual men in Kenya	Jauregui JC, Lewis KA, Moore DM, Ogunbajo A, Odero WW, Wambaya J, Onyango DP, Jadwin-Cakmak L, Harper GW. 'It kills the freedom or the spirit of people being who they are': impact of sexuality-based stigma and discrimination on the lives of gay and bisexual men in Kenya. Global Public Health. 2025;20(1).	Not about specialist addiction services	
A qualitative study to explore the healthcare-seeking experiences of men who have sex with men (MSM) and transgender women (TGW) in Rwanda	Isano S, Yohannes T, Igihozo G, Ndatinya GI & Wong R. A qualitative study to explore the healthcare-seeking experiences of men who have sex with men (MSM) and transgender women (TGW) in Rwanda. BMC Health Serv Res. 2023;23:291.	Not about specialist addiction services	
Alcohol and tobacco consumption among Australian sexual minority women: Patterns of use and service engagement	Amos N, Bourne A, Hill AO, Power J, McNair R, Mooney-Somers J, Pennay A, Carman M, Lyons A. Alcohol and tobacco consumption among Australian sexual minority women: Patterns of use and service engagement. Int J Drug Policy. 2022;100:103516.	Not about specialist addiction services	Not entirely clear that discussing specialist addictions services - 'professional support for alcohol use'

Alcohol use, behavioral and mental health help-seeking, and treatment satisfaction among sexual minority women	Scheer JR, Batchelder AW, Bochicchio LA, Kidd JD, Hughes TL. Alcohol use, behavioral and mental health help-seeking, and treatment satisfaction among sexual minority women. Alcohol Clin Exp Res. 2022;46(4):641-656.	Not about specialist addiction services	
Assessing the feasibility of harm reduction services for MSM: The late night breakfast buffet study	Rose VJ, Raymond HF, Kellogg TA, McFarland W. Assessing the feasibility of harm reduction services for MSM: the late night breakfast buffet study. Harm Reduct J. 2006;3:29.	Not about specialist addiction services	
At the intersection of marginalised identities: lesbian, gay, bisexual and transgender people's experiences of injecting drug use and hepatitis C seroconversion	Deacon RM, Mooney-Somers J, Treloar C, Maher L. At the intersection of marginalised identities: lesbian, gay, bisexual and transgender people's experiences of injecting drug use and hepatitis C seroconversion. Health Soc Care Community. 2013;21(4):402-10.	Not about specialist addiction services	
Barriers and facilitators to medication-assisted treatment for cocaine use disorder among men who have sex with men: a qualitative study	Hsiang E, Patel K, Wilson EC, Dunham A, Ikeda J, Matheson T, Santos GM. Barriers and facilitators to medication-assisted treatment for cocaine use disorder among men who have sex with men: a qualitative study. Addict Sci Clin Pract. 2024;19(1):84.	Not about specialist addiction services	
Barriers and unmet need for supportive services for HIV patients in care in Los Angeles County, California.	Wohl AR, Carlos JA, Tejero J, Dierst-Davies R, Daar ES, Khanlou H, Cadden J, Towner W, Frye D. Barriers and unmet need for supportive services for HIV patients in care in Los Angeles County, California. AIDS Patient Care STDS. 2011;25(9):525-32.	Not about specialist addiction services	
Changes in alcohol, tobacco, cannabis, and other substance use and its association with mental health during the COVID-19 pandemic among sexual minority men in Eastern European and Central Asian countries	Hong C, Mammadli T, Lunchenkov N, Garner A, Howell S, Holloway IW. Changes in alcohol, tobacco, cannabis, and other substance use and its association with mental health during the COVID-19 pandemic among sexual minority men in Eastern European and Central Asian countries. J Affect Disord. 2024;359:302-307.	Not about specialist addiction services	
Chems4EU: chemsex use and its impacts across four European countries in HIV-positive men who have sex with men attending HIV services	Whitlock GG, Protopapas K, Bernardino JI, Imaz A, Curran A, Stingone C, Shivasankar S, Edwards S, Herbert S, Thomas K, Mican R, Prieto P, Nestor Garcia J, Andreoni M, Hill S, Okhai H, Stuart D, Bourne A, Conway K. Chems4EU: chemsex use and its impacts across four European countries in HIV-positive men who have sex with men attending HIV services. HIV Med. 2021;22(10):944-957.	Not about specialist addiction services	More relevant to sexual health clinic support (e.g. 56 Dean St, 'professional chemsex support')
Chemsex and Psychosexual Health in a Large Italian Sample of Men Who Have Sex with Men (MSM)	Pessina R, Pavanello Decaro S, Torri C, Prunas A. Chemsex and Psychosexual Health in a Large Italian Sample of Men Who Have Sex with Men (MSM). Sexuality & Culture. 2025;10.1007/s12119-025-10339-y.	Not about specialist addiction services	Reports on generic 'professional support for chemsex' but no participants had attended specific addiction services
Comparing substance use and mental health among sexual and gender minority and heterosexual cisgender youth experiencing homelessness	Hao J, Beld M, Khoddam-Khorasani L, Flentje A, Kersey E, Mousseau H, Frank J, Leonard A, Kevany S, Dawson-Rose C. Comparing substance use and mental health among sexual and gender minority and heterosexual cisgender youth experiencing homelessness. PLoS One. 2021;16(3):e0248077.	Not about specialist addiction services	
Correlates of shared methamphetamine injection among methamphetamine-injecting treatment seekers: the first report from Iran	Mehrjerdi ZA, Abarashi Z, Noroozi A, Arshad L, Zarghami M. Correlates of shared methamphetamine injection among methamphetamine-injecting treatment seekers: the first report from Iran. Int J STD AIDS. 2014;25(6):420-7.	Not about specialist addiction services	
Demand for and availability of specialist chemsex services in the UK: A cross-sectional survey of sexual health clinics	Wiggins H, Ogaz D, Mebrahtu H, Sullivan A, Bowden-Jones O, Field N, Hughes G. Demand for and availability of specialist chemsex services in the UK: A cross-sectional survey of sexual health clinics. Int J Drug Policy. 2018;55:155-158.	Not about specialist addiction services	
Describing the effect of COVID-19 on sexual and healthcare-seeking behaviours of men who have sex with men in three counties in Kenya: a cross-sectional study	Shaw SY, Biegun JCS, Leung S, Isac S, Musyoki HK, Mugambi M, Kioko J, Musimbi J, Olango K, Kuria S, Ongaro MK, Walimbwa J, Emmanuel F, Blanchard J, Pickles M, Mishra S, Becker ML, Lazarus L, Lorway R, Bhattacharjee P. Describing the effect of COVID-19	Not about specialist addiction services	

	on sexual and healthcare-seeking behaviours of men who have sex with men in three counties in Kenya: a cross-sectional study. Sex Transm Infect. 2024;100(8):497-503.		
Development of a Small-Group Intervention for Stimulant-Using Men Who Have Sex With Men	Lyons T, Tilmon S & Fontaine YM. Development of a Small-Group Intervention for Stimulant-Using Men Who Have Sex With Men. Journal of Groups in Addiction & Recovery. 2014;9(1):54–70.	Not about specialist addiction services	
Differences in healthcare access, use, and experiences within a community sample of racially diverse lesbian, gay, bisexual, transgender, and questioning emerging adults	Macapagal K, Bhatia R, Greene GJ. Differences in Healthcare Access, Use, and Experiences Within a Community Sample of Racially Diverse Lesbian, Gay, Bisexual, Transgender, and Questioning Emerging Adults. LGBT Health. 2016;3(6):434-442.	Not about specialist addiction services	
Emergency Healthcare Utilization and Unmet Care Needs in Chemsex Users: A Cross-Sectional Survey among Sexual Minority Men	Gonzalez-Recio P, Moreno-García S, Donat M, Palma D, Guerras JM, Belza MJ. Emergency Healthcare Utilization and Unmet Care Needs in Chemsex Users: A Cross-Sectional Survey among Sexual Minority Men. J Community Health. 2025;10.1007/s10900-024-01440-8.	Not about specialist addiction services	
Enablers and inhibitors to the utilization of healthcare services by members of the LGBTQ plus community in Accra, Ghana	Anuga D, Iddrisu M, Konlan K. Enablers and inhibitors to the utilization of healthcare services by members of the LGBTQ+ community in Accra, Ghana. Discover Public Health. 2025;22(1):10.1186/s12982-025-00556-z.	Not about specialist addiction services	
Estimates of alcohol use and clinical treatment needs among homosexually active men and women in the U.S. population	Cochran SD, Keenan C, Schober C, Mays VM. Estimates of alcohol use and clinical treatment needs among homosexually active men and women in the U.S. population. J Consult Clin Psychol. 2000;68(6):1062-71.	Not about specialist addiction services	
Experiences of stigma and subsequent reduced access to health care among women who inject drugs	Brener L, Cama E, Broady T, Harrod ME, Holly C, Caruana T, Beadman K, Treloar C. Experiences of stigma and subsequent reduced access to health care among women who inject drugs. Drug Alcohol Rev. 2024;43(5):1071-1079.	Not about specialist addiction services	
Exploring Anabolic Androgenic Steroid Use Among Cisgender Gay, Bisexual, and Queer Men	Kutscher E, Arshed A, Greene RE, Kladney M. Exploring Anabolic Androgenic Steroid Use Among Cisgender Gay, Bisexual, and Queer Men. JAMA Netw Open. 2024;7(5):e2411088.	Not about specialist addiction services	
Factors related to counselor preference among gays and lesbians	McDermott D, Tyndall L, Lichtenberg JW. Factors related to counselor preference among gays and lesbians. Journal of Counseling & Development. 1989;68(1):31–35.	Not about specialist addiction services	
Feasibility, acceptability, and perceived usefulness of a community-evidence-based harm reduction intervention for sexualized stimulant use among Mexican gay, bisexual, and other men who have sex with men	Rafful C, Orozco R, Peralta D, Jiménez-Rivagorza L, Medina-Mora ME, Gutiérrez N, Morales-Gutierrez M. Feasibility, acceptability, and perceived usefulness of a community-evidence-based harm reduction intervention for sexualized stimulant use among Mexican gay, bisexual, and other men who have sex with men. Harm Reduct J. 2024;21(1):95.	Not about specialist addiction services	
Gay, bisexual, and other men who have sex with men: Barriers and facilitators to healthcare access in Ottawa	Haines M, O'Byrne P, & MacPherson P. Gay, bisexual, and other men who have sex with men: Barriers and facilitators to healthcare access in Ottawa. The Canadian Journal of Human Sexuality. 2021;30(3):339-348.	Not about specialist addiction services	
Health care access and health behaviors among men who have sex with men: The cost of health disparities	McKirnan DJ, Du Bois SN, Alvy LM, Jones K. Health care access and health behaviors among men who have sex with men: the cost of health disparities. Health Educ Behav. 2013;40(1):32-41.	Not about specialist addiction services	
Health disparities in access to health care for HIV infection, substance abuse, and mental health among	Loza O, Provencio-Vasquez E, Mancera B, & De Santis J. Health disparities in access to health care for HIV infection, substance abuse, and mental health among Latino men	Not about specialist addiction services	

Latino men who have sex with men in a U.S.–Mexico Border City	who have sex with men in a U.S.–Mexico Border City. Journal of Gay & Lesbian Social Services. 2021;33(3):320–336.		
Health status, health behaviours and healthcare access of lesbian, gay, bisexual and transgender populations in Turkey	Ercan Sahin N, Aslan F, Emiroglu ON. Health status, health behaviours and healthcare access of lesbian, gay, bisexual and transgender populations in Turkey. Scand J Caring Sci. 2020;34(1):239-246.	Not about specialist addiction services	
Impacts of COVID-19 on sexual risk behaviors, safe injection practices, and access to HIV services among key populations in Zambia: Findings from a rapid qualitative formative assessment	Parmley LE, Nkumbula T, Chilukutu L, Chelu L, Mulemfwe C, Hanunka B, Mwale J, Neal J, Handema R, Kasonde P, Mutale K, Sakala HM, Lahuerta M. Impacts of COVID-19 on sexual risk behaviors, safe injection practices, and access to HIV services among key populations in Zambia: Findings from a rapid qualitative formative assessment. PLoS One. 2023;18(8):e0289007.	Not about specialist addiction services	
Information-seeking behaviours in Australian sexual minority men engaged in chemsex	Demant D, Carroll JA, Saliba B, Bourne A. Information-seeking behaviours in Australian sexual minority men engaged in chemsex. Addict Behav Rep. 2021;16:100399.	Not about specialist addiction services	
Issues in psychotherapy with lesbians and gay men: A survey of psychologists	Garnets L, Hancock KA, Cochran SD, Goodchilds J, Peplau LA. Issues in psychotherapy with lesbians and gay men. A survey of psychologists. Am Psychol. 1991;46(9):964-72.	Not about specialist addiction services	
Issues in the treatment of lesbian and gay men with chronic mental illness	Hellman RE. Issues in the treatment of lesbian women and gay men with chronic mental illness. Psychiatr Serv. 1996;47(10):1093-8.	Not about specialist addiction services	
Lesbian uses of and satisfaction with mental health services: results from Boston Lesbian Health Project	Sorensen L, Roberts SJ. Lesbian uses of and satisfaction with mental health services: results from Boston Lesbian Health Project. J Homosex. 1997;33(1):35-49.	Not about specialist addiction services	
Lesbian, Gay, Bisexual, Sexual-Orientation Questioning Adolescents Seeking Mental Health Services: Risk Factors, Worries, and Desire to Talk About Them	Cniro D, Surko M, Bhandarkar K, Helfgott N, Peake K & Epstein I. Lesbian, Gay, Bisexual, Sexual-Orientation Questioning Adolescents Seeking Mental Health Services: Risk Factors, Worries, and Desire to Talk About Them. Social Work in Mental Health. 2005;3(3):213–234.	Not about specialist addiction services	
Lessons on social and health disparities from older lesbians with alcoholism and the role of interventions to promote culturally competent services	Rowan NL & Giunta N. Lessons on social and health disparities from older lesbians with alcoholism and the role of interventions to promote culturally competent services. Journal of Human Behavior in the Social Environment. 2015;26(2):210–216.	Not about specialist addiction services	
Links between Childhood Abuse, Insidious Trauma, and Methamphetamine Use across the Lifespan among Gay, Bisexual, and Other Men Who Have Sex with Men: A Qualitative Analysis	Berlin GW, Fulcher K, Taylor K, Nguyen T, Montiel A, Moore D, Hull M, Lachowsky NJ. Links Between Childhood Abuse, Insidious Trauma, and Methamphetamine Use Across the Lifespan Among Gay, Bisexual, and Other Men Who Have Sex with Men: A Qualitative Analysis. J Homosex. 2023;70(13):3192-3212.	Not about specialist addiction services	Paper describes 'support services' rather than specific addiction services
Mental Health Problems and Use of Services of Lesbians: Results of the Boston Lesbian Health Project II	Roberts SJ, Grindel CG, Patsdaughter CA, Reardon K & Tarmina MS. Mental Health Problems and Use of Services of Lesbians: Results of the Boston Lesbian Health Project II. Journal of Gay & Lesbian Social Services. 2004;17(4):1–16.	Not about specialist addiction services	
Mental Health Service Utilization Among Young Black Gay, Bisexual, and Other Men Who Have Sex with Men in HIV Care: A Retrospective Cohort Study	Hussen SA, Camp DM, Wondmeneh SB, Doraivelu K, Holbrook N, Moore SJ, Colasanti JA, Ali MK, Farber EW. Mental Health Service Utilization Among Young Black Gay, Bisexual, and Other Men Who Have Sex with Men in HIV Care: A Retrospective Cohort Study. AIDS Patient Care STDS. 2021;35(1):9-14.	Not about specialist addiction services	Aggregates findings for all mental health referrals, unable to disaggregate specialist addiction services
Missed opportunities for healthcare providers to discuss HIV preexposure prophylaxis with people who inject drugs	Vincent W, McFarland W. Missed opportunities for healthcare providers to discuss HIV preexposure prophylaxis with people who inject drugs. Int J Drug Policy. 2022;110:103873.	Not about specialist addiction services	

Motivations for opioid and stimulant use among drug using black sexual minority men: A life course perspective	Dangerfield DT, Heidari O, Cooper J, Allen S, Lucas GM. Motivations for opioid and stimulant use among drug using black sexual minority men: A life course perspective. Drug Alcohol Depend. 2020;215:108224.	Not about specialist addiction services	
Multivariate Patterns of Lifetime Adverse Experiences and Substance Use: Implications for Counseling Services for Lesbian and Bisexual Women	Tubman JG, Moore C, Lee J, Shapiro AJ. Multivariate Patterns of Lifetime Adverse Experiences and Substance Use: Implications for Counseling Services for Lesbian and Bisexual Women. Journal of LGBTQ Issues in Counseling. 2024;18(4):343–362.	Not about specialist addiction services	
Nursing and Health Care Preferences Among Opioid and Stimulant Using Black Sexual Minority Men: An Exploratory Study	Dangerfield DT, Cooper J, Heidari O, Allen S, Winder TJA, Lucas GM. Nursing and Health Care Preferences Among Opioid and Stimulant Using Black Sexual Minority Men: An Exploratory Study. J Assoc Nurses AIDS Care. 2021;32(5):e30-e39.	Not about specialist addiction services	
Older, gay men's navigation of mental health and substance use challenges: A qualitative exploration	Handlovsky I, Wonsiak T, Zakher B, Ferlatte O, Kia H, Oliffe JL. Older, gay men's navigation of mental health and substance use challenges: A qualitative exploration. SSM - Qualitative Research in Health. 2024;6:100484.	Not about specialist addiction services	
Perceived unmet substance use and mental health care needs of acute care patients who use drugs: A cross-sectional analysis using the Behavioral Model for Vulnerable Populations	Kosteniuk B, Salvalaggio G, Wild TC, Gelberg L, Hyshka E. Perceived unmet substance use and mental health care needs of acute care patients who use drugs: A cross-sectional analysis using the Behavioral Model for Vulnerable Populations. Drug Alcohol Rev. 2022;41(4):830-840.	Not about specialist addiction services	Unable to disaggregate substance use services and mental health services
Perspectives on Pain, Engagement in HIV Care, and Behavioral Interventions for Chronic Pain Among Older Sexual Minority Men Living with HIV and Chronic Pain: A Qualitative Analysis	McKetchnie SM, Beaugard C, Taylor SW, O'Cleirigh C. Perspectives on Pain, Engagement in HIV Care, and Behavioral Interventions for Chronic Pain Among Older Sexual Minority Men Living with HIV and Chronic Pain: A Qualitative Analysis. Pain Med. 2021;22(3):577-584.	Not about specialist addiction services	
Perspectives on unhealthy alcohol use among men who have sex with men prescribed HIV pre-exposure prophylaxis: A qualitative study	Strong SH, Oldfield BJ, van den Berg JJ, Cole CA, Biegacki E, Ogbuagu O, Virata M, Chan PA, Edelman EJ. Perspectives on unhealthy alcohol use among men who have sex with men prescribed HIV pre-exposure prophylaxis: A qualitative study. Prev Med Rep. 2023;37:102553.	Not about specialist addiction services	Treatment and support to reduce alcohol use conceptualised generally, not clear if talking about specialist addictions services or wider support/therapy/counselling for alcohol problems
Pre-Empting Stigma and Complicating Trauma: Narratives of Gay and Bisexual Men who Inject Drugs in Australia	Schroeder SE, Treloar C, Bourne A, Stoové M, Doyle J, Hellard M, Pedrana A. Pre-Empting Stigma and Complicating Trauma: Narratives of Gay and Bisexual Men who Inject Drugs in Australia. Qual Health Res. 2023;33(8-9):688-700.	Not about specialist addiction services	
Problematic recreational drug use: is there a role for outpatient sexual health clinics in identifying those not already engaged with treatment services?	Lovett C, Yamamoto T, Hunter L, White J, Dargan PI, Wood DM. Problematic recreational drug use: is there a role for outpatient sexual health clinics in identifying those not already engaged with treatment services? Sex Health. 2015;12(6):501-5.	Not about specialist addiction services	
ReACH2Gether: Iterative Development of a Couples-Based Intervention to Reduce Alcohol use Among Sexual Minority Men Living with HIV and Their Partners	Gamarel KE, Durst A, Zelaya DG, van den Berg JJ, Souza T, Johnson MO, Wu E, Monti PM, Kahler CW. ReACH2Gether: Iterative Development of a Couples-Based Intervention to Reduce Alcohol use Among Sexual Minority Men Living with HIV and Their Partners. AIDS Behav. 2024;28(4):1244-1256.	Not about specialist addiction services	
Scaling Up and Out HIV Prevention and Behavioral Health Services to Latino Sexual Minority Men in South Florida: Multi-Level Implementation Barriers, Facilitators, and Strategies	Harkness A, Satyanarayana S, Mayo D, Smith-Alvarez R, Rogers BG, Prado G, Safren SA. Scaling Up and Out HIV Prevention and Behavioral Health Services to Latino Sexual Minority Men in South Florida: Multi-Level Implementation Barriers, Facilitators, and Strategies. AIDS Patient Care STDS. 2021;35(5):167-179.	Not about specialist addiction services	No disaggregation of HIV/PrEP/sexual health/substance services

Service provision and barriers to care for men who have sex with men engaging in chemsex and sexualised drug use in England	Hibbert MP, Germain JS, Brett CE, Van Hout MC, Hope VD, Porcellato LA. Service provision and barriers to care for men who have sex with men engaging in chemsex and sexualised drug use in England. Int J Drug Policy. 2021;92:103090.	Not about specialist addiction services	Focuses on sexual health services rather than specialist addiction services
Sex, drugs and techno - a qualitative study on finding the balance between risk, safety and pleasure among men who have sex with men engaging in recreational and sexualised drug use	Dennermalm N, Scarlett J, Thomsen S, Persson KI, Alvesson HM. Sex, drugs and techno - a qualitative study on finding the balance between risk, safety and pleasure among men who have sex with men engaging in recreational and sexualised drug use. BMC Public Health. 2021;21(1):863.	Not about specialist addiction services	
Sexual, addiction and mental health care needs among men who have sex with men practicing chemsex – a cross-sectional study in the Netherlands	Evers YJ, Hoebe CJPA, Dukers-Muijrers NHTM, Kampman CJG, Kuizenga-Wessel S, Shilue D, Bakker NCM, Schamp SMAA, Van Buel H, Van Der Meijden WCJPM, Van Liere GAFS. Sexual, addiction and mental health care needs among men who have sex with men practicing chemsex - a cross-sectional study in the Netherlands. Prev Med Rep. 2020;18:101074.	Not about specialist addiction services	
Sexual behavior, sexual identity, and substance abuse among low-income bisexual and non-gay-identifying African American men who have sex with men	Harawa NT, Williams JK, Ramamurthi HC, Manago C, Avina S, Jones M. Sexual behavior, sexual identity, and substance abuse among low-income bisexual and non-gay-identifying African American men who have sex with men. Arch Sex Behav. 2008;37(5):748-62.	Not about specialist addiction services	
Sexual risk and sexual healthcare utilization profiles among Black sexual minority men in the U.S. Deep South	Heidari O, Dangerfield DT & Hickson DA. Sexual risk and sexual healthcare utilization profiles among Black sexual minority men in the U.S. Deep South. AIDS Care. 2020;32(12):1602–1609.	Not about specialist addiction services	
Sexualized drug use and specialist service experience among MSM attending urban and rural sexual health clinics in England and Scotland	Kennedy R, Murira J, Foster K, Heinsbroek E, Keane F, Pal N, Chalmers L, Sinka K. Sexualized drug use and specialist service experience among MSM attending urban and rural sexual health clinics in England and Scotland. Int J STD AIDS. 2021;32(14):1338-1346.	Not about specialist addiction services	
Stigma and help-seeking: The interplay of substance use and gender and sexual minority identity	Benz MB, Palm Reed K, Bishop LS. Stigma and help-seeking: The interplay of substance use and gender and sexual minority identity. Addict Behav. 2019;97:63-69.	Not about specialist addiction services	Not able to disaggregate specialist addiction services
Substance use among sexual minorities in the US - Linked to inequalities and unmet need for mental health treatment? Results from the National Survey on Drug Use and Health (NSDUH)	Rosner B, Neicun J, Yang JC, Roman-Urrestarazu A. Substance use among sexual minorities in the US - Linked to inequalities and unmet need for mental health treatment? Results from the National Survey on Drug Use and Health (NSDUH). J Psychiatr Res. 2021;135:107-118.	Not about specialist addiction services	
Support, care and peer support for gay and bi men engaging in chemsex	Nagington M, King S. Support, care and peer support for gay and bi men engaging in chemsex. Health Soc Care Community. 2022;30(6):e6396-e6403.	Not about specialist addiction services	Paper focuses on chemsex support via peers/sexual health clinics/inpatient admissions
The Experience of Chemsex and Accessing Medical Care for Gay, Bisexual and Other MSM in Russia: An Interpretive Phenomenological Analysis	Lunchenkov N, Rinne-Wolf S, Hughes T, Dwyer B. The Experience of Chemsex and Accessing Medical Care for Gay, Bisexual and Other MSM in Russia: An Interpretive Phenomenological Analysis. J Sex Res. 2025;62(3):360-366.	Not about specialist addiction services	
The Impacts of Intersecting Stigmas on Health and Housing Experiences of Queer Women Sex Workers in Vancouver, Canada	Lyons T, Krüsi A, Edgar E, Machat S, Kerr T, Shannon K. The Impacts of Intersecting Stigmas on Health and Housing Experiences of Queer Women Sex Workers in Vancouver, Canada. J Homosex. 2021;68(6):957-972.	Not about specialist addiction services	
The management of methamphetamine use in sexual settings among men who have sex with men in Malaysia	Lim SH, Akbar M, Wickersham JA, Kamarulzaman A, Altice FL. The management of methamphetamine use in sexual settings among men who have sex with men in Malaysia. Int J Drug Policy. 2018;55:256-262.	Not about specialist addiction services	

The Need and Desire for Mental Health and Substance Use-Related Services Among Clients of Publicly Funded Sexually Transmitted Infection Clinics in Vancouver, Canada	Salway T, Ferlatte O, Shoveller J, Purdie A, Grennan T, Tan DHS, Consolacion T, Rich AJ, Dove N, Samji H, Scott K, Blackwell E, Mirau D, Holgerson N, Wong J, Gilbert M. The Need and Desire for Mental Health and Substance Use-Related Services Among Clients of Publicly Funded Sexually Transmitted Infection Clinics in Vancouver, Canada. J Public Health Manag Pract. 2019;25(3):E1-E10.	Not about specialist addiction services	Paper aggregates 'mental health and substance use needs' for participants in sexual health context - not able to disaggregate specialist addiction services
The relationship between sexual and gender stigma and difficulty accessing primary and mental healthcare services among LGBTQI+ populations in Thailand: Findings from a national survey	Moallef S, Salway T, Phanuphak N, Kivioja K, Pongruengphant S & Hayashi K. The relationship between sexual and gender stigma and difficulty accessing primary and mental healthcare services among LGBTQI+ populations in Thailand: Findings from a national survey. Int J Ment Health Addiction. 2022;20:3244–3261.	Not about specialist addiction services	
Use of Primary Care and Emergency Departments for Substance Use Treatment: The Rural and Urban Divide	Albright N, Dyar C, Morgan E. Use of Primary Care and Emergency Departments for Substance Use Treatment: The Rural and Urban Divide. Subst Use Misuse. 2024;59(2):300-305.	Not about specialist addiction services	
Using social support for LGBTQ clients with mental illness to be out of the closet, in treatment, and in the community	Klein E. Using social support for LGBTQ clients with mental illness to be out of the closet, in treatment, and in the community. Journal of Gay & Lesbian Social Services. 2017;29(3):221–232.	Not about specialist addiction services	
Utilization of cessation resources among HIV-positive and HIV-negative men who smoke and who have sex with men in Chengdu, China	Berg CJ, Nehl EJ, Wang X, Ding Y, He N, Wong FY. Utilization of cessation resources among HIV-positive and HIV-negative men who smoke and who have sex with men in Chengdu, China. Nicotine Tob Res. 2014;16(10):1283-8.	Not about specialist addiction services	
Utilization of mental health services in relation to the intention to reduce chemsex behavior among clients from an integrated sexual health services center in Taiwan	Hung YR, Chuang TT, Chen TW, Chung AC, Wu MT, Hsu ST, Ko NY, Strong C. Utilization of mental health services in relation to the intention to reduce chemsex behavior among clients from an integrated sexual health services center in Taiwan. Harm Reduct J. 2023;20(1):52.	Not about specialist addiction services	Paper focuses on factors associated with joining chemsex group at specialist chemsex centre rather than specialist addiction services
A comparison of predictors of treatment drop-out of women seeking drug and alcohol treatment in a specialist women's and two traditional mixed-sex treatment services	Copeland J, Hall W. A comparison of predictors of treatment drop-out of women seeking drug and alcohol treatment in a specialist women's and two traditional mixed-sex treatment services. Br J Addict. 1992;87(6):883-90.	Not about barriers/facilitators	
A gay-identified alcohol treatment program: A follow-up study	Driscoll R. A gay-identified alcohol treatment program: a follow-up study. J Homosex. 1982;7(4):71-80.	Not about barriers/facilitators	
Acceptance and commitment therapy approach for problematic chemsex among men who have sex with men	Strika-Bruneau L, Karila L, Amirouche A, Fauvel B, Benyamina A. Acceptance and Commitment Therapy Approach for Problematic Chemsex Among Men Who Have Sex With Men. Cognitive and Behavioral Practice. 2024;31(4):451-468.	Not about barriers/facilitators	
Alcohol and other drug use, club drug dependence and treatment seeking among lesbian, gay and bisexual young people in Sydney	Lea T, Reynolds R, de Wit J. Alcohol and other drug use, club drug dependence and treatment seeking among lesbian, gay and bisexual young people in Sydney. Drug Alcohol Rev. 2013;32(3):303-11.	Not about barriers/facilitators	
Alcohol Use Disorder Treatment in Sexually and Gender Diverse Patients: A Retrospective Cohort Study	McDowell MJ, King DS, Gitin S, Miller AS, Batchelder AW, Busch AB, Greenfield SF, Huskamp HA, Keuroghlian AS. Alcohol Use Disorder Treatment in Sexually and Gender Diverse Patients: A Retrospective Cohort Study. J Clin Psychiatry. 2023;84(5):23m14812.	Not about barriers/facilitators	
Antisocial personality disorder predicts methamphetamine treatment outcomes in homeless, substance-dependent men who have sex with men	Fletcher JB, Reback CJ. Antisocial personality disorder predicts methamphetamine treatment outcomes in homeless, substance-dependent men who have sex with men. J Subst Abuse Treat. 2013;45(3):266-72.	Not about barriers/facilitators	

Are Specialized LGBT Program Components Helpful for Gay and Bisexual Men in Substance Abuse Treatment?	Senreich E. Are specialized LGBT program components helpful for gay and bisexual men in substance abuse treatment? Subst Use Misuse. 2010;45(7-8):1077-96.	Not about barriers/facilitators	More about treatment outcomes than barriers
Asking about sexual orientation during assessment for drug and alcohol concerns: a pilot study	Barbara AM & Chaim G. Asking About Sexual Orientation During Assessment for Drug and Alcohol Concerns: A Pilot Study. Journal of Social Work Practice in the Addictions. 2004;4(4):89–109.	Not about barriers/facilitators	
Assessing the feasibility of SMART Recovery for sexual and gender minority individuals: Results from a feasibility and preliminary outcomes trial	McGeough BL, Cohen NL & Greenwood E. Assessing the feasibility of SMART Recovery for sexual and gender minority individuals: Results from a feasibility and preliminary outcomes trial. Sexual and Gender Diversity in Social Services. 2023;36(3):437–466.	Not about barriers/facilitators	
Availability of Specific Programs and Medications for Addiction Treatment to Vulnerable Populations: Results from the Addiction Treatment Locator, Assessment, and Standards (ATLAS) Survey	Oldfield BJ, Chen K, Joudrey PJ, Biegacki ET, Fiellin DA. Availability of Specific Programs and Medications for Addiction Treatment to Vulnerable Populations: Results from the Addiction Treatment Locator, Assessment, and Standards (ATLAS) Survey. J Addict Med. 2023;17(4):477-480.	Not about barriers/facilitators	
Characteristics of lesbian, gay, bisexual, and transgender individuals entering substance abuse treatment	Cochran BN, Cauce AM. Characteristics of lesbian, gay, bisexual, and transgender individuals entering substance abuse treatment. J Subst Abuse Treat. 2006;30(2):135-46.	Not about barriers/facilitators	
Characteristics of Outpatient and Residential Substance Use Disorder Treatment Facilities with a Tailored LGBT Program	Ware OD, Austin AE, Srivastava A, Dawes HC, Baruah D, Hall WJ. Characteristics of Outpatient and Residential Substance Use Disorder Treatment Facilities with a Tailored LGBT Program. Subst Abuse. 2023;17:11782218231181274.	Not about barriers/facilitators	
Comparison of Government and Non-Government Alcohol and Other Drug (AOD) Treatment Service Delivery for the Lesbian, Gay, Bisexual, and Transgender (LGBT) Community	Mullens AB, Fischer J, Stewart M, Kenny K, Garvey S, Debattista J. Comparison of Government and Non-Government Alcohol and Other Drug (AOD) Treatment Service Delivery for the Lesbian, Gay, Bisexual, and Transgender (LGBT) Community. Subst Use Misuse. 2017;52(8):1027-1038.	Not about barriers/facilitators	Paper focuses on staff attitudes - not specifically conceptualised as barriers
Crystal methamphetamine use subgroups and associated addiction care access and overdose risk in a Canadian urban setting	Brooks O, Bach P, Dong H, Milloy MJ, Fairbairn N, Kerr T, Hayashi K. Crystal methamphetamine use subgroups and associated addiction care access and overdose risk in a Canadian urban setting. Drug Alcohol Depend. 2022;232:109274.	Not about barriers/facilitators	
Demographic Predictors of Dropping Out of Treatment (DOT) in Substance Use Disorder Treatment	Hanauer M, Sielbeck-Mathes K, Banks B, Mitori J, Reuveny A. Demographic Predictors of Dropping Out of Treatment (DOT) in Substance Use Disorder Treatment. Subst Use Misuse. 2021;56(8):1155-1160.	Not about barriers/facilitators	
Differences in assessment and treatment approaches for homosexual clients	MacEwan I. Differences in assessment and treatment approaches for homosexual clients. Drug and Alcohol Review. 1994;13(1):57–62.	Not about barriers/facilitators	Although paper discusses treatment differences between heterosexual and homosexual patients, not specifically conceptualised or elaborated as barriers/facilitators to access
Do specialized services exist for LGBT individuals seeking treatment for substance misuse? A study of available treatment programs	Cochran BN, Peavy KM, Robohm JS. Do specialized services exist for LGBT individuals seeking treatment for substance misuse? A study of available treatment programs. Subst Use Misuse. 2007;42(1):161-76.	Not about barriers/facilitators	
Does sexuality matter? A cross-sectional study of drug use, social injecting, and access to injection-specific care among men who inject drugs in Melbourne, Australia	Schroeder SE, Wilkinson AL, O'Keefe D, Bourne A, Doyle JS, Hellard M, Dietze P and Pedrana A. Does sexuality matter? A cross-sectional study of drug use, social injecting, and access to injection-specific care among men who inject drugs in Melbourne, Australia. Harm Reduction Journal. 2023;20:9.	Not about barriers/facilitators	

Drink goal difficulty effect on outcomes in moderation-based alcohol treatment for sexual minority men	Levak S, Kuerbis AN, Morgenstern J. Drink goal difficulty effect on outcomes in moderation-based alcohol treatment for sexual minority men. J Subst Abuse Treat. 2020;112:1-9.	Not about barriers/facilitators	Paper is more about treatment outcomes than barriers to access
Effects of Sexual Orientation and Gender on Perceived Need for Treatment by Persons With and Without Mental Disorders	Grella CE, Cochran SD, Greenwell L, Mays VM. Effects of sexual orientation and gender on perceived need for treatment by persons with and without mental disorders. Psychiatr Serv. 2011;62(4):404-10.	Not about barriers/facilitators	
Evaluation of an SMS Based Alcohol Intervention for Same Sex Attracted Women: A Randomized Controlled Trial to Examine Feasibility, Acceptability, and Efficacy	Bush R, Staiger PK, McNeill IM, Brown R, Orellana L, Lubman D, McNair R. Evaluation of an SMS Based Alcohol Intervention for Same Sex Attracted Women: A Randomized Controlled Trial to Examine Feasibility, Acceptability, and Efficacy. Subst Use Misuse. 2024;59(8):1157-1166.	Not about barriers/facilitators	
Factors Associated with Exchange Sex Among Cisgender Persons Who Inject Drugs: Women and MSM-23 US Cities, 2018	Rushmore J, Buchacz K, Broz D, Agnew-Brune CB, Jones MLJ, Cha S; NHBS Study Group. Factors Associated with Exchange Sex Among Cisgender Persons Who Inject Drugs: Women and MSM-23 U.S. Cities, 2018. AIDS Behav. 2023;27(1):51-64.	Not about barriers/facilitators	
Gay, Lesbian, Bisexual, and Transgendered People and Chemical Dependency: Exploring Successful Treatment	Rowan NL, Faul AC. Gay, Lesbian, Bisexual, and Transgendered People and Chemical Dependency: Exploring Successful Treatment. Journal of Gay & Lesbian Social Services. 2011;23(1):107–130.	Not about barriers/facilitators	
Governing beyond the closet: Remaking stigma, identity, and sexual behavior in a post-disciplinary rehab	Aleksanyan J. Governing beyond the closet: Remaking stigma, identity, and sexual behavior in a post-disciplinary rehab. Ethnography. 2020;23(4):516-538.	Not about barriers/facilitators	Ethnography including historical analysis, not specifically conceptualised as barriers to access
Health care utilization among young adult injection drug users in Harlem, New York	Cronquist A, Edwards V, Galea S, Latka M, Vlahov D. Health care utilization among young adult injection drug users in Harlem, New York. J Subst Abuse. 2001;13(1-2):17-27.	Not about barriers/facilitators	
Influence of gender, sexual orientation, and need on treatment utilization for substance use and mental disorders: findings from the California Quality of Life Survey	Grella CE, Greenwell L, Mays VM, Cochran SD. Influence of gender, sexual orientation, and need on treatment utilization for substance use and mental disorders: findings from the California Quality of Life Survey. BMC Psychiatry. 2009;9:52.	Not about barriers/facilitators	
Injection and Sexual Behavior Profiles among People Who Inject Drugs in Miami, Florida	Chueng TA, Tookes HE, McLaughlin M, Arcaro-Vinas AM, Serota DP, Bartholomew TS. Injection and Sexual Behavior Profiles among People Who Inject Drugs in Miami, Florida. Subst Use Misuse. 2022;57(9):1374-1382.	Not about barriers/facilitators	
Lesbian, Gay, and Bisexual Clients' Experiences in Treatment for Addiction	Matthews CR, Selvidge MM. Lesbian, gay, and bisexual clients' experiences in treatment for addiction. J Lesbian Stud. 2005;9(3):79-90.	Not about barriers/facilitators	More about factors associated with success of treatment than access to treatment itself
Longitudinal changes in alcohol and drug use among men seen at a gay-specific substance abuse treatment agency	Paul JP, Barrett DC, Crosby GM, Stall RD. Longitudinal changes in alcohol and drug use among men seen at a gay-specific substance abuse treatment agency. J Stud Alcohol. 1996;57(5):475-85.	Not about barriers/facilitators	
Meeting the Needs of Lesbian, Gay, and Bisexual Clients in Substance Abuse Treatment	Flentje A, Livingston NA, Sorensen JL. Meeting the Needs of Lesbian, Gay, and Bisexual Clients in Substance Abuse Treatment. Counselor (Deerfield Beach). 2016;17(3):54-59.	Not about barriers/facilitators	
Mental and Physical Health Needs of Lesbian, Gay, and Bisexual Clients in Substance Abuse Treatment	Flentje A, Livingston NA, Roley J, Sorensen JL. Mental and Physical Health Needs of Lesbian, Gay, and Bisexual Clients in Substance Abuse Treatment. J Subst Abuse Treat. 2015;58:78-83.	Not about barriers/facilitators	

Navigating drug use, cessation, and recovery: a retrospective case notes review among sexual minority men at a community-based service in Singapore	Wah TH, Ong AJX, Naidu KNC, Hanafi S, Tan K, Tan A, Ong TJJ, Ong E, Ho DWS, Subramaniam M, See MY, Tan RKJ. Navigating drug use, cessation, and recovery: a retrospective case notes review among sexual minority men at a community-based service in Singapore. Subst Abuse Treat Prev Policy. 2024;19(1):23.	Not about barriers/facilitators	
Pilot randomized controlled trial of an integrative intervention with methamphetamine-using men who have sex with men	Carrico AW, Gómez W, Siever MD, Discepola MV, Dilworth SE, Moskowitz JT. Pilot randomized controlled trial of an integrative intervention with methamphetamine-using men who have sex with men. Arch Sex Behav. 2015;44(7):1861-7.	Not about barriers/facilitators	
Predictors of moderated drinking in a primarily alcohol-dependent sample of men who have sex with men	Kuerbis A, Morgenstern J, Hail L. Predictors of moderated drinking in a primarily alcohol-dependent sample of men who have sex with men. Psychol Addict Behav. 2012;26(3):484-95.	Not about barriers/facilitators	
Preparing social workers for practice with LGBT populations affected by substance use: perceptions from students, alumni, and service providers	Dentato MP, Kelly BL, Lloyd MR & Busch N. Preparing Social Workers for Practice with LGBT Populations Affected by Substance Use: Perceptions from Students, Alumni, and Service Providers. Social Work Education. 2017;37(3):294–314.	Not about barriers/facilitators	
Queer quit: a pilot study of a smoking cessation programme tailored to gay men	Dickson-Spillmann M, Sullivan R, Zahno B, Schaub MP. Queer quit: a pilot study of a smoking cessation programme tailored to gay men. BMC Public Health. 2014;14:126.	Not about barriers/facilitators	
Reports of alcohol consumption and alcohol-related problems among homosexual, bisexual and heterosexual respondents: Results from the 2000 National Alcohol Survey	Drabble L, Midanik LT, Trocki K. Reports of alcohol consumption and alcohol-related problems among homosexual, bisexual and heterosexual respondents: results from the 2000 National Alcohol Survey. J Stud Alcohol. 2005;66(1):111-20.	Not about barriers/facilitators	
Service Provider Interpretations of Childhood Sexual Experiences Among Sexual Minority Men	Benoit E, Downing MJ, Brown D, Coe L. Service provider interpretations of childhood sexual experiences among sexual minority men. Psychol Serv. 2023;20(Suppl 1):94-102.	Not about barriers/facilitators	
Sexual orientation and substance abuse treatment utilization in the United States: results from a national survey	McCabe SE, West BT, Hughes TL, Boyd CJ. Sexual orientation and substance abuse treatment utilization in the United States: results from a national survey. J Subst Abuse Treat. 2013;44(1):4-12.	Not about barriers/facilitators	
Sexuality and Gender Identity Inequities in Substance Use Disorder and Its Treatment among American Indian, Alaska Native, and Native Hawaiian College Students	Qeadan F, Egbert J, Barbeau WA, Madden EF, Venner KL, English K. Sexuality and Gender Identity Inequities in Substance Use Disorder and Its Treatment among American Indian, Alaska Native, and Native Hawaiian College Students. Subst Use Misuse. 2022;57(14):2085-2093.	Not about barriers/facilitators	
Simulating the experience of searching for LGBTQ-specific opioid use disorder treatment in the United States	Paschen-Wolff MM, Velasquez R, Aydinoglo N, Campbell ANC. Simulating the experience of searching for LGBTQ-specific opioid use disorder treatment in the United States. J Subst Abuse Treat. 2022;140:108828.	Not about barriers/facilitators	
Social and health service use and treatment outcomes for sexual minorities in a national sample of substance abuse treatment programs	Hardesty M, Cao D, Shin HC, Andrews CM, & Marsh J. Social and Health Service Use and Treatment Outcomes for Sexual Minorities in a National Sample of Substance Abuse Treatment Programs. Journal of Gay & Lesbian Social Services. 2012; 24(2):97–118.	Not about barriers/facilitators	
Substance Use, Mental Health, and Service Access among Bisexual Adults in Australia	Loi B, Lea T, & Howard J. Substance Use, Mental Health, and Service Access among Bisexual Adults in Australia. Journal of Bisexuality. 2017;17(4):400–417.	Not about barriers/facilitators	
Substance Use among Lesbian, Gay, and Bisexual Clients Entering Substance Abuse Treatment: Comparisons to Heterosexual Clients	Flentje A, Heck NC, Sorensen JL. Substance use among lesbian, gay, and bisexual clients entering substance abuse treatment: Comparisons to heterosexual clients. J Consult Clin Psychol. 2015;83(2):325-34.	Not about barriers/facilitators	

Substance-related problems and treatment among men who have sex with men in comparison to other men in Chicago	Mackesy-Amiti ME, Fendrich M, Johnson TP. Substance-related problems and treatment among men who have sex with men in comparison to other men in Chicago. J Subst Abuse Treat. 2009;36(2):227-33.	Not about barriers/facilitators	
The 3-Year Course of Multiple Substance Use Disorders in the United States: A National Longitudinal Study	McCabe SE, West BT. The 3-Year Course of Multiple Substance Use Disorders in the United States: A National Longitudinal Study. J Clin Psychiatry. 2017;78(5):e537-e544.	Not about barriers/facilitators	
The availability of LGBT-specific mental health and substance abuse treatment in the United States	Williams ND, Fish JN. The availability of LGBT-specific mental health and substance abuse treatment in the United States. Health Serv Res. 2020;55(6):932-943.	Not about barriers/facilitators	Looking at predictors of services offering specialised LGBT programs
The Availability of Sexual and Gender Minority (SGM) Specific Substance Use Services	Ji CG, Cochran B. The Availability of Sexual and Gender Minority (SGM) Specific Substance Use Services. Subst Use Misuse. 2022;57(14):2126-2133.	Not about barriers/facilitators	
To what extent are key services offered in treatment programs for special populations?	Olmstead T, Sindelar JL. To what extent are key services offered in treatment programs for special populations? J Subst Abuse Treat. 2004;27(1):9-15.	Not about barriers/facilitators	
Treatment counselor's attitudes about lesbian, gay, bisexual, and transgendered clients: Urban vs. rural settings	Eliason MJ, Hughes T. Treatment counselor's attitudes about lesbian, gay, bisexual, and transgendered clients: urban vs. rural settings. Subst Use Misuse. 2004;39(4):625-44.	Not about barriers/facilitators	
Unmet mental health and substance abuse treatment needs of sexual minority elders	Jessup MA, Dibble SL. Unmet mental health and substance abuse treatment needs of sexual minority elders. J Homosex. 2012;59(5):656-74.	Not about barriers/facilitators	
Women Who Use Drugs and Have Sex with Women in a Canadian Setting: Barriers to Treatment Enrollment and Exposure to Violence and Homelessness	Lyons T, Shannon K, Richardson L, Simo A, Wood E, Kerr T. Women Who Use Drugs and Have Sex with Women in a Canadian Setting: Barriers to Treatment Enrollment and Exposure to Violence and Homelessness. Arch Sex Behav. 2016;45(6):1403-10.	Not about barriers/facilitators	
Rapid assessment of drug use and sexual HIV risk patterns among vulnerable drug-using populations in Cape Town, Durban and Pretoria, South Africa	Parry C, Petersen P, Carney T, Dewing S, Needle R. Rapid assessment of drug use and sexual HIV risk patterns among vulnerable drug-using populations in Cape Town, Durban and Pretoria, South Africa. SAHARA J. 2008;5(3):113-9.	Other	Duplicate paper - this paper appears to report on the same data about 78 MSM as in the other included paper by the same authors

Supplementary Table 7: Summary Table of Results

**Service-Related Determinants of Access**	**Barriers**
**Approachability**	APPROPRIATE SERVICES NOT EXISTING<br>• Addiction services being set up for alcohol or non-chemsex drug users rather than for chemsex[88,91,94]<br>• No available inpatient facilities for methamphetamine detoxification, with methamphetamine-using MSM deliberately consuming excessive alcohol to be eligible for detox admission[68]<br>• Services lacking specific expertise around LGBTQ+ addiction issues[67,68,74,81,86–88,91,93,94,100]<br><br>SERVICES NOT WORKING TOGETHER<br>• Fragmentation of services, patients being 'cycled' between services or 'knocking on several doors'[81,90,100]<br><br>SERVICES NOT BEING PUBLICISED<br>• Services failing to provide information about how drug services work and how they can help[89,95]

**Acceptability**	HARM AND DISCRIMINATION<br>• Explicit harassment, discrimination, bullying, threats, violence, or abuse towards SM people within services, including patients being denied treatment due to their sexual identity[43,48,54,57,67,69,73,75,80,104]<br>• Staff failing to protect SM patients from hostility, overt homophobia, attacks, sexual violence, or abuse from non-SM patients in treatment settings[43,48,69,75,80,81]<br>• Explicit negative or stigmatising attitudes of service staff towards SM patients[46,48,57,58,69,75,80,85,86,91,94,105]<br>• Disclosure of SM status whilst accessing or already in treatment negatively affecting subsequent service provision[48,54,67,69,75,80]<br>• For chemsex participants, stigma around accessing needle exchanges and services (because they are associated with opiate drug users)[88]<br><br>UNMET TRAINING NEEDS<br>• Inadequate training/clinical supervision of staff around LGBTQ+ issues, or staff holding inaccurate information/myths/stereotypes about LGBTQ+ people[46,48,68,69,74,80,81,91,104]<br>• Services lacking insight into the unique needs of SM mothers[48,73,93]<br><br>INAPPROPRIATE SERVICE PROCESSES<br>• Heteronormative assumptions made on intake forms and during treatment[48,69,73,80,93]<br>• Drug services having a 'poor reputation'[66]<br>• Services not including the partners of gay/lesbian people in 'family' programmes[73]<br>• Services focusing on abstinence-only approaches[46,67]<br>• Organisational tensions between non-specialist and specialist LGBT programmes within the same service[53]
**Availability and Accommodation**	INCONVENIENT TIMES AND PLACES<br>• Inconvenient opening times (e.g. only 9am-5pm)[42,66,88,90]<br>• Geographical unavailability of services (e.g. outside main city areas)[56,66,78,94]<br>• No clear overall trend of the effect of rurality vs. urbanicity on unmet alcohol and substance use disorder treatment need[62]<br><br>INADEQUATE SERVICE PROVISION<br>• Problems with childcare provision at services[48,86]<br>• Long waiting lists (e.g. six months for a GBL/GHB detox)[66,101]<br>• Low numbers of SM patients leading to isolation within treatment programmes, or restricted provisions of SM-only services[53,54]<br>• Finite/limited resources of services evident (e.g. not giving naloxone to non-injecting drug users)[79]<br>• Staff feeling unable to accommodate the specific needs of SM patients[54]<br>• The type of treatment SM patients wanted not being offered[49]
**Affordability**	INADEQUATE FUNDING OF SERVICES<br>• Low cost or free addiction treatment programmes not being available in certain regions[102]<br>• Under-funded services being unable to pay for additional staff, rooms, or specialist training[53]<br><br>WIDER POLICY AND ECONOMIC FACTORS<br>• Health insurance not covering the cost of addiction treatment[49]<br>• Lack of economic resources preventing access to appropriate addiction-related medical services[46]
**Appropriateness**	LACKING LGBTQ+ CULTURAL COMPETENCE<br>• Service staff being negative/awkward about, or lacking knowledge about, the topic of sexuality and sexual practices[48,54,57,69,74,81,86,91,93,94]<br>• Missed opportunities by services and staff to address or explore important LGBTQ+ issues (such as trauma, coming out, sexualised drug use)[48,54,57,67,69,80,81,91,94,101]<br>• Staff stating that bisexuality did not exist[80]<br>• Staff not understanding the topic of sexual identity and motherhood[93]<br>• Services not acknowledging or addressing intersectionality (e.g. race/ethnicity and sexuality)[48]

	INAPPROPRIATE TREATMENT<br>• An inappropriate under-focus on sexuality (e.g. deflecting the topic even when patients explicitly linked substance use to the stresses associated with being SM)[48,54,69,80,86,94]<br>• An inappropriate over-focus on sexuality, making patients feel misunderstood or "different" (e.g. insisting homosexuality must be the cause of addiction problems)[48,54,69,80]<br>• Staff 'outing' patients without consent and breaching confidentiality[69,80]<br>• Instructing SM patients to hide their sexuality for the sake of non-SM patients in groups[48,54]<br>• Paternalistic, confrontational, or coercive consultation styles[48,69]<br>• Silencing patients who brought up chemsex-related topics[95]<br>• Staff believing that sexuality-conversion therapy was effective or practicing conversion therapy to try and make patients heterosexual[54,80]<br>• Services only targeting a middle-class clientele rather than patients with higher needs[43]

**Service-Related Determinants of Access**	**Facilitators**
**Approachability**	CREATING SPECIFIC SERVICES<br>• Creation or provision of SM-specific services or treatment pathways, including specific needs assessments[53,54,67,69,73,76,78,86,88,93,94,99]<br>• Creation of visibly LGBTQ+ safe/friendly environments (e.g. having LGBTQ+ positive waiting-room literature, or displaying Pride flags or rainbow stickers)[54,69,76,85,91,93]<br><br>SERVICES WORKING TOGETHER<br>• Holistic 'one-stop shop' services which could address addiction, sexual health, and mental health concerns in an integrated way[81]<br>• Partnership working (e.g. between addiction, sexual health, and emergency services, criminal justice agencies, housing providers, and LGBTQ+ organisations)[82,88,100]<br>• Accessing specialist drug services within sexual health clinics (e.g. because they were trusted as gay-friendly)[87,89]<br><br>IMPROVING REFERRAL INTO AND BETWEEN SERVICES<br>• Clear referral pathways into addiction services (e.g. following crisis hospitalisation, from sexual health services, and from public assistance services)[43,88,100,101]<br>• Provision of referral pathway options beyond the criminal justice system[88]<br>• Addiction service staff having knowledge of other local support services to facilitate onward referrals[76,81,91]<br>• Training medical staff (particularly in sexual health and HIV care) in identification and referral of chemsex participants into services[88]<br><br>PUBLICITY AND INFORMATION<br>• Publicity around drug support services directed at MSM[89]<br>• For existing services, outreach to the SM community to facilitate access (e.g. via smartphone hook-up apps, pop-up services, discussion groups, social networks, gay club nights/fetish venues, and commercial sex areas)[43,50,51,88,89,100]<br>• Improved information on pharmacotherapy for alcohol use disorder (AUD) using the language of harm reduction[50]
**Acceptability**	POSITIVE STAFF ATTITUDES AND BEHAVIOURS<br>• Positive, caring, affirmative, or non-judgemental attitudes from service providers (e.g. staff empathising with the difficulties of homophobia, taking time to ensure patients felt comfortable, being able to openly discuss gay sexuality, or acting as LGBTQ+ allies)[54,69,73,75,76,81,82,85,87,89,90,94,100,102]<br>• Service providers having 'cultural humility' or 'horizontal approaches', involving critical self-reflection and positioning the patient as the expert in their lives[69,81,90]<br>• Staff at needle exchange programmes providing informal referrals to less LGBT-hostile service environments, or acting as sources of solidarity[53,69]<br>• Staff explicitly letting patients know that their service is LGB-positive at their first contact[85]<br><br>INCLUSIVE SERVICE PROCESSES AND POLICIES<br>• Staff training (including for non-clinical staff) and policies about SM patients[54,69,73,93]

	• Intake forms/processes using inclusive, open-ended language regarding sexuality and gender, and allowing non-disclosure of SM status[69,93]<br>• Particularly in countries with sanctions around drug use or SM status, services that can be used anonymously[96]<br><br>LGBTQ+ INVOLVEMENT IN SERVICE DESIGN AND DELIVERY<br>• Seeing an openly LGBTQ+ healthcare professional, managerial and organisational support of diversity within drug services, and services recruiting LGBTQ+ staff[47,53,54,69,73,74,76,82,91,94]<br>• Inclusion of LGBTQ+ people in service development (e.g. formulating non-discrimination or staff vetting policies)[69]<br>• Positive LGBTQ+ community feedback enhancing the reputability of services[94]
**Availability and Accommodation**	CONVENIENT TIMES AND PLACES<br>• Establishment of evening and weekend drug clinics, offering alcohol treatment groups in non-clinical settings, and improved service provision in city centres[55,88]<br>• Locating services strategically in LGBTQ+ community areas or areas of high need (e.g. a needle exchange in the gay village, drop-in recovery services near where alcohol is sold)[55,88,89]<br>• For existing services, providing gender neutral bathrooms[53]<br><br>SERVICE DESIGN PREFERENCES<br>• For methamphetamine-using MSM, preferred drug service design characteristics include the availability of one-to-one support in addition to counsellor-led group counselling; long-term, open-ended support; and support located close to home[82]<br>• Having a separate treatment facility or separate unit/area within a service for SM patients[76]<br><br>MULTIPLE SERVICES WORKING TOGETHER<br>• For MSM, high access to substance abuse treatment programmes is typically correlated with high access to HIV risk reduction and education; medical care; mental health services; and a high education level[102]
**Affordability**	Drop-in community recovery centres offering cheap or sliding scale payments[55]
**Appropriateness**	LGBTQ+ COMPETENT TREATMENT<br>• Professionals having specific knowledge, education, and 'cultural competence' about issues relevant to SM healthcare (e.g. methamphetamine use, coming out challenges, societal homophobia, links between drug use and sexual practices)[46,53,54,57,69,73,76,78,81,82,85,94]<br>• Service providers acknowledging sexuality as an important component of identity whilst not assuming it is the cause of a patient's problems[69,73,76,93]<br>• Providers "meeting the client where they are at" (e.g. reflecting patients' own language and identity labels, or offering flexibly tailored treatment)[45,69,76]<br><br>POSITIVE, APPROPRIATE TREATMENT<br>• Open, non-judgemental questioning about gender and sexual preference during intake processes for new patients[53,54,69,73,93]<br>• Matching patients to therapists with their preferred sexual and/or gender identity[53]<br>• Staff using a warm, hopeful consultation style which preserves the patient's decision-making power[48,76,81]<br>• Approaches which are trauma-informed and sex-positive[57,69]<br>• Provision of relevant harm reduction supplies (e.g. clean needles)[69,82,100]<br><br>HOLISTIC APPROACHES<br>• Holistic, intersectional, or 'whole person' approaches to treatment (e.g. incorporating sexuality, substance abuse, housing and social support, subsistence needs, mental health, culture, and spirituality)[48,54,57,69,73,76,81,82,90]<br>• Services being inclusive of HIV education and advocacy[57]<br>• Services having the ability to link patients to wider support programmes (e.g. a SM women's group)[53,81]

Patient-Related Determinants of Access	Barriers
**Ability to Perceive**	NOT KNOWING ABOUT SERVICES<br>• Not knowing that addiction treatment was available/existed[42,50,66,79,94,97]<br>• Not knowing where or how to access services[49,66,79,98]<br><br>NOT WANTING SERVICES<br>• Ambivalence around drug/alcohol use or reluctance to change[42,47,50,58,67,98,101]<br>• Not seeing drug/alcohol use as a problem or serious problem[42,79,89,94,98,101]<br>• Normalisation of drug/alcohol use among SM people/peers[101]<br><br>NOT KNOWING IF SERVICES ARE RIGHT<br>• Not being sure if they would be eligible for treatment[66]<br>• Feeling the type of treatment they wanted was not offered from services[49]<br>• Not believing addiction treatment was appropriate to their needs[48,79]<br>• For lesbians, a sense of being 'different and isolated' leading to a feeling that conventional healthcare is not designed for their needs[48]<br><br>NOT BELIEVING SERVICES COULD HELP<br>• Negative beliefs around addiction or its treatment (e.g. feeling ‘I should be strong enough to handle this alone’, not believing that ‘alcoholism’ is a treatable condition, or feeling that GBL/GHB users were treated inadequately by services)[42,66,97,98,101]<br>• Concerns about other drug users, or rehabilitation itself, triggering relapse[67,79,98]<br><br>DRUG USE PREVENTING SERVICE ACCESS<br>• For MSM, greater frequency of methamphetamine use, and greater frequency of sexualised use, is associated with an increased perceived difficulty of accessing support[84]
**Ability to Seek**	FEAR OF NEGATIVE OR PUNITIVE EXPERIENCES<br>• Fears or embarrassment about discussing addiction or treatment[42,66,73]<br>• Apprehension about discussing sexualised drug or alcohol use[75,79,87,89]<br>• Worrying about involuntary admission to hospital if services are accessed[42]<br>• For lesbians, fears that children will be taken into care if they enter treatment services[48]<br>• Fear of incarceration/criminal sanctions for drug use or gay sex, or being reported by staff to drug law enforcement agencies [95,96]<br>• Not accessing services due to fears of legal ramifications[94]<br><br>PREVIOUS NEGATIVE EXPERIENCES<br>• Previous unsuccessful attempts to access or utilise treatment[42,98,101]<br><br>CONCERNS ABOUT STIGMA<br>• Fears of others’ opinions on learning about a person’s addiction problems[42,73]<br>• Fear of stigma due to SM status, alcohol/drug use (particularly injecting), or addiction treatment itself [48,50,56,69,70,73,75,76,79,85,86,94–96]<br>• Concerns around being given pathologising labels (e.g. ‘alcoholic’) compounding the social stigma already experienced[45,48,86]<br><br>NEEDING COMMITMENT<br>• For services that are difficult to physically access, an increased commitment is needed to attend[56]

	SPECIFIC 'MINORITY-WITHIN-A-MINORITY' FACTORS<br>• For Black non-gay identified men who have sex with men and women, not seeking treatment due to cultural constructions of masculinity[45]<br>• For Black MSM, barriers to methamphetamine treatment are a racialised problem: caused by exposure to poverty, inadequate education, and few resources compared with white MSM[68]<br>• For gay/lesbian-specific treatment units, a focus on white men can mean that they are perceived as less welcoming for women and non-white people[76]<br>• Women experiencing lifetime high risk drug use were more likely than moderate or low risk users to report that they had wanted but had not received professional support[44]<br>• Compared with gay men, bisexual men were reported to have both a decreased63,65 and increased83 likelihood of drug treatment utilisation, and an increased likelihood of alcohol treatment utilisation[71]<br>• Compared with a matched cohort of heterosexual men, SM men ≥50 years old had a lower rate of inpatient drug treatment utilisation, and compared with heterosexual women, SM women ≥50 years old had a higher rate of inpatient drug treatment utilisation[72]<br>• Black bisexual women had higher odds of reporting a treatment gap for both specialty drug, and alcohol services (compared with Black bisexual men, gay men, or lesbian women), and Black gay men had worse odds of experiencing a drug specialty treatment gap than Black bisexual men[59]<br>• Latina SM women were less likely to access substance use treatment services compared with white SM women[77]<br>• Compared with SM men, SM women with probable AUD were less likely to have this diagnosed by a professional, and less likely to attend substance use-related appointments[60]
**Ability to Reach**	LACKING APPROPRIATE FAMILY OR SOCIAL SUPPORT<br>• Black lesbian and bisexual women have less social support to help with accessing alcohol treatment than for heterosexual women[52]<br>• For MSM, family members forcing them enter treatment may create resistance to the idea[67]<br><br>TRANSPORT DIFFICULTIES<br>• A car may be required to access services, or patients may need to pay others to drive them[56]
**Ability to Pay**	INDIVIDUAL FINANCIAL FACTORS<br>• Being unable to afford medical bills/pay for addiction services, or concerns about the potential costs[42,46,56,66]<br>• For medications for AUD, concerns around cost to benefits ratio of medications[50]<br><br>WIDER POLICY FACTORS<br>• Problems with obtaining health insurance, which may be dependent on employment[56,78]
**Ability to Engage**	IDENTITY CONCEALMENT AND ALIENATION<br>• Patients feeling the need to present in a heteronormative manner[80]<br>• Patients feeling the need to censor/not disclose their sexuality[75,85,94]<br>• Treatment being unsuccessful or less successful if someone feels unable to disclose their sexuality or sexual practices[75,85,94]<br>• Feelings of isolation or alienation (i.e. as a minority within predominantly heterosexual) services or therapeutic groups[67,75,76]<br><br>FEAR OR MISTRUST OF HEALTHCARE PROVIDERS<br>• Mistrust in healthcare providers (e.g. patients feeling that healthcare providers have negative/pathologising attitudes or inadequate knowledge, or they have to educate their healthcare providers about LGBTQ+ related issues)[48,69,85,94]<br>• Patients fearing homophobia, shaming, rejection, or abuse based on their sexual identity within treatment[54,69,75,76,86,94]<br>• Difficulties discussing sexual practices or identity in therapeutic relationships, or fearing that staff members cannot 'relate' to SM patients[54,69,75,85,89,94]<br><br>INDIVIDUAL MOTIVATION FACTORS<br>• Patients not wanting to engage with services, or wanting to continue alcohol/drug use[42]<br>• Difficulty staying sober because of being unable to enjoy sex without drugs[46]<br>• For medication for AUD, concerns around the burden of treatment, and fears that it might negatively impact enjoyment of alcohol[50,97]<br><br>PRACTICAL AND CONFIDENTIALITY CONCERNS<br>• Not having time to attend a service[66]

	• Difficulty in keeping appointments during periods of heavy substance use[56]<br>• Concerns with confidentiality when accessing services[66,73,94]

Patient-Related Determinants of Access	Facilitators
**Ability to Perceive**	KNOWING ABOUT SERVICES<br>• Knowledge of addiction support services and treatment options (e.g. pharmacological treatment of AUD)[50,101]<br><br>FINDING INFORMATION ABOUT SERVICES<br>• Peer advocates and LGBTQ+ networks helping patients to understand their addiction problems and promoting treatment[43,58,67]<br><br>HAVING MOTIVATION TO CHANGE<br>• For chemsex participants, motivation to access services may occur when they perceive it to be detrimental, or to avoid related financial, housing, or employment crises[94]
**Ability to Seek**	No reported facilitators
**Ability to Reach**	LGBTQ+ PEERS AND NETWORKS FACILITATING SERVICE ENTRY<br>• Signposting and referral to services through LGBTQ+ community contacts, networks, peer advocates, family and friends[43,58,67,101]<br>• For MSM, use of substance abuse treatment programmes is positively associated with connection to the gay community[102]<br><br>HAVING FAMILY SUPPORT<br>• Family support viewed as important for successful treatment[97]<br>• Gay/lesbian people being able to bring 'whoever you want to be considered family' into family meetings as part of treatment[73]<br><br>CRIMINAL SYSTEM INVOLVEMENT<br>• SM individuals with a history of criminal legal system involvement are more likely to utilise drug treatment than SM individuals without this history[64]
**Ability to Pay**	FLEXIBILITY IN PAYING FOR SERVICES<br>• Patients using private services (paid for directly or by insurance) even when free public healthcare was available, because of faster access or needing expertise not available in the public health sector[81]<br>• State addiction treatment funding being able to be used to access an LGBTQ-specific treatment centre[78]<br><br>NOMINAL FEES<br>• Providing AUD treatment for a nominal fee to allow for more buy-in with the idea of treatment[97]<br><br>HAVING SIMILAR PEERS<br>• For MSM, the importance of other participants in drug treatment being in similar financial situations to themselves[82]
**Ability to Engage**	HAVING SPECIFIC MINORITY-WITHIN-MINORITY SERVICES<br>• For SM women, having specialist women-only services is associated with greater likelihood of completing treatment[92]<br>• Having specialist female-only or lesbian-only groups allows SM women to discuss specific relevant issues (e.g. sexual harassment and gender roles)[55,86]<br><br>PEER SUPPORT AND LGBTQ+ COMMUNITY CONNECTION<br>• Peer support around GHB/GBL use for MSM[101]<br>• Building opportunities for socialising, self-expression, community connection, and mutual support (particularly with other SM people) within treatment[67,73,75,76,82]<br>• The importance of SM role models who were in recovery from addiction themselves; both other patients and staff[73,94]<br>• Having other treatment participants who identify as SM or LGBTQ+[69,73,75,76,82]

	ENCOURAGING AUTHENTICITY, FOREGROUNDING AUTONOMY<br>• For gay and lesbian patients, being able to 'work though shame to self-acceptance'[73]<br>• Focus of treatment being use reduction or harm reduction rather than abstinence, or allowing patients to set their own goals[55,67,69,82,97]<br>• Being able to be honest and open about the specific context of sexuality and addiction[73,75,76]<br><br>NON-CLINICAL SETTINGS<br>• For individuals with mild or moderate alcohol problems, they may be more likely to engage in a group in a non-clinical setting[55]<br><br>SPECIFIC RECOVERY FACILITATORS<br>• Both SM and heterosexual participants reported that their top addiction recovery facilitators were friends, community and network; family and children; and 12-step programmes such as Alcoholics Anonymous. More SM than heterosexual participants cited spirituality, education, and loss (e.g. of identity, loved ones, agency) as facilitators to recovery[61]

Supplementary Table 8: PRISMA Checklist

Section and Topic	Item #	Checklist item	Location where item is reported (page number)
**TITLE**			
Title	1	Identify the report as a systematic review.	1
**ABSTRACT**			
Abstract	2	See the PRISMA 2020 for Abstracts checklist.	1
**INTRODUCTION**			
Rationale	3	Describe the rationale for the review in the context of existing knowledge.	2
Objectives	4	Provide an explicit statement of the objective(s) or question(s) the review addresses.	2
**METHODS**			
Eligibility criteria	5	Specify the inclusion and exclusion criteria for the review and how studies were grouped for the syntheses.	3, OSM
Information sources	6	Specify all databases, registers, websites, organisations, reference lists and other sources searched or consulted to identify studies. Specify the date when each source was last searched or consulted.	3
Search strategy	7	Present the full search strategies for all databases, registers and websites, including any filters and limits used.	OSM
Selection process	8	Specify the methods used to decide whether a study met the inclusion criteria of the review, including how many reviewers screened each record and each report retrieved, whether they worked independently, and if applicable, details of automation tools used in the process.	4
Data collection process	9	Specify the methods used to collect data from reports, including how many reviewers collected data from each report, whether they worked independently, any processes for obtaining or confirming data from study investigators, and if applicable, details of automation	4

Section and Topic	Item #	Checklist item	Location where item is reported (page number)
		tools used in the process.	
Data items	10a	List and define all outcomes for which data were sought. Specify whether all results that were compatible with each outcome domain in each study were sought (e.g. for all measures, time points, analyses), and if not, the methods used to decide which results to collect.	4, OSM
	10b	List and define all other variables for which data were sought (e.g. participant and intervention characteristics, funding sources). Describe any assumptions made about any missing or unclear information.	4, OSM
Study risk of bias assessment	11	Specify the methods used to assess risk of bias in the included studies, including details of the tool(s) used, how many reviewers assessed each study and whether they worked independently, and if applicable, details of automation tools used in the process.	4
Effect measures	12	Specify for each outcome the effect measure(s) (e.g. risk ratio, mean difference) used in the synthesis or presentation of results.	N/A
Synthesis methods	13a	Describe the processes used to decide which studies were eligible for each synthesis (e.g. tabulating the study intervention characteristics and comparing against the planned groups for each synthesis (item #5)).	4
	13b	Describe any methods required to prepare the data for presentation or synthesis, such as handling of missing summary statistics, or data conversions.	4
	13c	Describe any methods used to tabulate or visually display results of individual studies and syntheses.	N/A
	13d	Describe any methods used to synthesize results and provide a rationale for the choice(s). If meta-analysis was performed, describe the model(s), method(s) to identify the presence and extent of statistical heterogeneity, and software package(s) used.	4
	13e	Describe any methods used to explore possible causes of heterogeneity among study results (e.g. subgroup analysis, meta-regression).	N/A
	13f	Describe any sensitivity analyses conducted to assess robustness of the synthesized results.	N/A
Reporting bias assessment	14	Describe any methods used to assess risk of bias due to missing results in a synthesis (arising from reporting biases).	4
Certainty assessment	15	Describe any methods used to assess certainty (or confidence) in the body of evidence for an outcome.	4
**RESULTS**			
Study selection	16a	Describe the results of the search and selection process, from the number of records identified in the search to the number of studies included in the review, ideally using a flow diagram.	4
	16b	Cite studies that might appear to meet the inclusion criteria, but which were excluded, and explain why they were excluded.	OSM
Study characteristics	17	Cite each included study and present its characteristics.	5
Risk of bias in	18	Present assessments of risk of bias for each included study.	5, OSM

Section and Topic	Item #	Checklist item	Location where item is reported (page number)
studies			
Results of individual studies	19	For all outcomes, present, for each study: (a) summary statistics for each group (where appropriate) and (b) an effect estimate and its precision (e.g. confidence/credible interval), ideally using structured tables or plots.	5-12
Results of syntheses	20a	For each synthesis, briefly summarise the characteristics and risk of bias among contributing studies.	5
	20b	Present results of all statistical syntheses conducted. If meta-analysis was done, present for each the summary estimate and its precision (e.g. confidence/credible interval) and measures of statistical heterogeneity. If comparing groups, describe the direction of the effect.	N/A
	20c	Present results of all investigations of possible causes of heterogeneity among study results.	N/A
	20d	Present results of all sensitivity analyses conducted to assess the robustness of the synthesized results.	N/A
Reporting biases	21	Present assessments of risk of bias due to missing results (arising from reporting biases) for each synthesis assessed.	5, OSM
Certainty of evidence	22	Present assessments of certainty (or confidence) in the body of evidence for each outcome assessed.	5, OSM
**DISCUSSION**			
Discussion	23a	Provide a general interpretation of the results in the context of other evidence.	11-12
	23b	Discuss any limitations of the evidence included in the review.	12
	23c	Discuss any limitations of the review processes used.	12
	23d	Discuss implications of the results for practice, policy, and future research.	13
**OTHER INFORMATION**			
Registration and protocol	24a	Provide registration information for the review, including register name and registration number, or state that the review was not registered.	3
	24b	Indicate where the review protocol can be accessed, or state that a protocol was not prepared.	3
	24c	Describe and explain any amendments to information provided at registration or in the protocol.	N/A
Support	25	Describe sources of financial or non-financial support for the review, and the role of the funders or sponsors in the review.	13
Competing interests	26	Declare any competing interests of review authors.	14
Availability of data, code and other materials	27	Report which of the following are publicly available and where they can be found: template data collection forms; data extracted from included studies; data used for all analyses; analytic code; any other materials used in the review.	OSM

Online Supplementary Material

Online Supplementary Material........1
Supplementary Table 1: Inclusion and Exclusion Criteria........1
Supplementary Table 2: Search Syntaxes........2
Supplementary Table 3: Data Items Extracted........5
Supplementary Table 4: Included Studies........6
Supplementary Table 5: MMAT-2018 Responses for Included Studies........20
Supplementary Table 6: Excluded Studies........24
Supplementary Table 7: Summary Table of Results........38
Supplementary Table 8: PRISMA Checklist........45

Supplementary Table 1: Inclusion and Exclusion Criteria

	INCLUSION	EXCLUSION
**COVERAGE**	Articles reporting on adults (>80% of participants in study are ≥18 years)<br>AND<br>Studies published in English<br>AND<br>Published in any country	Articles reporting solely on patients <18 years<br>OR<br>Studies not published in English
**POPULATION**	Studies that report on the population defined above<br>OR<br>Studies that include findings from health and social work professionals, or service providers, involved in the care of the above population, which are relevant to the study aims	Studies that report only on heterosexual participants<br>OR<br>Studies where barriers/facilitators to treatment cannot be disaggregated for sexual minorities and heterosexual participants<br>OR<br>Studies reporting solely on gender minorities (e.g. trans or non-binary individuals)

**OUTCOMES**	Studies reporting on barriers to access for addiction treatment services (i.e. that are delivering specialist addiction-related healthcare such as medical assessment, pharmacotherapy, psychological support, detox, needle exchange, facilitated treatment groups) for the above population<br>OR<br>Studies reporting on facilitators to access for addiction treatment services for the above population	Studies reporting only on barriers/facilitators to access for peer-support or community-organised groups such as Alcoholics Anonymous<br>OR<br>Studies reporting on access barriers/facilitators to general mental health or medical services or primary care services (not specifically addiction services)<br>OR<br>Studies based exclusively on clinical management or outcomes for this population
**STUDY DESIGN/TYPE**	Qualitative, quantitative, or mixed-methods primary research studies	Editorials, reviews, case reports, comment articles, news articles, books, or conference abstracts<br>OR<br>Papers reporting solely on methods/instruments, with no research findings relevant to the study question
**QUALITY**	Passed MMAT-2018 screening questions	Did not pass MMAT-2018 screening questions
**OTHER**	Full-text available	Full-text not available

Supplementary Table 2: Search Syntaxes

DATABASE	SEARCH SYNTAX OR STRATEGY
MEDLINE	((("gay"[Title/Abstract] OR "lesbian*"[Title/Abstract] OR "LGB"[Title/Abstract] OR "queer*"[Title/Abstract] OR "msm"[Title/Abstract] OR "wsw"[Title/Abstract] OR "homosex*"[Title/Abstract] OR "pansex*"[Title/Abstract] OR "GLB"[Title/Abstract] OR "sexual minorit*"[Title/Abstract] OR "same sex attract*"[Title/Abstract] OR "non heterosexual*"[Title/Abstract] OR "men who have sex with men"[Title/Abstract] OR "women who have sex with women"[Title/Abstract] OR ("homosexuality"[MeSH Terms] OR "bisexuality"[MeSH Terms])) AND ("addict*"[Title/Abstract] OR "alcohol*"[Title/Abstract] OR "ivdu*"[Title/Abstract] OR "heroin"[Title/Abstract] OR "opioid"[Title/Abstract] OR "cocaine"[Title/Abstract] OR "methamphetamine"[Title/Abstract] OR "MDMA"[Title/Abstract] OR "ecstasy"[Title/Abstract] OR "GHB"[Title/Abstract] OR "GBL"[Title/Abstract] OR "hallucinogen*"[Title/Abstract] OR "cannabis"[Title/Abstract] OR "marijuana"[Title/Abstract] OR "drinking"[Title/Abstract] OR "injecting"[Title/Abstract] OR "substance misuse"[Title/Abstract] OR "substance use"[Title/Abstract] OR "substance abuse"[Title/Abstract] OR "illicit drug"[Title/Abstract] OR "illegal drug"[Title/Abstract] OR "drug misuse"[Title/Abstract] OR "drug abuse"[Title/Abstract] OR "drug use"[Title/Abstract] OR ("alcoholism"[MeSH Terms] OR "substance related disorders"[MeSH Terms] OR "drug seeking behavior"[MeSH Terms])) AND ("rehab*"[Title/Abstract] OR "service*"[Title/Abstract] OR "psychiatr*"[Title/Abstract] OR "CMHT"[Title/Abstract] OR "counsel*"[Title/Abstract] OR "GP"[Title/Abstract] OR "psychotherap*"[Title/Abstract] OR "detox*"[Title/Abstract] OR "treatment*"[Title/Abstract] OR "intervention*"[Title/Abstract] OR "therap*"[Title/Abstract] OR "mental health*"[Title/Abstract] OR "primary care"[Title/Abstract] OR "health team*"[Title/Abstract] OR ("substance abuse treatment centers"[MeSH Terms] OR "needle exchange programs"[MeSH Terms] OR "mental health services"[MeSH Terms] OR "addiction medicine"[MeSH Terms])) AND ("accepta*"[Title/Abstract] OR "access*"[Title/Abstract] OR

	"barrier*"[Title/Abstract] OR "challenge*"[Title/Abstract] OR "engage*"[Title/Abstract] OR "exclu*"[Title/Abstract] OR "hindrance*"[Title/Abstract] OR "assist*"[Title/Abstract] OR "limitation*"[Title/Abstract] OR "obstacle*"[Title/Abstract] OR "pathway*"[Title/Abstract] OR "utilisation*"[Title/Abstract] OR "utilization*"[Title/Abstract] OR "helpseeking"[Title/Abstract] OR "uptake*"[Title/Abstract] OR "facilitat*"[Title/Abstract] OR "furtherance"[Title/Abstract] OR "disparit*"[Title/Abstract] OR "treatment gap"[Title/Abstract] OR "help-seeking"[Title/Abstract] OR "help seeking"[Title/Abstract] OR "support-seeking"[Title/Abstract] OR "support seeking"[Title/Abstract] OR "seeking help"[Title/Abstract] OR "seeking support"[Title/Abstract] OR ("patient acceptance of health care"[MeSH Terms] OR "health services accessibility"[MeSH Terms] OR "help seeking behavior"[MeSH Terms])) AND "English"[Language] AND "humans"[MeSH Terms]) NOT ("Comment"[Publication Type] OR "Editorial"[Publication Type] OR "meta analysis"[Publication Type] OR "News"[Publication Type] OR "Review"[Publication Type] OR "Systematic Review"[Publication Type] OR "cochrane"[Title/Abstract] OR "literature review"[Title] OR "Systematic Review"[Title])) AND (alladult[Filter])
PSYCHInfo (via Ovid)	#1 gay.ti,ab. or lesbian*.ti,ab. or LGB.ti,ab. or queer*.ti,ab. or msm.ti,ab. or wsw.ti,ab. or homosex*.ti,ab. or pansex*.ti,ab. or GLB.ti,ab. or sexual minorit*.ti,ab. or same sex attract*.ti,ab. or non heterosexual*.ti,ab. or men who have sex with men.ti,ab. or women who have sex with women.ti,ab. or (exp homosexuality/ or exp bisexuality/)<br><br>#2 addict*.ti,ab. or alcohol*.ti,ab. or ivdu*.ti,ab. or heroin.ti,ab. or opioid.ti,ab. or cocaine.ti,ab. or methamphetamine.ti,ab. or MDMA.ti,ab. or ecstasy.ti,ab. or GHB.ti,ab. or GBL.ti,ab. or hallucinogen*.ti,ab. or cannabis.ti,ab. or marijuana.ti,ab. or drinking.ti,ab. or injecting.ti,ab. or substance misuse.ti,ab. or substance use.ti,ab. or substance abuse.ti,ab. or illicit drug.ti,ab. or illegal drug.ti,ab. or drug misuse.ti,ab. or drug abuse.ti,ab. or drug use.ti,ab. or (exp alcoholism/ or exp substance related disorders/ or exp drug seeking behavior/)<br><br>#3 rehab*.ti,ab. or service*.ti,ab. or psychiatr*.ti,ab. or CMHT.ti,ab. or counsel*.ti,ab. or GP.ti,ab. or psychotherap*.ti,ab. or detox*.ti,ab. or treatment*.ti,ab. or intervention*.ti,ab. or therap*.ti,ab. or mental health*.ti,ab. or primary care.ti,ab. or health team*.ti,ab. or (exp substance abuse treatment centers/ or exp needle exchange programs/ or exp mental health services/ or exp addiction medicine/)<br><br>#4 accepta*.ti,ab. or access*.ti,ab. or barrier*.ti,ab. or challenge*.ti,ab. or engage*.ti,ab. or exclu*.ti,ab. or hindrance*.ti,ab. or assist*.ti,ab. or limitation*.ti,ab. or obstacle*.ti,ab. or pathway*.ti,ab. or utilisation*.ti,ab. or utilization*.ti,ab. or helpseeking.ti,ab. or uptake*.ti,ab. or facilitat*.ti,ab. or furtherance.ti,ab. or disparit*.ti,ab. or treatment gap.ti,ab. or help-seeking.ti,ab. or help seeking.ti,ab. or support-seeking.ti,ab. or support seeking.ti,ab. or seeking help.ti,ab. or seeking support.ti,ab. or (exp patient acceptance of health care/ or exp health services accessibility/ or exp help seeking behavior/)<br><br>#5 exp Comment/ or exp Editorial/ or exp meta analysis/ or exp News/ or exp Review/ or exp Systematic Review/ or cochrane.ti,ab. or literature review.ti,bt. or Systematic Review.ti,bt.<br><br>(1 AND 2 AND 3 AND 4) NOT 5 with limits set of 'English', 'Adult'
CINAHL (via EBSCO*Host*)	#1 TI (gay or lesbian* or LGB or queer* or msm or wsw or homosex* or pansex* or GLB or "sexual minorit*" or "same sex attract*" or "non heterosexual" or "men who have sex with men" or "women who have sex with women") or AB (gay or lesbian* or LGB or queer* or msm or wsw or

	homosex* or pansex* or GLB or "sexual minorit*" or "same sex attract*" or "non heterosexual" or "men who have sex with men" or "women who have sex with women") or (MH "Gay Persons+") or (MM "Bisexuals") or (MM "Gay Men") or (MM "Bisexuality")<br><br>#2 TI (addict* or alcohol* or ivdu* or heroin or opioid or cocaine or methamphetamine or MDMA or ecstasy or GHB or GBL or hallucinogen* or cannabis or marijuana or drinking or injecting or "substance misuse" or "substance use" or "substance abuse" or "illicit drug" or "illegal drug" or "drug misuse" or "drug abuse" or "drug use") or AB (addict* or alcohol* or ivdu* or heroin or opioid or cocaine or methamphetamine or MDMA or ecstasy or GHB or GBL or hallucinogen* or cannabis or marijuana or drinking or injecting or "substance misuse" or "substance use" or "substance abuse" or "illicit drug" or "illegal drug" or "drug misuse" or "drug abuse" or "drug use") or (MH "Substance Use Disorders+") or (MH "Alcohol Abuse+") or (MH "Alcoholism") or (MH "Drug-Seeking Behavior")<br><br>#3 TI (rehab* or service* or psychiatr* or CMHT or counsel* or GP or psychotherap* or detox* or treatment* or intervention* or therap* or "mental health*" or "primary care" or "health team*" or "needle exchange*" or AB (rehab* or service* or psychiatr* or CMHT or counsel* or GP or psychotherap* or detox* or treatment* or intervention* or therap* or "mental health*" or "primary care" or "health team*" or "needle exchange*") or (MH "Substance Use Rehabilitation Programs+") or (MM "Needle Exchange Programs") or (MH "Mental Health Services+") or (MM "Addictions Nursing")<br><br>#4 TI (accepta* or access* or barrier* or challenge* or engage* or exclu* or hindrance* or assist* or limitation* or obstacle* or pathway* or utilisation* or utilization* or helpseeking or uptake* or facilitat* or furtherance or disparit* or "treatment gap" or "help-seeking" or "help seeking" or "support-seeking" or "support seeking" or "seeking help" or "seeking support") or AB (accepta* or access* or barrier* or challenge* or engage* or exclu* or hindrance* or assist* or limitation* or obstacle* or pathway* or utilisation* or utilization* or helpseeking or uptake* or facilitat* or furtherance or disparit* or "treatment gap" or "help-seeking" or "help seeking" or "support-seeking" or "support seeking" or "seeking help" or "seeking support") or (MH "Health Services Accessibility+") or (MM "Help Seeking Behavior")<br><br>#5 TI (Cochrane or "literature review" or "systematic review" or "meta analysis") or AB (Cochrane or "literature review" or "systematic review" or "meta analysis")<br><br>(1 AND 2 AND 3 AND 4) NOT 5 with limits set of 'English', 'Humans', 'All Adult', 'Journal Article'
Web of Science	(((TI=(gay or lesbian* or LGB or queer* or msm or wsw or homosex* or pansex* or GLB or "sexual minorit*" or "same sex attract*" or "non heterosexual" or "men who have sex with men" or "women who have sex with women")) or (AB=(gay or lesbian* or LGB or queer* or msm or wsw or homosex* or pansex* or GLB or "sexual minorit*" or "same sex attract*" or "non heterosexual" or "men who have sex with men" or "women who have sex with women"))) AND ((TI=(addict* or alcohol* or ivdu* or heroin or opioid or cocaine or methamphetamine or MDMA or ecstasy or GHB or GBL or hallucinogen* or cannabis or marijuana or drinking or injecting or "substance misuse" or "substance use" or "substance abuse" or "illicit drug" or "illegal drug" or "drug misuse" or "drug abuse" or "drug use")) or (AB=(addict* or alcohol* or ivdu* or heroin or opioid or cocaine or methamphetamine or MDMA or ecstasy or GHB or GBL or hallucinogen* or cannabis or marijuana or drinking or injecting or "substance misuse" or "substance use" or "substance abuse" or "illicit drug" or "illegal drug" or "drug misuse" or "drug abuse" or "drug use"))) AND ((TI=(rehab* or service* or psychiatr* or CMHT or counsel* or GP or psychotherap* or detox* or treatment* or intervention* or therap* or "mental health*" or "primary care"

	or "health team*" or "needle exchange*")) or (AB=(rehab* or service* or psychiatr* or CMHT or counsel* or GP or psychotherap* or detox* or treatment* or intervention* or therap* or "mental health*" or "primary care" or "health team*" or "needle exchange*"))) AND ((TI=(accepta* or access* or barrier* or challenge* or engage* or exclu* or hindrance* or assist* or limitation* or obstacle* or pathway* or utilisation* or utilization* or helpseeking or uptake* or facilitat* or furtherance or disparit* or "treatment gap" or "help-seeking" or "help seeking" or "support-seeking" or "support seeking" or "seeking help" or "seeking support")) or (AB=(accepta* or access* or barrier* or challenge* or engage* or exclu* or hindrance* or assist* or limitation* or obstacle* or pathway* or utilisation* or utilization* or helpseeking or uptake* or facilitat* or furtherance or disparit* or "treatment gap" or "help-seeking" or "help seeking" or "support-seeking" or "support seeking" or "seeking help" or "seeking support")))) NOT ((TI=(Cochrane or "literature review" or "systematic review" or "meta analysis")) or (AB=(Cochrane or "literature review" or "systematic review" or "meta analysis"))) with limits set for 'English' and 'Journal Article'
Sociological Abstracts (via ProQuest)	#1 TITLE,ABSTRACT(gay or lesbian* or LGB or queer* or msm or wsw or homosex* or pansex* or GLB or "sexual minorit*" or "same sex attract*" or "non heterosexual" or "men who have sex with men" or "women who have sex with women") or MAINSUBJECT.EXACT.EXPLODE("Homosexuality") or MAINSUBJECT.EXACT.EXPLODE("Bisexuality")<br><br>#2 TITLE,ABSTRACT(addict* or alcohol* or ivdu* or heroin or opioid or cocaine or methamphetamine or MDMA or ecstasy or GHB or GBL or hallucinogen* or cannabis or marijuana or drinking or injecting or "substance misuse" or "substance use" or "substance abuse" or "illicit drug" or "illegal drug" or "drug misuse" or "drug abuse" or "drug use") or MAINSUBJECT.EXACT.EXPLODE("Drug Abuse") or MAINSUBJECT.EXACT.EXPLODE("Alcoholism") or MAINSUBJECT.EXACT.EXPLODE("Drug Addiction") or MAINSUBJECT.EXACT.EXPLODE("Substance Abuse")<br><br>#3 TITLE,ABSTRACT(rehab* or service* or psychiatr* or CMHT or counsel* or GP or psychotherap* or detox* or treatment* or intervention* or therap* or "mental health*" or "primary care" or "health team*" or "needle exchange*") or MAINSUBJECT.EXACT.EXPLODE("Needle Exchange Programs") or MAINSUBJECT.EXACT.EXPLODE("Mental Health Services")<br><br>#4 TITLE,ABSTRACT(accepta* or access* or barrier* or challenge* or engage* or exclu* or hindrance* or assist* or limitation* or obstacle* or pathway* or utilisation* or utilization* or helpseeking or uptake* or facilitat* or furtherance or disparit* or "treatment gap" or "help-seeking" or "help seeking" or "support-seeking" or "support seeking" or "seeking help" or "seeking support") or MAINSUBJECT.EXACT.EXPLODE("Help Seeking Behavior")<br><br>#5 TITLE,ABSTRACT(Cochrane or "literature review" or "systematic review" or "meta analysis")<br><br>(#1 AND #2 AND #3 AND #4) NOT #5 with limits set of 'Article' and 'English'

Supplementary Table 3: Data Items Extracted

The following key variables were extracted from identified full-text studies into Microsoft Excel using a standardised form:

Data Item	Description
Study identifier	First author and year of publication, study title
Type of study	Qualitative, quantitative, or mixed-methods
Country/countries that study is reporting data from	Name of country
Research methods and objectives of the study	Brief summary description of study indicating key objectives and methods
Total number of study patients/participants reported on	Number
Type of addiction treatment service reported on (e.g. statutory health services, private health services, third-sector providers, residential vs. non-residential settings)	Description of type of service
Barriers to access	Categorised into the dimensions of accessibility according to the model from Levesque et al., 'service side' factors (approachability, acceptability, availability & accommodation, affordability, appropriateness) and 'patient side' factors (ability to perceive, ability to seek, ability to reach, ability to pay, ability to engage)
Facilitators to access	Categorised into the dimensions of accessibility according to the model from Levesque et al., 'service side' factors (approachability, acceptability, availability & accommodation, affordability, appropriateness) and 'patient side' factors (ability to perceive, ability to seek, ability to reach, ability to pay, ability to engage)
(If data available) mean age of sample	Years
(If data available) numbers of gay vs. lesbian vs. bisexual vs. other SM participants in the sample	Reported using language of each paper
(If data available) gender identity of participants	Reported using language of each paper
(If data available) ethnic/racial identity of participants	Reported using language of each paper

Supplementary Table 4: Included Studies

First author and year of publication	Full title	Country the data are from	Overall number of participants the study	Number of sexual minority participants in the study relevant to questions of the review	Study description and methodology	Type of addiction treatment service reported on (e.g. statutory health services, private health services, third-sector providers, residential vs. non-residential settings)	Mean age of SM people within sample (years)	Sexual identity of SM people in sample (number of participants)	Gender identity of SM people in sample (number of participants)	Race/ethnicity of SM people within sample (number of participants)
Allen (2016)	Sexual orientation, treatment utilization, and barriers for alcohol related problems: Findings from a nationally	USA	11,182	53	Secondary analysis of representative population survey (NESARC) on alcohol-related conditions, comparing heterosexual and sexual minority respondents' ratings of	Alcohol or drug detox, 12-step meetings including AA/NA, family/social service, inpatient wards, outpatient clinics, rehab programmes, A&E, crisis	Not reported	Gay or lesbian 31, bisexual 22	Gay/lesbian: 60.3% male and 39.7% female<br><br>Bisexual: 32.3% male and 67.7% female	Gay/lesbian: White non-Hispanic 73.2%, Black/African American 6.9%, American Indian/Alaskan

	representative sample				barriers to treatment access	centres, employee assistance programmes, clergyman/priest/rabbi, private professional, other agencies				Native 5.7%, Asian/Pacific Islander 3.5%, Hispanic (any race) 10.8%<br><br>Bisexual: White non-Hispanic 76.8%, Black/African American 12.3%, American Indian/Alaskan Native 2.0%, Asian/Pacific Islander 0.9%, Hispanic (any race) 7.9%
Aslan (2024)	Harm reduction and multidisciplinary consultations for gay, bisexual, and other men who have sex with men practising chemsex based in a French infectious disease unit: patients' characteristics and perceptions	France	172	96	Cross-sectional survey assessing sociodemographic and clinical characteristics of gay, bisexual, and other men who have sex with men (MSM) involved in chemsex who attended a specific chemsex harm reduction programme	Establishment of a specific chemsex treatment/harm reduction programme within an infectious diseases unit	41	Gay, bisexual, or other men who have sex with men 96	Men 96	Unknown
Barbara (2002)	Substance abuse treatment with lesbian, gay and bisexual people: A qualitative study of service providers	Canada	26	18 (study of service providers)	Qualitative study of service providers' knowledge, involving focus groups and individual interviews with clinicians experienced in treating LGB clients with substance abuse problems	All participants in the study delivering LGB substance abuse treatment via addiction centres, counselling centres or private practice	Not reported specifically for SM, of all participants: 43 (range 30-64)	Gay or lesbian 18	Not reported specifically for SM, of all participants: female 17, male 9	Unknown
Batchelder (2021)	Mental Health and Substance Use Diagnoses and Treatment Disparities by Sexual Orientation and Gender in a Community Health Center Sample	USA	24,325	13,648	Quantitative analysis of data from electronic medical records at health centre for sexual and gender minority patients, comparing mental health and addiction diagnoses, and treatment attendance, between sexual orientation and gender categories	Substance abuse treatment services at a community health centre	32.1	Gay men 8652, gay/lesbian women 1718, bisexual men 771, bisexual women 1189, other men 640, other women 678	Cisgender men 9141, cisgender women 2745, transgender men 615, transgender women 742	Race: Native American, Pacific Islander or Other 382, Asian or Asian American 687, Black or African American 786, Multiracial 743, White 10,321<br><br>Ethnicity: Latinx 754
Bernier (2024)	Examining sexual minority engagement	USA	337	68	Mixed methods study: quantitative analysis comparing heterosexual	Addiction recovery community centres offering e.g. relapse	40.34	Non-sexual or asexual 7, homosexual, gay or lesbian 22, bisexual 30,	Cisgender male 24, cisgender female 41,	Race: White or Caucasian 49, Black or African

	in recovery community centers				and sexual minority participants' utilisation of addiction recovery community centres, qualitative analysis of recovery facilitators	prevention, case management, employment assistance, support groups		questioning, curious or not sure 8, other 3	transgender male 2, other 1	American 15, Native Indian or Alaskan Native 1<br><br>Ethnicity: Hispanic or Latino 9, Not Hispanic 59
Blanchette (2024)	Sexualized substance use among gbMSM: Their perspectives on their intervention needs and counsellor competencies	Canada	20	20	Qualitative semi-structured interviews with gay, bisexual, or other MSM with addiction treatment experience, aiming to identify sexualised substance use intervention needs, analysed using descriptive/interpretive approach within frameworks of sexual health and addiction counsellor competencies	Substance use treatment programs	37.6 (range 23-60)	Gay 17, bisexual 1, pansexual 1, queer 1	Cisgender male 19, trans male 1	Ethnocultural background: Canadian 18, Latino 2
Bourne (2015)	"Chemsex" and harm reduction need among gay men in South London	UK	30	30	Qualitative interviews with gay men involved in the chemsex scene in South London, analysed using thematic analysis	Specialist drug services	36 (range 21-53)	Gay men 30	Men 30	White British 16, White other 8, White Irish 3, Black Caribbean 1, Other 2
Braine (2014)	Sexual Minority Women Who Use Drugs: Prejudice, Poverty, and Access to Care	USA	34	34	Qualitative semi-structured interviews with sexual minority female heroin or crack cocaine users in New York City, analysed using grounded theory	Inpatient drug treatment programs, syringe/needle exchange programmes, methadone maintenance programmes	Not reported (range 20-60+)	Lesbian/gay 19, women who have sex with women (WSW) 5, bisexual 6, primarily heterosexual WSW 4	Female 34, of which 1 assigned male at birth<br><br>Gender conformity: 'normative gender identity' 23, 'non conforming gender identity' 34	African American 17, Latina (Puerto Rico and Dominican Republic) 5, White 7, Mixed 5
Brown (2017)	Men Who Have Sex With Men in Peru: Acceptability of Medication-Assisted Therapy for Treating Alcohol Use Disorders	Peru	26	20 (sexual identity of remaining participants unspecified)	Qualitative study using three focus groups about acceptability of medication assisted therapy for alcohol use disorder in Lima, analysed using thematic analysis	Medication assisted therapy for AUD	28.3 (range 20-40)	Male: homosexual 8, bisexual 5, heterosexual MSM 4, Unspecified 3<br><br>Transgender women: 6 (sexual identity unspecified)	Men 20, trans women 6	Unknown
Card (2021)	Perceived difficulty of getting help to reduce or abstain from substances among sexual and gender minority men	Canada	376	376	Quantitative online survey among methamphetamine-using SGMSM examining demographic, clinical, and social predictors of	Addiction treatment, counselling, or harm reduction services	42.00	Men, trans men, or non-binary people who have sex with men 376, of which non-gay identified 88	Cisgender men 352, trans or non-binary 24	White 248, Person of Colour 104

	who have sex with men (SGMSM) and use methamphetamine during the early period of the COVID-19 pandemic				perceived difficulty in getting help with substance use during the initial phases of the COVID-19 pandemic					
Card (2022)	Does Treatment Readiness Shape Service-Design Preferences of Gay, Bisexual, and Other Men Who Have Sex with Men Who Use Crystal Methamphetamine? A Cross Sectional Study	Canada	291	291	Cross-sectional survey of gay, bisexual, and other MSM who use methamphetamine, examining the association between readiness-to-change (assessed using SOCRATES-8D scale) and relative importance of different addiction service design characteristics	Study assessing theoretical addiction service design characteristics; participants asked about 'professional help to control, cut down, or stop using crystal methamphetamine'	Median 41 (Q1-Q3 34-51)	MSM 291, of which gay 231, bisexual 34, other 26	Cisgender men 263, trans or non-binary 22	Person of Colour 83, Not Person of Colour 208
Cochran (2007)	Substance abuse treatment providers' explicit and implicit attitudes regarding sexual minorities	USA	46	11	Survey of substance abuse treatment providers' implicit and explicit attitudes regarding LGBT clients (measures of heterosexist/homophobic bias), quantitative analysis of measures of implicit and explicit bias	All specialist substance abuse treatment counsellors	Not reported specifically for SM, of all participants: 46.3	Gay/lesbian 7, bisexual 3, other 1	Not reported specifically for SM, of all participants: 52% female	Not reported specifically for SM, of all participants: Caucasian 22, Latino/a 9, African American 5, Asian American 4, Multi-racial 4, Other 2
Conner (2023)	Access and Barriers to Health Services Among Sexual and Gender Minority College Students	USA	1892	406	Online survey comparing cisgender, heterosexual with sexual and gender minority college students, assessing demographics, mental health and addiction characteristics, service utilisation, and barriers to service access	Substance use services	Not reported, but majority (68.9%) of all participants in age range 18-20	Gay or lesbian 63, bisexual or pansexual 246, questioning or bicurious 54, asexual or different identity 43	Female sex assigned at birth 325	Minority race or ethnicity 91
Copeland (1992)	A comparison of women seeking drug and alcohol treatment in a specialist women's and two traditional mixed-sex treatment services	Australia	320	27 lesbian; bisexual women also included in study but numbers aggregated with heterosexual women	Characteristics of 160 women who left specialist addiction services <5 days from admission compared to 160 women who stayed in specialist addiction services 5 or more days from admission; specialist addiction services were comprised of a specialist women's centre with residential childcare and a traditional mixed sex treatment service	Specialist drug treatment services	Not reported specifically for SM, of all participants: 29.7 (left services) and 30.8 (continued in services), range 17-66	Lesbian 27; bisexual women also included in study but numbers aggregated with heterosexual women	Women 27	Unknown

Corliss (2006)	Drug use, drug severity, and help-seeking behaviors of lesbian and bisexual women	USA	2011	2011	Self-administered cross-sectional questionnaire of sexual minority women in the San Francisco Bay Area and Los Angeles County, assessing demographics, drug use information, social/emotional problems, and help-seeking behaviours	Professional substance use services compared to self-help services	Not reported (range 18-55+)	Lesbian 82.8%, bisexual 11.2%, other non-heterosexual 6.0%	Women 2011	White 66.7%, African American 10.2%, Latina 13.2%, Asian/Pacific Islander 6.7%, Other 3.1%
Dyar (2023)	Rural and urban differences in disparities in substance use and substance use disorders affecting sexual minority populations	USA	210392	15007	Quantitative analysis of data from 2015-2019 National Survey of Drug Use and Health (NSDUH), comparing sexual minority to heterosexual disparities in substance use, substance use disorders, and treatment need as a function of urbanicity/rurality	Substance use treatment or counselling (for alcohol and/or drugs)	Not reported	Lesbian female 2292, bisexual female 8017, gay male 2348, bisexual male 2350	Female 10309, male 4698	Lesbian female: Non-Hispanic White (NHW) 63.0%, Non-Hispanic Black (NHB) 15.7%, Hispanic (H) 15.0%, Non-Hispanic Asian (NHA) 2.7%, Non-Hispanic Other Race (NHOR) 3.6%<br><br>Bisexual female: NHW 60.8%, NHB 13.8%, H 6.2%, NHA 44.4%, NHOR 4.8%<br><br>Gay male: NHW 61.5%, NHB 11.3%, H 19.4%, NHA 4.6%, NHOR 3.1%<br><br>Bisexual male: NHW 59.8%, NHB 10.1%, H 20.0%, NHA 7.0%, NHOR 3.1%
Flores (2017)	Availability and Use of Substance Abuse Treatment Programs Among Substance-Using Men Who Have Sex With Men Worldwide	Global	3478	3478	Global, cross-sectional online survey of substance-using MSM aiming to explore substance abuse treatment programme availability and use, and behavioural and demographic correlates of treatment programme use	Free or low-cost substance abuse treatment programmes	35	MSM 3478	Men 3478	Minority racial/ethnic category within country in which they reside 891, not a minority racial/ethnic category within country in which they reside 2640

Forenza (2016)	Exploring Service Provider Perceptions of Treatment Barriers Facing Black, Non-Gay-Identified MSMW	USA	18	Not reported (study of service providers)	Qualitative study involving two focus groups in New Jersey and New York City of 18 individuals from HIV/AIDS and substance abuse service providers, focusing on Black Non-Gay-Identified men who have sex with men and women (NGI-MSMW), analysed using grounded theory methods	Outpatient counselling, residential treatment, harm reduction services, detox and medical treatment for substance use disorders	Not reported (study of service providers)	Not reported (study of service providers)	Not reported (study of service providers)	Not reported (study of service providers)
Freestone (2025)	Exploring experiences of talk therapies among gay and bisexual men seeking to reduce or abstain from using crystal methamphetamine in the context of chemsex	Australia	24	24	Qualitative interview study with gay and bisexual MSM and non-binary people with chemsex experience, focusing on access to counselling or therapy around drug use, analysed using qualitative descriptive methodology	Therapeutic and psychological support for drug use, including an outpatient hospital stimulant treatment programme, LGBTQ+ drug counselling programme, drug support from a psychologist or counsellor, drug counselling via sexual health clinics or LGBTQ+ organisations	Not reported	Gay 21, bisexual 3, queer 1	Cisgender male 22, trans male 1, non-binary person 1	Caucasian 21, Asian 3, Latinx 1
Goodyear (2020)	"Am I gonna get in trouble for acknowledging my will to be safe?": Identifying the experiences of young sexual minority men and substance use in the context of an opioid overdose crisis	Canada	50	50	Semi-structured interviews with 50 sexual minority men who use substances in Vancouver, Canada, explicitly exploring how patterns and contexts of substance use are linked to the opioid overdose crisis	Harm reduction and drug use treatment services	24.18 (range 18-30)	Gay 29, bisexual 14, pansexual 6, queer 4, straight 2, asexual 1, unsure 1, other 2<br><br>(Note some participants selected more than one sexual identity)	Cisgender man 44, transgender man 4, queer 4, genderqueer 2, non-binary 1<br><br>(Note some participants selected more than one gender identity)	White 26, Aboriginal 10, Latin American 7, South Asian 5, South East Asian 2, Chinese 2, Korean 2, Filipino 2, Arab/West Asian 2, Black 1, Japanese 1, Other 2<br><br>(Note some participants selected more than one racial identity)
Gorman (1997)	Speed, Sex, Gay Men, and HIV: Ecological and Community Perspectives	USA	30	30	Exploratory ethnographic study of MSM who use drugs in the Seattle area using ethnographic observations, focus groups and individual interviews	Drug treatment programme	Not reported (range 20-56)	MSM or trans women having sex with men 30, of which majority gay, about 1/6 bisexual, 3 trans women, some declined to specify sexual orientation	Not fully reported but all MSM or trans women	Approx 80% White, 20% included Native American, Latino, African American and Mixed-Race individuals
Graf (2018)	Chemsex among men who have sex with	Germany	116	89	Qualitative study of drug-using MSM and health	Multidisciplinary chemsex support	Not reported (range 22-64)	MSM 89	Men 89	Not specified, though study

	men in Germany: motives, consequences and the response of the support system				professionals' perspectives on chemsex, using individual interviews and analysed using content analysis	services (including medical care, psychosocial and drug advice, sexual therapy, inpatient withdrawal treatment, harm reduction)				notes 'nearly none of them [participants] had a migratory background'
Green (2011)	Barriers and treatment preferences reported by worried drinkers of various sexual orientations	USA	218	62	Online convenience survey of worried alcohol drinkers, comparing heterosexual vs. LGB respondents, asking about barriers to treatment access and addiction treatment preferences	Professional inpatient treatment, professional outpatient treatment, self-help groups, computerised treatment, self-help books	Not reported	Lesbian women 34, bisexual women 12, bisexual men 2, gay men 14	Male 16, female 46	Unknown
Hall (1994)	Lesbians recovering from alcohol problems: an ethnographic study of health care experiences	USA	35	35	Qualitative semi-structured ethnographic interviews with lesbian problem alcohol drinkers, analysed using grounded theory and critical feminist approaches	Outpatient specialist addiction services and barriers to accessing formal addiction treatments	37 (range 24-54)	Lesbian 35	Female 35	Euro-American 24, African American 6, Latina 3, Asian/Pacific 1, Native American 1
Haney (2021)	Sexual Orientation, Social Determinants of Health, and Unmet Substance Use Treatment Need: Findings from a National Survey	USA	988	175	Analysis of data from representative population survey (National Survey of Drug Use and Health) comparing heterosexual with gay/lesbian/bisexual respondents regarding perceived reasons for unmet substance use treatment need	Speciality treatment facilities for illicit drug/alcohol use, including inpatient hospitals, drug/alcohol rehabilitation facilities (in/outpatient) and mental health centres	Not reported	Lesbian, gay, bisexual 175 (no disaggregated data)	Unknown	Unknown
Hellman (1989)	Treatment of homosexual alcoholics in government-funded agencies: provider training and attitudes	USA	164	13 (study of service providers)	Descriptive quantitative survey of service providers in government-funded agencies in New York City, focusing on their training experiences, competencies, and attitudes towards treating homosexual alcoholics	Government-funded alcohol treatment agencies	Not reported specifically for SM, of all participants: mean not reported, age ranges 18-24 = 4, 25-34 = 39, 35-44 = 60, 45-54 = 42, 55-64 = 12, 65+ = 2	Bisexual 3, homosexual 10 (service providers)	Not reported specifically for SM, of all participants: male 64, female 94	Not reported specifically for SM, of all participants: Asian 6, Black 48, Hispanic 26, White 73, Other 3
Hodges (2023)	Sexual minorities and substance use treatment utilization: New evidence from a national sample	USA	21,926	2871	Quantitative study using data from the 2015-2019 National Survey on Drug Use and Health (NSDUH) to examine the association between sexual identity, gender, and substance use	Substance use disorder treatment for alcohol, illicit drugs, and prescription medication misuse (including residential,	Not reported in detail, though age included as potential confounder	Gay men 487, bisexual men 453, lesbian women 342, bisexual women 1589	Male 940, female 1931	Not reported in detail, though race/ethnicity included as potential confounder

					treatment utilisation, controlling for demographic covariates	inpatient, outpatient, and self-help groups)				
Hsiang (2018)	Acceptability of pharmacotherapy for hazardous alcohol use among men who have sex with men: Findings from a qualitative study	USA	39	39	Qualitative focus group discussions with MSM from San Francisco Bay area who had consumed alcohol in the last year, analysed using content analysis	Pharmacological treatment for hazardous drinking	39.1 (range 23-66)	MSM 39	Male 39	Asian/Pacific Islander 10, Black/African American 9, Latino 6, Mixed 4, White 10
Hussen (2021)	Exploring influences on methamphetamine use among Black gay, bisexual and other men who have sex with men in Atlanta: A focus group study	USA	54	36	Qualitative study using thematic analysis of eight focus groups of key informants from community-based and healthcare organisations working with Black gay, bisexual, and other MSM, exploring trends in methamphetamine use and the associated risk environment	Substance use treatment, including harm reduction facilities	Not reported specifically for SM, of all participants: mean not specified, age ranges 20-30 = 22, 31-40 = 15,<br>41-50 = 8,<br>51+ = 7<br><br>(Note two participants missing demographic data)	Gay/same-gender loving 29, bisexual 4, other 3	Not reported specifically for SM, of all participants: cisgender man 35, cisgender woman 14, transgender woman 2, gender non-conforming person 1<br><br>(Note two participants missing demographic data)	Not reported specifically for SM, of all participants: Black 45, White 3, Other 4<br><br>(Note two participants missing demographic data)
Israelstam (1988)	Knowledge and opinions of alcohol intervention workers in Ontario, Canada, regarding issues affecting male gays and lesbians: Parts I and II	Canada	85	Not reported (study of service providers)	Quantitative mailed, anonymous survey to assess the knowledge, attitudes, and experiences of alcohol intervention workers, with a focus on the treatment of gay and lesbian clients	Alcohol treatment or intervention services	Not reported specifically for SM, of all participants: median 40, range 25-63	Not reported	Not reported specifically for SM, of all participants: male 39, female 44, unknown 2	Not reported
Jeong (2016)	Racial/ethnic differences in unmet needs for mental health and substance use treatment in a community-based sample of sexual minority women	USA	699	699	Quantitative analysis of cross-sectional data from the Chicago Health and Life Experiences of Women (CHLEW) study, employing logistic regression to examine associations between sexual identity, race/ethnicity, and use of mental health and substance use treatment while controlling for various demographic factors	Substance use treatment	Not reported, however age ranges: ≤30 = 225, 31-40 = 149, 41-50 = 141, ≥51 = 184	Lesbian 517, bisexual 182	Women 699	Latina 162, White 261, African American 252

Joyce (2018)	The Experience and Meaning of Problematic 'G' (GHB/GBL) Use in an Irish Context: an Interpretative Phenomenological Analysis	Ireland	7	5	Qualitative semi-structured interviews with participants attending a community drug team for support with problematic GHB/GBL use, analysed using interpretative phenomenological analysis	Specialist drug service for problematic GHB/GBL use	36.2 (range 31-39)	Gay 5	Male 5	White 5
Kanouse (2005)	Recruiting drug-using men who have sex with men into behavioral interventions: a two-stage approach	USA	83	83	Quantitative study comparing methods of recruiting drug-using MSM into behavioural interventions (such as motivational interviewing or contingency management) - 2 stage (recruiting into discussion groups then offering intervention) vs. 1 stage (offering intervention)	Drug use behavioural interventions	Not reported	Gay 53, bisexual 25, other 5	Male 83	African-American 23, White 32, Hispanic 17, Asian/Pacific Islander 2, unspecified 9
Krasnova (2021)	Disparities in substance use disorder treatment use and perceived need by sexual identity and gender among adults in the United States	USA	21,926	2871	Quantitative study of data from the 2015-2019 National Survey on Drug Use and Health (NSDUH) using weighted multivariable logistic regression models to examine sexual orientation differences in treatment utilisation and perceived need for substance use treatment, adjusting for socio-demographic characteristics	Study examined both 'any substance use disorder (SUD) treatment' (any service utilisation in outpatient and inpatient settings, such as a hospital, rehabilitation facility, emergency room, mental health centre, doctor's office, prison or jail, self-help services such as Alcoholics Anonymous and Narcotics Anonymous, or "some other place") and 'specialty SUD treatment', (treatment in an inpatient hospital, drug or alcohol rehabilitation centre (inpatient or outpatient), or mental health centre offering specialty treatments)	Not reported	Gay men 487, bisexual men 453, gay/lesbian women 342, bisexual women 1589	Men 940, women 1931	Gay men: Non-Hispanic white (NHW) 59.2%, Non-Hispanic Black (NHB) 15.5%, Hispanic (H) 20.1%, Other (O) 5.3%<br><br>Bisexual men: NHW 63.3%, NHB 9.7%, H 20.6%, O 6.5%<br><br>Gay/lesbian women: NHW 57.1%, NHB 17.0%, H 18.2%, O 7.6%<br><br>Bisexual women: NHW 61.6%, NHB 15.1%, H 15.7%, O 7.7%
Matthews (2006)	Treatment Experiences of Gays and Lesbians In Recovery from Addiction: A Qualitative Inquiry	USA	10	10	Qualitative interview study of gay men and lesbians' experiences of treatment and recovery from drug and/or alcohol addiction,	Addiction treatment programs including inpatient, outpatient, partial hospitalisation, and 12-step programs	46.9 (range 31-69)	Lesbian/dyke 6, gay male/homosexual 4	Women 6, men 4	European American 9, African American 1

					analysed using thematic analysis					
Mays (1994)	Perceived social support for help-seeking behaviors of Black heterosexual and homosexually active women alcoholics	USA	70 (of whom 59 specified their sexuality)	21	Cross-sectional self-administered questionnaire of African American women in alcoholism treatment centres asking about social support and help seeking behaviours	Inpatient alcoholism treatment centres	31 (range 20-68)	Lesbian 12, bisexual 9	Female 21	African-American 21
McGaughey (2023)	Harm reduction services for people engaging in chemsex in Brighton, UK: A pilot qualitative study	UK	10 (service providers)	Not reported	Qualitative semi-structured interview study of commissioners, managers, or providers of chemsex harm reduction services, analysed using thematic analysis	Harm reduction services for chemsex	Not reported	Not reported	Not reported	Not reported
Mericle (2018)	Delivering LGBT-sensitive substance use treatment to sexual minority women	USA	11	Not reported (study of service providers)	Qualitative semi-structured elite interviews with directors of substance use treatment programmes in the San Francisco Bay area, focusing on how they address the needs of sexual minority women, analysed using qualitative framework approach	Residential and outpatient specialist substance use addiction services	Not reported (study of service providers)	Not reported (study of service providers)	Female 9, male 2	Not reported (study of service providers)
Micale (2024)	Examining alcohol use disorder treatment utilization in lesbian, gay, and bisexual populations through the Behavioral Model for Vulnerable Populations	USA	1152	1152	Quantitative analysis of data from 2012-2013 National Epidemiologic Survey on Alcohol and Related Conditions-III (NESARC-III), analysed using logistic regression based on the Behavioral Model for Vulnerable Populations, to assess the impact of predisposing factors, enabling factors, and treatment need on alcohol treatment use among LGB participants	Professional alcohol use disorder services (including family services or other social service agencies, alcohol/drug detoxification wards/clinics, inpatient wards of psychiatric/general hospital or community mental health programs, outpatient clinics, alcohol/drug rehabilitation programs, halfway houses/therapeutic communities, private physicians, psychiatrists, psychologists, or social workers, any other professional or agency)	37.56	Gay male 321, lesbian woman 265, bisexual man 144, bisexual woman 422	Female 687, male 465	White, non-Hispanic 622, Black, non-Hispanic 266, American Indian/Alaska Native, non-Hispanic 20, Asian/Native Hawaiian/Other Pacific Islander, non-Hispanic 28, Hispanic (any race) 216

Nieder (2025)	Minority Stress, Substance Use, and Resilience: Examining Long-Term Sobriety in the LGBTQ Community	USA	16	14	Qualitative interview study with LGBTQ people in long-term recovery (2-24 years) from substance use disorders, focusing on their paths to recovery, analysed using thematic analysis	Professional drug support services including inpatient and outpatient substance use programs, sober living programs, therapy, LGBTQ-specific drug treatment programs	35.38 (range 20-69)	Gay 7, lesbian 2, bisexual 1, pansexual 2, queer 2	Cisgender male 6, cisgender female 5, non-binary 2, genderfluid 1	White 7, Latinx 3, Black 1, Black/White 1, Latinx/White 1, Native American/White 1
Parry (2008)	Rapid assessment of drug-related HIV risk among men who have sex with men in three South African cities	South Africa	78	78	Qualitative study using rapid assessment methodology including mapping, observations, key informant interviews and focus groups with 78 drug-using MSM in Pretoria, Durban and Cape Town	Drug treatment centres and drug rehabilitation centres	For key informant interviews: 28.9, (range 18-50) For focus group: not reported (range 19-55)	MSM 78	Men 78	African Black 13, Coloured 23, Indian 3, White 39
Paschen-Wolff (2024)	Experiences of and recommendations for LGBTQ plus - affirming substance use services: an exploratory qualitative descriptive study with LGBTQ plus people who use opioids and other drugs	USA	23	23	Qualitative study using individual interviews with LGBTQ+ people, exploring experiences of substance use treatments and generating recommendations for affirming care, analysed using thematic analysis	Substance use treatment services and related services (e.g. syringe exchange services and 12-step programs)	27.5 (range 21-38)	Bisexual 5, gay 4, lesbian 4, prefer not to specify 1, pansexual 2, queer 7	Cisgender man 4, cisgender woman 8, non-binary 4 (two of whom also identified as transgender), transgender man 6, transgender woman 1	Black/African American (non-Hispanic) 7, Latinx (any race) 4, Multiracial (non-Hispanic) 3, White (non-Hispanic) 9
Penn (2013)	LGBTQ persons with co-occurring conditions: Perspectives on treatment	USA	10	10	Qualitative semi-structured focus group exploring barriers, needs and experiences of LGBTQ people living with substance abuse and mental illness, analysed using grounded theory methods	Therapeutic programme to address substance abuse and mental health conditions	Not reported (range 22-51)	Lesbian 2, gay 3, bisexual 2, transgendered 1, lesbian/bisexual 1, gay/bisexual 1	Male 5, female 5	White 5, Hispanic 2, Native American 2, Black 1
Pennay (2018)	Improving alcohol and mental health treatment for lesbian, bisexual and queer women: Identity matters	Australia	25	25	Qualitative semi-structured interviews with sexual minority women about alcohol and mental health treatment, analysed thematically using a grounded theory approach	Alcohol and substance use treatment services	40.1 (range 19-71)	Lesbian 11, bisexual 5, gay 2, queer 2, pansexual 2, did not wish to be labelled 3	Female 22, transgender 1, intersex 1, genderqueer 1	Race/ethnicity not described but country of birth provided: Australia 18 (1 Aboriginal), UK 3, New Zealand 1, USA 1, Russia 1, Singapore 1
Ralphs (2018)	New psychoactive substances: new service provider challenges	UK	84	15 chemsex users - all assumed to be LGBTQ, no information given about	Qualitative semi-structured face to face interviews with 53 users of new psychoactive substances (including 15 chemsex users) and 31 key	Specialist addiction services for problematic users of new psychoactive substances	Not reported	MSM 15	Assumed to be men 15	Not reported

				sexual identity of other participants	stakeholders (e.g. drug service providers) in Manchester, no information provided on the qualitative analysis method					
Ramakrishnan (2023)	Sexual Orientation, Mental Illness, and Substance Use Disorders Among Criminal Legal System-Involved Individuals	USA	195,239	14,995	Quantitative study using data from the 2015-2019 National Survey of Drug Use and Health, comparing sexual minority and heterosexual adults with and without criminal legal system involvement, using multivariable logistic regression, and controlling for sociodemographic characteristics	Substance use disorder treatment services	Not reported	Criminal legal involvement: bisexual 68.5%, gay or lesbian 31.5%<br>No criminal legal involvement: bisexual 61.5%, gay or lesbian 38.5%	Criminal legal involvement: male 39.0%, female 61.0%<br>No criminal legal involvement: male 39.2%, female 60.8%	Criminal legal involvement: Non-Hispanic White (NHW) 51.1%, Non-Hispanic Black (NHB) 22.6%, Non-Hispanic Other (NHO) 6.4%, Hispanic (H) 19.9%<br>No criminal legal involvement: NHW 64.9%, NHB 13.0%, NHO 4.0%, H 18.1%
Rice (2024)	Risk factors of substance use treatment gaps among a nationally representative sample of Black American adults in relation to sexual minority status and health insurance coverage	USA	36,098	Not specifically reported, but 5.9% of the sample gay/lesbian or bisexual	Quantitative analysis of cross-sectional data from 2015-2019 National Survey on Drug Use and Health, examining self-reported substance use treatment gaps for Black Americans	Speciality treatment facilities for illicit drug/alcohol use, including inpatient hospitals, drug/alcohol rehabilitation facilities (in/outpatient) and mental health centres	Not reported (59.9% of total sample ≥35)	Of total sample: bisexual men 0.8%, bisexual women 2.6%, gay men 1.0%, lesbian women 1.1%	Of total sample: 45.8% men, 54.2% women	All of sample Black
Roth (2021)	Longitudinal Analysis of HIV Risk and Substance Use Patterns for Men Who Have Sex With Men and Women and Men Who Have Sex With Men Only	Canada	771	771	Quantitative analysis of data from prospective cohort study in British Columbia, comparing HIV risk and substance use between men who have sex with men only (MSMO) and men who have sex with men and women (MSMW), using multivariable logistic regression	Treatment for substance abuse	MSMO: median 34 (Q1-Q3 26-47)<br>MSMW: median 31 (Q1-Q3 23-40)	Gay 655, bisexual 73, queer/other 46	Male 759, female to male transgender 14, male to female transgender 1	MSMO: White 537, Asian 74, Indigenous 45, Latino/Other 57<br>MSMW: White 47, Asian 0, Indigenous 3, Latino/Other 8
Rowan (2013)	What Is Valued in Gay and Lesbian Specific Alcohol and Other Drug Treatment	USA	40	39	Qualitative study using individual interviews with 24 'alumni' (former clients) and 16 clinical staff of gay-specific addiction treatment programs, analysed using thematic	Gay-specific alcohol and other drug treatment programs	Alumni: 42 (range 33-68)<br>Clinical staff: 44 (range 26-60)	Alumni: gay male 23, lesbian 1<br>Clinical staff: gay male 10, bisexual male 1, lesbian 3, bisexual female 1	Alumni: male 23, female 1<br>Clinical staff: male 11, female 4	Alumni: Caucasian 17, Black 2, Hispanic 1, East Indian 1, Multiriacial 1<br>Clinical staff: Caucasian 15

					analysis and the recovery capital framework					
Rowan (2022)	Health Disparities Between Older Lesbian, Gay, and Bisexual Adults and Heterosexual Adults in the Public Mental Health System	USA	3118	1659	Quantitative study of electronic health records, comparing a matched sample of lesbian, gay, and bisexual adults ≥50 years with a heterosexual sample, examining disparities in health and service use	Inpatient stays related to substance use (including detoxification services, rehabilitation services, and other substance use-related stays)	Not reported (all study participants ≥50)	Gay and bisexual men 844, lesbian and bisexual women 815	Men 844, women 815	Men: White Non-Hispanic 457, Black Non-Hispanic 176, Hispanic 181 Women: White Non-Hispanic 332, Black Non-Hispanic 269, Hispanic 186
Saulnier (1997)	Alcohol problems and marginalization: Social group work with lesbians	USA	12	12	Mixed-methods evaluation of a social work intervention group for lesbian women with alcohol problems, including quantitative assessment of alcohol use and qualitative analysis of the 12 group sessions and individual interviews with each participant, analysed using feminist theory	Group work intervention for problematic alcohol use	34 (range 22-44)	Lesbian 12	Female 12	White 12
Senreich (2009)	A comparison of perceptions, reported abstinence, and completion rates of gay, lesbian, bisexual, and heterosexual clients in substance abuse treatment	USA	227	120	Mixed-methods study comparing heterosexual and LGB former clients of substance abuse treatment programs, examining treatment experiences and outcomes, and reasons for leaving treatment	Substance abuse treatment programs (outpatient programs, residential programs, inpatient rehabs, and methadone maintenance programs)	41.6	Bisexual women 9, bisexual men 13, lesbian women 30, gay men 68	Men 81, women 39	Not reported specifically for SM, of all participants: White 50%, Black 30%, Hispanic 19%, Other 1%
Smiles (2023)	Needle exchange practitioners accounts of delivering harm reduction advice for chemsex: implications for policy and practice	UK	17	Not reported (study of service providers)	Qualitative realist study using interviews with 17 harm reduction practitioners working at needle exchanges, focusing on their understandings of chemsex and its treatment, and analysed using thematic analysis	One integrated substance misuse service with a needle exchange program, one stand-alone harm-reduction service	Not reported (study of service providers)	Not reported (study of service providers)	Male 9, female 8	Not reported (study of service providers)
Sucaldito (2023)	Exploring Individual and Contextual Factors Associated With Sexual Risk and Substance Use Among Underserved GBQMSM and Transgender and	USA	16	16	Qualitative study using interviews with 16 gay, bisexual, queer, and other men who have sex with men in rural Appalachia about risks for HIV, STI and HCV transmission and access to care, analysed	Drug harm reduction programs, including syringe exchange services	33.3 (range 24-50)	Gay/homosexual 6, queer 5, heterosexual MSM 2, pansexual 1, bisexual 1, queer/bisexual/pansexual 1	Cisgender male 5, nonbinary 4, transgender woman 3, genderfluid nonbinary 1, genderfluid transmasculine	White 9, Latino 2, Black 1, Hispanic and Native American 1, Native American/Eastern Band of Cherokee Indians 1, African American and

	Nonbinary Persons in South Central Appalachia				using grounded theory methods				1, transgender man 1, two-spirit 1	Caucasian 1, Mixed, Puerto Rican and Italian 1
Tan (2018)	Chemsex among gay, bisexual, and other men who have sex with men in Singapore and the challenges ahead: A qualitative study	Singapore	30	30	Qualitative study using interviews with 30 gay, bisexual, and other men involved in chemsex, focusing on sexual risk behaviours and chemsex experiences, analysed using applied thematic analysis methods and a socio-ecological framework	Addiction management services	Not reported, range 18-39	Gay, bisexual, and other men who have sex with men 30	Men 30	Not reported
Tan (2021)	Exploring the role of trauma in underpinning sexualised drug use ('chemsex') among gay, bisexual and other men who have sex with men in Singapore	Singapore	33	33	Qualitative study using interviews with 33 gay, bisexual, and other men who have sex with men with a history of sexualised drug use, focusing on experiences of chemsex, treatment-seeking, trauma, and resilience, analysed using thematic analysis methods	Substance use treatment services	Not reported (most participants age 31 to 40)	Gay, bisexual, and other men who have sex with men 33	Men 33	Chinese 25, Malay 3, Indian 3, Others 2
Tomkins (2018)	How can those engaging in chemsex best be supported? An online survey to gain intelligence in Greater Manchester	UK	52	52	Quantitative study using an online convenience survey of MSM involved in chemsex in Greater Manchester, focusing on their sexual health and perceived barriers and facilitators to accessing chemsex support services	Chemsex support services	Not reported (range 25-64)	Men who have sex with men 52	Men 52	Unknown
Travers (1996)	Barriers to accessibility for lesbian and gay youth needing addictions services	Canada	17	17	Qualitative interviews with lesbian and gay 17 to 24 year-olds with experience of using addiction services, exploring barriers to service accessibility, using feminist sociology and existential-phenomenological psychology approaches and analysed thematically	All specialist addiction services, such as residential treatment, addiction counselling programmes, day treatment programmes and/or detox facilities	Not reported (range 17-24)	Gay men 14, lesbian women 3	Male 14, female 3	Caucasian 12, People of Colour (non-Native Canadians) 2, Native Canadians 2
Underhill (2014)	Access to healthcare, HIV/STI testing, and preferred pre-exposure prophylaxis providers among men who have sex with men and men	USA	94	94	Multi-stage qualitative study using focus groups and interviews with men who have sex with men, some of whom were sex workers, asking about access to healthcare, STI	Drug rehabilitation services	Not reported (range 21-70)	MSM 94, of which 7 identified as 'straight/heterosexual'	Male 94	Race:<br>White 72, African American 18, Native American 2, Asian 1, refused 1

	who engage in street-based sex work in the US				testing and PrEP, analysed thematically					Ethnicity: Hispanic/Latino identity 13
Viera (2022)	Substance Use Treatment Engagement among Men Who Have Sex with Men Who Use Stimulants in the Northeastern United States	USA	21	21	Qualitative study using interviews with 21 stimulant-using men who have sex with men, exploring perceptions of and experiences with substance use treatment, analysed using thematic analysis	Substance use treatment services	39.3	Gay 11, straight MSM 3, bisexual 4, other 3	Men 21	White/Caucasian 12, Black/African American 6, Other 3
Washington (2011)	Exploring the perspectives of substance abusing Black men who have sex with men and women in addiction treatment programs: a need for a human sexuality educational model for addiction professionals	USA	105	105	Qualitative study utilising focus groups of Black male sex workers who use injecting drugs, all of whom identified as men who have sex with both women and men, exploring their perspectives on useful topics for addiction professionals to address, analysed using grounded theory	Substance use/addiction treatment services	31.6	Men who have sex with women and men 105	Men 105	Black 105 (of which African American not of Hispanic origin 94, African American Hispanic origin 11)
Willging (2018)	A Mixed-Method Assessment of a Pilot Peer Advocate Intervention for Rural Gender and Sexual Minorities	USA	47	47 sexual and gender minorities	Mixed-methods assessment of a pilot peer advocate intervention for rural gender and sexual minorities in New Mexico; qualitative semi-structured interviews analysed using grounded theory, and quantitative questionnaires analysed descriptively	Specialist mental health/substance use disorder services	35.1 (range 18-61)	Sexual and gender minorities 47, of which: bisexual 19, lesbian/gay 19, questioning 4, other 3, heterosexual 2	Women/girl 23, man/boy 18, transgender 8<br><br>(Note some participants selected more than one category)	American Indian 21, African American 3, Asian American or Asian 1, Non-Hispanic white 14, Hispanic/Latina/o 14<br><br>(Note some participants selected more than one category)

Supplementary Table 5: MMAT-2018 Responses for Included Studies

Note: Mixed methods studies advise rating questions using quality criteria 5, plus 1 and then either 2, 3, or 4.

**First author and year of publication**	**Screening: S1**	**Screening: S2**	**Type of study, MMAT questions used**	**Quality: Q1**	**Quality: Q2**	**Quality: Q3**	**Quality: Q4**	**Quality: Q5**	**Comments**
Allen (2016)	Yes	Yes	Quantitative non-randomised: 3	Yes	Yes	Yes	Yes	Yes	

Aslan (2024)	Yes	Yes	Quantitative descriptive: 4	Yes	Yes	Yes	No	Yes	Response rate 55.2%; likely non-response bias
Barbara (2002)	Yes	Yes	Qualitative: 1	Yes	Yes	Yes	Yes	Yes	
Batchelder (2021)	Yes	Yes	Quantitative non-randomised: 3	Yes	Yes	Yes	No	Yes	No analysis of potential confounders
Bernier (2024)	Yes	Yes	Mixed-methods: 5	No	Yes	Yes	Yes	No	No explicit rationale for mixed methods approach, however derived qualitative themes subsequently analysed quantitatively<br>Qualitative component clearly described and executed; some problems with quantitative components as below
"			+ Qualitative: 1	Yes	Yes	Yes	Yes	Yes	
"			+ Quantitative non-randomised: 3	Can't tell	Yes	Yes	No	Yes	Small sample of SM people, not representatively selected, no reporting of number of people who were approached but declined to participate (i.e. potential non-response bias)<br>No analysis of potential confounders, however exploratory study with small number of SM participants
Blanchette (2024)	Yes	Yes	Qualitative: 1	Yes	Yes	Yes	Yes	Yes	
Bourne (2015)	Yes	Yes	Qualitative: 1	Yes	Yes	Yes	Yes	Yes	
Braine (2014)	Yes	Yes	Qualitative: 1	Yes	Yes	Yes	Yes	Yes	
Brown (2017)	Yes	Yes	Qualitative: 1	Yes	Yes	Yes	Yes	Yes	
Card (2021)	Yes	Yes	Quantitative non-randomised: 3	No	Yes	Yes	Yes	Yes	46.8% of those consented completed the (convenience) survey, likely non-response bias (though included vs excluded participants broadly similar in demographic characteristics)
Card (2022)	Yes	Yes	Quantitative non-randomised: 3	No	Yes	Yes	No	Yes	36.2% of those consented fully completed the (convenience) survey, likely non-response bias (included vs excluded participants broadly similar in demographic characteristics but differ in sexual orientation and readiness to change)<br>No explicit analysis of potential confounders
Cochran (2007)	Yes	Yes	Quantitative descriptive: 4	Yes	Can't tell	Yes	No	Yes	No information reported about non-responders to survey<br>Response rate 23.4%; high risk of non-response bias
Conner (2023)	Yes	Yes	Quantitative non-randomised: 3	Can't tell	Yes	Yes	No	Yes	Although high completion rate of survey, no information reported about potential size of eligible population for survey; convenience sampling<br>Potential confounders not explicitly addressed in analysis
Copeland (1992)	Yes	Yes	Quantitative non-randomised: 3	Can't tell	Yes	Yes	Yes	Yes	Minimal information reported about recruitment processes and no information on response bias (i.e. how many women were eligible/approached but declined to participate)

Corliss (2006)	Yes	Yes	Quantitative descriptive: 4	Yes	Yes	Yes	Can't tell	Yes	Non-response rate not reported (although a number of recruitment methods used to obtain a more representative sample)
Dyar (2023)	Yes	Yes	Quantitative non-randomised: 3	Yes	Yes	Yes	Yes	Yes	
Flores (2017)	Yes	Yes	Quantitative descriptive: 4	Yes	Yes	Yes	No	Yes	Global convenience sample of MSM recruited via online-based methods - high risk of non-response bias
Forenza (2016)	Yes	Yes	Qualitative: 1	Yes	Yes	Yes	Yes	Yes	
Freestone (2025)	Yes	Yes	Qualitative: 1	Yes	Yes	Yes	Yes	Yes	
Goodyear (2020)	Yes	Yes	Qualitative: 1	Yes	Yes	Yes	Yes	Yes	
Gorman (1997)	Yes	Yes	Qualitative: 1	Yes	Yes	Yes	No	Can't tell	Minimal analysis and contextualisation within existing theory, as preliminary report/exploratory study
Graf (2018)	Yes	Yes	Qualitative: 1	Yes	Yes	Yes	No	No	No quotes provided to justify the themes in final results section, 'support system's response to chemsex' Although this is an 'explorative' paper, does not coherently draw on collected data throughout results and discussion sections
Green (2011)	Yes	Yes	Quantitative non-randomised: 3	No	Yes	Yes	No	Yes	29.9% of those who initiated the survey answered one or more questions – high risk of non-response bias Potential confounders not explicitly addressed in analysis
Hall (1994)	Yes	Yes	Qualitative: 1	Yes	Yes	Yes	Yes	Yes	
Haney (2021)	Yes	Yes	Quantitative non-randomised: 3	Yes	Yes	Yes	Yes	Yes	
Hellman (1989)	Yes	Yes	Quantitative descriptive: 4	Yes	Can't tell	Yes	No	Yes	Unable to judge whether respondents differed from target population of service providers 41% response rate, likely non-response bias
Hodges (2023)	Yes	Yes	Quantitative non-randomised: 3	Yes	Yes	Yes	Yes	Yes	
Hsiang (2018)	Yes	Yes	Qualitative: 1	Yes	Yes	Yes	Yes	Yes	
Hussen (2021)	Yes	Yes	Qualitative: 1	Yes	Yes	Yes	Yes	Yes	
Israelstam (1988)	Yes	Yes	Quantitative descriptive: 4	Yes	Yes	Yes	Yes	Yes	
Jeong (2016)	Yes	Yes	Quantitative non-randomised: 3	Yes	Yes	Yes	Yes	Yes	
Joyce (2018)	Yes	Yes	Qualitative: 1	Yes	Yes	Yes	Yes	Yes	
Kanouse (2005)	Yes	Yes	Quantitative non-randomised: 3	Can't tell	Yes	Yes	No	Yes	Small to medium-size convenience sample of hard-to reach population, likely non-response bias Potential confounders not explicitly addressed in analysis
Krasnova (2021)	Yes	Yes	Quantitative non-randomised: 3	Yes	Yes	Yes	Yes	Yes	
Matthews (2006)	Yes	Yes	Qualitative: 1	Yes	No	Yes	No	Yes	Small sample size of 10 participants Mostly short quotations used to illustrate themes, some themes not substantiated by quotations

Mays (1994)	Yes	Yes	Quantitative descriptive: 4	Yes	Can't tell	Yes	No	Yes	Small to medium size convenience sample, unable to ascertain how representative of target population<br>Unable to ascertain number of eligible women who declined to participate; high risk of non-response bias
McGaughey (2023)	Yes	Yes	Qualitative: 1	Yes	No	Yes	No	Yes	Small sample size of 10 participants<br>Mostly short quotations used to illustrate themes, some themes not substantiated by quotations, however note pilot study
Mericle (2018)	Yes	Yes	Qualitative: 1	Yes	Can't tell	Yes	Yes	Yes	Small sample size of 11 participants, however all elite interviews
Micale (2024)	Yes	Yes	Quantitative descriptive: 4	Yes	Yes	Yes	Yes	Yes	
Nieder (2025)	Yes	Yes	Qualitative: 1	Yes	Yes	Yes	Yes	Yes	
Parry (2008)	Yes	Yes	Qualitative: 1	Yes	Yes	Yes	Yes	Yes	
Paschen-Wolff (2024)	Yes	Yes	Qualitative: 1	Yes	Yes	Yes	Yes	Yes	
Penn (2013)	Yes	Yes	Qualitative: 1	Yes	No	Yes	No	Yes	Small sample size of 10 participants<br>Mostly short quotations used to illustrate themes, some themes not substantiated by quotations
Pennay (2018)	Yes	Yes	Qualitative: 1	Yes	Yes	Yes	Yes	Yes	
Ralphs (2018)	Yes	Yes	Qualitative: 1	Yes	Yes	Can't tell	Yes	Can't tell	Qualitative data analysis method not explicitly described or stated (appears to be thematic analysis)
Ramakrishnan (2023)	Yes	Yes	Quantitative non-randomised: 3	Yes	Yes	Yes	Yes	Yes	
Rice (2024)	Yes	Yes	Quantitative non-randomised: 3	Yes	Yes	Yes	Yes	Yes	
Roth (2021)	Yes	Yes	Quantitative non-randomised: 3	Yes	Yes	Yes	Yes	Yes	
Rowan (2013)	Yes	Yes	Qualitative: 1	Yes	Yes	Yes	Yes	Yes	
Rowan (2022)	Yes	Yes	Quantitative non-randomised: 3	Yes	Yes	Yes	Yes	Yes	
Saulnier (1997)	Yes	Yes	Mixed-methods: 5	Can't tell	Yes	Yes	Yes	No	Some rationale for including qualitative and quantitative components, however not fully elaborated<br>Quality issues with component studies
"			+ Qualitative: 1	Yes	Can't tell	Can't tell	Yes	Yes	Small to medium sample size of 12 participants<br>Qualitative analysis method described but not cited, and rationale for choosing that method not provided
"			+ Quantitative descriptive: 4	No	No	Yes	No	Yes	Small to medium size convenience sample unlikely to be representative of target population<br>Inclusion and exclusion criteria not clear<br>High risk of non-response bias
Senreich (2009)	Yes	Yes	Mixed-methods: 5	Can't tell	Yes	Yes	Yes	No	Although mixed-methods design appropriate for research question, no explicit rationale provided in methods<br>Quality issues with component studies

"			+ Qualitative: 1	Yes	No	No	Yes	No	Small qualitative component to larger study, not seeking in-depth qualitative data<br>Data analysis method not described or cited<br>Limited coherence due to above, limited interpretation and integration of qualitative findings
"			+ Quantitative non-randomised: 3	Yes	Yes	Yes	Yes	Yes	
Smiles (2023)	Yes	Yes	Qualitative: 1	Yes	Yes	Yes	Yes	Yes	
Sucaldito (2023)	Yes	Yes	Qualitative: 1	Yes	Yes	Yes	Yes	Yes	
Tan (2018)	Yes	Yes	Qualitative: 1	Yes	Yes	Yes	Yes	Yes	
Tan (2021)	Yes	Yes	Qualitative: 1	Yes	Yes	Yes	Yes	Yes	
Tomkins (2018)	Yes	Yes	Quantitative descriptive: 4	Yes	Can't tell	Yes	No	Yes	Relatively small convenience sample, unclear how representative of the target population<br>High risk of non-response bias
Travers (1996)	Yes	Yes	Qualitative: 1	Yes	Yes	Can't tell	Yes	Yes	Analysis method described but not explicitly cited
Underhill (2014)	Yes	Yes	Qualitative: 1	Yes	Yes	Yes	Yes	Yes	
Viera (2022)	Yes	Yes	Qualitative: 1	Yes	Yes	Yes	Yes	Yes	
Washington (2011)	Yes	Yes	Qualitative: 1	Yes	Yes	Yes	Yes	Yes	
Willging (2018)	Yes	Yes	Mixed methods: 5	Yes	Yes	Yes	Yes	No	Some issues with quantitative component study
"			+ Qualitative: 1	Yes	Yes	Yes	Yes	Yes	
"			+ Quantitative descriptive: 4	Yes	Can't tell	Yes	No	Yes	Medium sample size but hard to ascertain representativeness, potential number of eligible participants – convenience sampling<br>High risk of non-response bias

Supplementary Table 6: Excluded Studies

**Study Title**	**Full Citation**	**Exclusion Reason**	**Additional Notes**
A community-led, harm-reduction approach to chemsex: case study from Australia's largest gay city	Stardust Z, Kolstee J, Joksic S, Gray J, Hannan S. A community-led, harm-reduction approach to chemsex: case study from Australia's largest gay city. Sex Health. 2018;15(2):179-181.	Not a primary research article	Case study
A model for the treatment of lesbian and gay alcohol abusers	Ratner E. A Model for the Treatment of Lesbian and Gay Alcohol Abusers. Alcoholism Treatment Quarterly. 1988; 5(1–2), 25–46.	Not a primary research article	Editorial/practice paper
Alcohol interventions for LGBTQ+ adults: A systematic review	Dimova ED, Elliott L, Frankis J, Drabble L, Wiencierz S, Emslie C. Alcohol interventions for LGBTQ+ adults: A systematic review. Drug Alcohol Rev. 2022;41(1):43-53.	Not a primary research article	Review
Alcohol Use and Alcohol-Related Problems Among Sexual Minority Women	Hughes T. Alcohol Use and Alcohol-Related Problems among Sexual Minority Women. Alcohol Treat Q. 2011;29(4):403-435.	Not a primary research article	Review

Anova Health Institute's harm reduction initiatives for people who use drugs	Hugo JM, Rebe KB, Tsouroulis E, Manion A, de Swart G, Struthers H, McIntyre JA. Anova Health Institute's harm reduction initiatives for people who use drugs. Sex Health. 2018;15(2):176-178.	Not a primary research article	Case report
Chemical Dependency and Depression in Lesbians and Gay Men: What Helps?	Finnegan DG & McNally EB. Chemical Dependency and Depression in Lesbians and Gay Men: What Helps? Journal of Gay & Lesbian Social Services. 1996;4(2):115–129.	Not a primary research article	Review
Comprehensive clinical care for men who have sex with men: an integrated approach	Mayer KH, Bekker LG, Stall R, Grulich AE, Colfax G, Lama JR. Comprehensive clinical care for men who have sex with men: an integrated approach. Lancet. 2012;380(9839):378-87.	Not a primary research article	Review
Correction to: The relationship between sexual and gender stigma and difficulty accessing primary and mental healthcare services among LGBTQI+ populations in Thailand: Findings from a national survey	Moallef S, Salway T, Phanuphak N, Kivioja K, Pongruengphant S and Hayashi K. Correction to: The Relationship Between Sexual and Gender Stigma and Difficulty Accessing Primary and Mental Healthcare Services Among LGBTQI + Populations in Thailand: Findings from a National Survey. Int J Ment Health Addiction. 2024;22: 799.	Not a primary research article	Correction
Counseling the homosexual alcoholic	Colcher RW. Counseling the homosexual alcoholic. J Homosex. 1982;7(4):43-52.	Not a primary research article	Editorial/practice paper
Culturally (in)competent? Dismantling health care barriers for sexual minority women	Dearing RL, Hequembourg AL. Culturally (in)competent? Dismantling health care barriers for sexual minority women. Soc Work Health Care. 2014;53(8):739-61.	Not a primary research article	Review
Developing and testing a brief alcohol intervention for lesbian, gay, bisexual, transgender, and queer populations	Mirabito LA. Developing and Testing a Brief Alcohol Intervention for Lesbian, Gay, Bisexual, Transgender, and Queer Populations. Dissertations. 2021;1058.	Not a primary research article	Dissertation/thesis
Developing and testing of an interactive internet-based intervention to reduce sexual harm of sexualised drug use ('chemsex') among men who have sex with men in Hong Kong: a study protocol for a randomised controlled trial	Choi EPH, Chau PH, Wong WCW, Kowk JYY, Choi KWY, Chow EPF. Developing and testing of an interactive internet-based intervention to reduce sexual harm of sexualised drug use (‘chemsex’) among men who have sex with men in Hong Kong: a study protocol for a randomised controlled trial. BMC Public Health. 2021;21:713.	Not a primary research article	Protocol only
Drug use, dependence and mental health among gay, lesbian and bisexual people reporting regular methamphetamine use: Sydney Australia	Matheson A, Roxburgh A, Degenhardt L, Howard J, Down I. Drug use, dependence and mental health among gay, lesbian and bisexual people reporting regular methamphetamine use: Sydney Australia. 2019.	Not a primary research article	Report
Dual diagnosis issues with homosexual persons	Hellman RE. Dual Diagnosis Issues with Homosexual Persons. Journal of Chemical Dependency Treatment. 1993;5(1):105–117.	Not a primary research article	Case series
Effective intervention and treatment for lesbians	Drabble L & Underhill BL. Effective intervention and treatment for lesbians. In S.L.A. Straussner & S. Brown (Eds.), The Handbook of Addiction Treatment for Women (pp. 399–422). 2002. Jossey-Bass/Wiley.	Not a primary research article	Book chapter
Effectiveness of a culturally tailored SMS alcohol intervention for same-sex attracted women: protocol for an RCT	Bush R, Brown R, McNair R, Orellana L, Lubman DI, Staiger PK. Effectiveness of a culturally tailored SMS alcohol intervention for same-sex attracted women: protocol for an RCT. BMC Womens Health. 2019;19(1):29.	Not a primary research article	Protocol only
Elevated substance use among lesbian and bisexual women: Possible explanations and intervention implications for an urgent public health concern	Rosario M. Elevated substance use among lesbian and bisexual women: possible explanations and intervention implications for an urgent public health concern. Subst Use Misuse. 2008;43(8-9):1268-70.	Not a primary research article	Review/opinion article
Gay and alcoholic: Epidemiologic and clinical issues	Paul JP, Stall R, Bloomfield KA. Gay and alcoholic: Epidemiologic and clinical issues. Alcohol Health & Research World. 1991;15(2):151–160.	Not a primary research article	Review

Impact of perceived public stigma on mental health treatment utilization: differences amongst various demographics	Patel T. Impact of Perceived Public Stigma on Mental Health Treatment Utilization: Differences Amongst Various Demographics. The Chicago School of Professional Psychology ProQuest Dissertations & Theses, 2023. 29993575.	Not a primary research article	Dissertation/thesis
Implementing group therapy for adults with substance use disorders: What research-based evidence?	Lo Coco G, Graffeo MT & Albano G. Implementing group therapy for adults with substance use disorders: What research-based evidence? Group Dynamics: Theory, Research, and Practice. 2024;28(3):199–215.	Not a primary research article	Review
Increasing Cultural Awareness and Sensitivity: Effective Substance Treatment in the Adult Lesbian Population	Taliaferro JD, Lutz B, Moore AK & Scipien K. Increasing Cultural Awareness and Sensitivity: Effective Substance Treatment in the Adult Lesbian Population. Journal of Human Behavior in the Social Environment. 2014;24(5):582–588.	Not a primary research article	Review article
Integrating SMART Recovery and Mental Health Services to Meet the Needs and Goals of LGBTQ Individuals Experiencing Substance Use–Related Problems	McGeough BL, Greenwood EM, Cohen NL & Wootton AR. Integrating SMART Recovery and Mental Health Services to Meet the Needs and Goals of LGBTQ Individuals Experiencing Substance Use–Related Problems. Families in Society. 2022;104(2): 222-233.	Not a primary research article	Editorial
Interventions, Barriers, and Facilitators to Address the Sexual Problems of Gay, Bisexual and Other Men Who Have Sex with Men Living with HIV: A Rapid Scoping Review	Avallone F, Engler K, Cox J, Hickson F, Lebouché B. Interventions, Barriers, and Facilitators to Address the Sexual Problems of Gay, Bisexual and Other Men Who Have Sex with Men Living with HIV: A Rapid Scoping Review. AIDS Behav. 2024;28(2):450-472.	Not a primary research article	Scoping review
Issues and standards in counseling lesbians and gay men with substance abuse concerns	Cheng Z. Issues and Standards in Counseling Lesbians and Gay Men with Substance Abuse Concerns. Journal of Mental Health Counseling. 2003;25(4):323–336.	Not a primary research article	Review
Lesbian alcohol and substance abuse	Mosbacher D. Lesbian Alcohol and Substance Abuse. Psychiatric Annals. 1988;18(1):47-50.	Not a primary research article	Commentary
Meeting the Needs of the Gay and Lesbian Community: Outcomes in the Human Services	Maccio EM & Doueck HJ. Meeting the Needs of the Gay and Lesbian Community: Outcomes in the Human Services. Journal of Gay & Lesbian Social Services. 2002;14(4): 55–73.	Not a primary research article	Review
Men who have sex with men: Health risks, methamphetamine use, and approaches to preventive care	Espejo JB. Men who have sex with men: Health risks, methamphetamine use, and approaches to preventive care. Journal of Men's Health. 2013;10(1):8–13.	Not a primary research article	Case study
Mental health disparities in sexual minority and transgender women: Implications and considerations for treatment	Matouk KM, Schulman JK, Case JAC. Mental Health Disparities in Sexual Minority and Transgender Women: Implications and Considerations for Treatment. Psychiatr Clin North Am. 2023;46(3):583-595.	Not a primary research article	Editorial/review
Methamphetamine abuse: Issues for special populations	Freese TE, Obert J, Dickow A, Cohen J, Lord RH. Methamphetamine abuse: issues for special populations. J Psychoactive Drugs. 2000;32(2):177-82.	Not a primary research article	Case series/review
Mobile-Enhanced Prevention Support Study for Men Who Have Sex With Men and Transgender Women Leaving Jail: Protocol for a Randomized Controlled Trial	Edwards GG, Reback CJ, Cunningham WE, Hilliard CL, McWells C, Mukherjee S, Weiss RE, Harawa NT. Mobile-Enhanced Prevention Support Study for Men Who Have Sex With Men and Transgender Women Leaving Jail: Protocol for a Randomized Controlled Trial. JMIR Res Protoc. 2020;9(9):e18106.	Not a primary research article	Protocol only
Online intervention as strategy to reach men who have sex with other men and who use substances in a sexual context. Development of the MONBUZZ.ca project	Flores-Aranda J, Goyette M, Larose-Osterrath C. Online Intervention as Strategy to Reach Men Who Have Sex With Other Men and Who Use Substances in a Sexual Context. Development of the MONBUZZ.ca Project. Front Psychiatry. 2019;10:183.	Not a primary research article	Literature review and service evaluation

Optimizing Contingency Management With Methamphetamine-Using Men Who Have Sex With Men	Gómez W, Olem D, Andrews R, Discepola MV, Ambrose P, Dilworth SE, Carrico AW. Optimizing Contingency Management with Methamphetamine-Using Men who Have Sex with Men. Cogn Behav Pract. 2018;25(2):286-295.	Not a primary research article	Program evaluation
Providing care for lesbian, gay, bisexual, and transgender immigrants at health centers and clinics	Keuroghlian AS, McDowell MJ, Stern TA. Providing Care for Lesbian, Gay, Bisexual, and Transgender Immigrants at Health Centers and Clinics. Psychosomatics. 2018;59(2):193-198.	Not a primary research article	Case report
Providing Unique Support for Health Study Among Young Black and Latinx Men Who Have Sex With Men and Young Black and Latinx Transgender Women Living in 3 Urban Cities in the United States: Protocol for a Coach-Based Mobile-Enhanced Randomized Control Trial	Arrington-Sanders R, Hailey-Fair K, Wirtz A, Cos T, Galai N, Brooks D, Castillo M, Dowshen N, Trexler C, D'Angelo LJ, Kwait J, Beyrer C, Morgan A, Celentano D; PUSH Study. Providing Unique Support for Health Study Among Young Black and Latinx Men Who Have Sex With Men and Young Black and Latinx Transgender Women Living in 3 Urban Cities in the United States: Protocol for a Coach-Based Mobile-Enhanced Randomized Control Trial. JMIR Res Protoc. 2020;9(9):e17269.	Not a primary research article	Protocol only
Queering recovery: A proposed model for LGBTQ+-affirmative relapse prevention	Chaney MP, Mason N. Queering recovery: A proposed model for LGBTQ+-affirmative relapse prevention. Journal of Addictions and Offender Counseling. 2024;45(1):98-110.	Not a primary research article	Editorial/comment
Service Engagement with High-Risk Men Who Have Sex with Men: Challenges and Implications for Social Work Practice	Natale AP, Moxley DP. Service engagement with high-risk men who have sex with men: challenges and implications for social work practice. Soc Work Health Care. 2009;48(1):38-56.	Not a primary research article	Review
Sex like you can't even imagine: "Crystal," crack and gay men	Guss JR. Sex Like You Can't Even Imagine: "Crystal," Crack and Gay Men. Journal of Gay & Lesbian Psychotherapy. 2000;3(3–4):105–122.	Not a primary research article	Comment/clinical practice article
Sexualised drug use among LGBT people: A mixed methods study of reasons for engagement and associations with physical and psychological wellbeing	Hibbert M. Sexualised drug use among LGBT people: a mixed methods study of reasons for engagement and associations with physical and psychological wellbeing. Doctoral thesis, Liverpool John Moores University. 2020.	Not a primary research article	Thesis/dissertation
Specific approaches and techniques in the treatment of gay male alcohol abusers	Smith TM. Specific Approaches and Techniques in the Treatment of Gay Male Alcohol Abusers. Journal of Homosexuality. 1982;7(4):53–69.	Not a primary research article	Review
Substance Abuse and Dependency in Gay Men and Lesbians	Anderson SC. Substance Abuse and Dependency in Gay Men and Lesbians. Journal of Gay & Lesbian Social Services. 1996;5(1):59–76.	Not a primary research article	Review
Substance Abuse Treatment as HIV Prevention for Men Who Have Sex with Men	Shoptaw S, Frosch D. Substance Abuse Treatment as HIV Prevention for Men Who Have Sex with Men. AIDS Behav. 2000;4:193–203.	Not a primary research article	Review
Substance abuse, internalized homophobia, and gay men and lesbians: Psychodynamic issues and clinical implications	Cabaj RP. Substance abuse, internalized homophobia, and gay men and lesbians: Psychodynamic issues and clinical implications. In J.R. Guss & J. Drescher (Eds.), Addictions in the Gay and Lesbian Community. (pp. 5–24). 2000. Haworth Press.	Not a primary research article	Book chapter
Substance Use in Lesbian, Gay, and Bisexual Populations: An Update on Empirical Research and Implications for Treatment	Green KE, Feinstein BA. Substance use in lesbian, gay, and bisexual populations: an update on empirical research and implications for treatment. Psychol Addict Behav. 2012;26(2):265-78.	Not a primary research article	Review
The check-up: In-person, computerized, and telephone adaptations of motivational enhancement treatment to elicit voluntary participation by the contemplator	Walker DD, Roffman RA, Picciano JF, Stephens RS. The check-up: in-person, computerized, and telephone adaptations of motivational enhancement treatment to elicit voluntary participation by the contemplator. Subst Abuse Treat Prev Policy. 2007;2:2.	Not a primary research article	Review/clinical practice paper

The experiences of LGBQ clients accessing substance use treatment: Understanding barriers and facilitators through minority stress theory	Spalding A. The experiences of LGBQ clients accessing substance use treatment: Understanding barriers and facilitators through minority stress theory. Alliant International University ProQuest Dissertations & Theses, 2023.	Not a primary research article	Thesis/dissertation
The impacts of internalized homonegativity, shame and self-compassion on quality of recovery in gay, bisexual and queer males in addiction recovery	Campagna DL. The impacts of internalized homonegativity, shame and self-compassion on quality of recovery in gay, bisexual and queer males in addiction recovery. Alliant International University ProQuest Dissertations & Theses, 2022.	Not a primary research article	Thesis/dissertation
Treating gay, lesbian, bisexual and transgender professionals with addictive disease	Ziegler PP. Treating Gay, Lesbian, Bisexual and Transgender Professionals with Addictive Disease. Journal of Gay & Lesbian Psychotherapy. 2000;3(3–4):59–68.	Not a primary research article	Review/clinical practice paper
Understanding the experiences of gay men in addiction treatment: a phenomenological study	Cullen J. Understanding the experiences of gay men in addiction treatment: a phenomenological study. Thesis (Ph.D.)-University of Toronto, 2004.	Not a primary research article	Thesis/dissertation
Understanding the Impact of Childhood Sexual Abuse on Men's Risk Behavior: Protocol for a Mixed-Methods Study	Downing MJ Jr, Brown D, Steen J, Benoit E. Understanding the Impact of Childhood Sexual Abuse on Men's Risk Behavior: Protocol for a Mixed-Methods Study. JMIR Res Protoc. 2018;7(2):e62.	Not a primary research article	Protocol only
What really worked? A case analysis and discussion of confrontational intervention for substance abuse in marginalized women	Hall JM. What really worked? A case analysis and discussion of confrontational intervention for substance abuse in marginalized women. Arch Psychiatr Nurs. 1993;7(6):322-7.	Not a primary research article	Case study
Who should be doing what about the gay alcoholic?	Zigrang TA. Who Should Be Doing What about the Gay Alcoholic? Journal of Homosexuality. 1982;7(4):27–35.	Not a primary research article	Review/commentary
Staying Negative -- It's Not Automatic: A Harm-Reduction Approach to Substance Use and Sex	Elovich R. Staying negative--it's not automatic: a harm-reduction approach to substance use and sex. AIDS Public Policy J. 1996;11(2):66-77.	Not in adults	
Failure to get into substance abuse treatment	Fisher DG, Reynolds GL, D'Anna LH, Hosmer DW, Hardan-Khalil K. Failure to get into substance abuse treatment. J Subst Abuse Treat. 2017;73:55-62.	Not about sexual minority people	Data aggregated between heterosexual and SM people for the barriers to access, so unable to draw conclusions
Structural determinants of tailored behavioral health services for sexual and gender minorities in the United States, 2010 to 2020: a panel analysis	Cascalheira CJ, Helminen EC, Shaw TJ, Scheer JR. Structural determinants of tailored behavioral health services for sexual and gender minorities in the United States, 2010 to 2020: a panel analysis. BMC Public Health. 2022;22(1):1908.	Not about sexual minority people	
"What if I get sick, where shall I go?": a qualitative investigation of healthcare engagement among young gay and bisexual men in Nairobi, Kenya	Mwaniki SW, Kaberia PM, Mugo PM, Palanee-Phillips T. "What if I get sick, where shall I go?": a qualitative investigation of healthcare engagement among young gay and bisexual men in Nairobi, Kenya. BMC Public Health. 2024;24(1):52.	Not about specialist addiction services	
'It kills the freedom or the spirit of people being who they are': impact of sexuality-based stigma and discrimination on the lives of gay and bisexual men in Kenya	Jauregui JC, Lewis KA, Moore DM, Ogunbajo A, Odero WW, Wambaya J, Onyango DP, Jadwin-Cakmak L, Harper GW. ‘It kills the freedom or the spirit of people being who they are’: impact of sexuality-based stigma and discrimination on the lives of gay and bisexual men in Kenya. Global Public Health. 2025;20(1).	Not about specialist addiction services	
A qualitative study to explore the healthcare-seeking experiences of men who have sex with men (MSM) and transgender women (TGW) in Rwanda	Isano S, Yohannes T, Igihozo G, Ndatinya GI & Wong R. A qualitative study to explore the healthcare-seeking experiences of men who have sex with men (MSM) and transgender women (TGW) in Rwanda. BMC Health Serv Res. 2023;23:291.	Not about specialist addiction services	
Alcohol and tobacco consumption among Australian sexual minority women: Patterns of use and service engagement	Amos N, Bourne A, Hill AO, Power J, McNair R, Mooney-Somers J, Pennay A, Carman M, Lyons A. Alcohol and tobacco consumption among Australian sexual minority women: Patterns of use and service engagement. Int J Drug Policy. 2022;100:103516.	Not about specialist addiction services	Not entirely clear that discussing specialist addictions services - 'professional support for alcohol use'

Alcohol use, behavioral and mental health help-seeking, and treatment satisfaction among sexual minority women	Scheer JR, Batchelder AW, Bochicchio LA, Kidd JD, Hughes TL. Alcohol use, behavioral and mental health help-seeking, and treatment satisfaction among sexual minority women. Alcohol Clin Exp Res. 2022;46(4):641-656.	Not about specialist addiction services	
Assessing the feasibility of harm reduction services for MSM: The late night breakfast buffet study	Rose VJ, Raymond HF, Kellogg TA, McFarland W. Assessing the feasibility of harm reduction services for MSM: the late night breakfast buffet study. Harm Reduct J. 2006;3:29.	Not about specialist addiction services	
At the intersection of marginalised identities: lesbian, gay, bisexual and transgender people's experiences of injecting drug use and hepatitis C seroconversion	Deacon RM, Mooney-Somers J, Treloar C, Maher L. At the intersection of marginalised identities: lesbian, gay, bisexual and transgender people's experiences of injecting drug use and hepatitis C seroconversion. Health Soc Care Community. 2013;21(4):402-10.	Not about specialist addiction services	
Barriers and facilitators to medication-assisted treatment for cocaine use disorder among men who have sex with men: a qualitative study	Hsiang E, Patel K, Wilson EC, Dunham A, Ikeda J, Matheson T, Santos GM. Barriers and facilitators to medication-assisted treatment for cocaine use disorder among men who have sex with men: a qualitative study. Addict Sci Clin Pract. 2024;19(1):84.	Not about specialist addiction services	
Barriers and unmet need for supportive services for HIV patients in care in Los Angeles County, California.	Wohl AR, Carlos JA, Tejero J, Dierst-Davies R, Daar ES, Khanlou H, Cadden J, Towner W, Frye D. Barriers and unmet need for supportive services for HIV patients in care in Los Angeles County, California. AIDS Patient Care STDS. 2011;25(9):525-32.	Not about specialist addiction services	
Changes in alcohol, tobacco, cannabis, and other substance use and its association with mental health during the COVID-19 pandemic among sexual minority men in Eastern European and Central Asian countries	Hong C, Mammadli T, Lunchenkov N, Garner A, Howell S, Holloway IW. Changes in alcohol, tobacco, cannabis, and other substance use and its association with mental health during the COVID-19 pandemic among sexual minority men in Eastern European and Central Asian countries. J Affect Disord. 2024;359:302-307.	Not about specialist addiction services	
Chems4EU: chemsex use and its impacts across four European countries in HIV-positive men who have sex with men attending HIV services	Whitlock GG, Protopapas K, Bernardino JI, Imaz A, Curran A, Stingone C, Shivasankar S, Edwards S, Herbert S, Thomas K, Mican R, Prieto P, Nestor Garcia J, Andreoni M, Hill S, Okhai H, Stuart D, Bourne A, Conway K. Chems4EU: chemsex use and its impacts across four European countries in HIV-positive men who have sex with men attending HIV services. HIV Med. 2021;22(10):944-957.	Not about specialist addiction services	More relevant to sexual health clinic support (e.g. 56 Dean St, 'professional chemsex support')
Chemsex and Psychosexual Health in a Large Italian Sample of Men Who Have Sex with Men (MSM)	Pessina R, Pavanello Decaro S, Torri C, Prunas A. Chemsex and Psychosexual Health in a Large Italian Sample of Men Who Have Sex with Men (MSM). Sexuality & Culture. 2025;10.1007/s12119-025-10339-y.	Not about specialist addiction services	Reports on generic 'professional support for chemsex' but no participants had attended specific addiction services
Comparing substance use and mental health among sexual and gender minority and heterosexual cisgender youth experiencing homelessness	Hao J, Beld M, Khoddam-Khorasani L, Flentje A, Kersey E, Mousseau H, Frank J, Leonard A, Kevany S, Dawson-Rose C. Comparing substance use and mental health among sexual and gender minority and heterosexual cisgender youth experiencing homelessness. PLoS One. 2021;16(3):e0248077.	Not about specialist addiction services	
Correlates of shared methamphetamine injection among methamphetamine-injecting treatment seekers: the first report from Iran	Mehrjerdi ZA, Abarashi Z, Noroozi A, Arshad L, Zarghami M. Correlates of shared methamphetamine injection among methamphetamine-injecting treatment seekers: the first report from Iran. Int J STD AIDS. 2014;25(6):420-7.	Not about specialist addiction services	
Demand for and availability of specialist chemsex services in the UK: A cross-sectional survey of sexual health clinics	Wiggins H, Ogaz D, Mebrahtu H, Sullivan A, Bowden-Jones O, Field N, Hughes G. Demand for and availability of specialist chemsex services in the UK: A cross-sectional survey of sexual health clinics. Int J Drug Policy. 2018;55:155-158.	Not about specialist addiction services	
Describing the effect of COVID-19 on sexual and healthcare-seeking behaviours of men who have sex with men in three counties in Kenya: a cross-sectional study	Shaw SY, Biegun JCS, Leung S, Isac S, Musyoki HK, Mugambi M, Kioko J, Musimbi J, Olango K, Kuria S, Ongaro MK, Walimbwa J, Emmanuel F, Blanchard J, Pickles M, Mishra S, Becker ML, Lazarus L, Lorway R, Bhattacharjee P. Describing the effect of COVID-19	Not about specialist addiction services	

	on sexual and healthcare-seeking behaviours of men who have sex with men in three counties in Kenya: a cross-sectional study. Sex Transm Infect. 2024;100(8):497-503.		
Development of a Small-Group Intervention for Stimulant-Using Men Who Have Sex With Men	Lyons T, Tilmon S & Fontaine YM. Development of a Small-Group Intervention for Stimulant-Using Men Who Have Sex With Men. Journal of Groups in Addiction & Recovery. 2014;9(1):54–70.	Not about specialist addiction services	
Differences in healthcare access, use, and experiences within a community sample of racially diverse lesbian, gay, bisexual, transgender, and questioning emerging adults	Macapagal K, Bhatia R, Greene GJ. Differences in Healthcare Access, Use, and Experiences Within a Community Sample of Racially Diverse Lesbian, Gay, Bisexual, Transgender, and Questioning Emerging Adults. LGBT Health. 2016;3(6):434-442.	Not about specialist addiction services	
Emergency Healthcare Utilization and Unmet Care Needs in Chemsex Users: A Cross-Sectional Survey among Sexual Minority Men	Gonzalez-Recio P, Moreno-García S, Donat M, Palma D, Guerras JM, Belza MJ. Emergency Healthcare Utilization and Unmet Care Needs in Chemsex Users: A Cross-Sectional Survey among Sexual Minority Men. J Community Health. 2025;10.1007/s10900-024-01440-8.	Not about specialist addiction services	
Enablers and inhibitors to the utilization of healthcare services by members of the LGBTQ plus community in Accra, Ghana	Anuga D, Iddrisu M, Konlan K. Enablers and inhibitors to the utilization of healthcare services by members of the LGBTQ+ community in Accra, Ghana. Discover Public Health. 2025;22(1):10.1186/s12982-025-00556-z.	Not about specialist addiction services	
Estimates of alcohol use and clinical treatment needs among homosexually active men and women in the U.S. population	Cochran SD, Keenan C, Schober C, Mays VM. Estimates of alcohol use and clinical treatment needs among homosexually active men and women in the U.S. population. J Consult Clin Psychol. 2000;68(6):1062-71.	Not about specialist addiction services	
Experiences of stigma and subsequent reduced access to health care among women who inject drugs	Brener L, Cama E, Broady T, Harrod ME, Holly C, Caruana T, Beadman K, Treloar C. Experiences of stigma and subsequent reduced access to health care among women who inject drugs. Drug Alcohol Rev. 2024;43(5):1071-1079.	Not about specialist addiction services	
Exploring Anabolic Androgenic Steroid Use Among Cisgender Gay, Bisexual, and Queer Men	Kutscher E, Arshed A, Greene RE, Kladney M. Exploring Anabolic Androgenic Steroid Use Among Cisgender Gay, Bisexual, and Queer Men. JAMA Netw Open. 2024;7(5):e2411088.	Not about specialist addiction services	
Factors related to counselor preference among gays and lesbians	McDermott D, Tyndall L, Lichtenberg JW. Factors related to counselor preference among gays and lesbians. Journal of Counseling & Development. 1989;68(1):31–35.	Not about specialist addiction services	
Feasibility, acceptability, and perceived usefulness of a community-evidence-based harm reduction intervention for sexualized stimulant use among Mexican gay, bisexual, and other men who have sex with men	Rafful C, Orozco R, Peralta D, Jiménez-Rivagorza L, Medina-Mora ME, Gutiérrez N, Morales-Gutierrez M. Feasibility, acceptability, and perceived usefulness of a community-evidence-based harm reduction intervention for sexualized stimulant use among Mexican gay, bisexual, and other men who have sex with men. Harm Reduct J. 2024;21(1):95.	Not about specialist addiction services	
Gay, bisexual, and other men who have sex with men: Barriers and facilitators to healthcare access in Ottawa	Haines M, O'Byrne P, & MacPherson P. Gay, bisexual, and other men who have sex with men: Barriers and facilitators to healthcare access in Ottawa. The Canadian Journal of Human Sexuality. 2021;30(3):339-348.	Not about specialist addiction services	
Health care access and health behaviors among men who have sex with men: The cost of health disparities	McKirnan DJ, Du Bois SN, Alvy LM, Jones K. Health care access and health behaviors among men who have sex with men: the cost of health disparities. Health Educ Behav. 2013;40(1):32-41.	Not about specialist addiction services	
Health disparities in access to health care for HIV infection, substance abuse, and mental health among	Loza O, Provencio-Vasquez E, Mancera B, & De Santis J. Health disparities in access to health care for HIV infection, substance abuse, and mental health among Latino men	Not about specialist addiction services	

Latino men who have sex with men in a U.S.–Mexico Border City	who have sex with men in a U.S.–Mexico Border City. Journal of Gay & Lesbian Social Services. 2021;33(3):320–336.		
Health status, health behaviours and healthcare access of lesbian, gay, bisexual and transgender populations in Turkey	Ercan Sahin N, Aslan F, Emiroglu ON. Health status, health behaviours and healthcare access of lesbian, gay, bisexual and transgender populations in Turkey. Scand J Caring Sci. 2020;34(1):239-246.	Not about specialist addiction services	
Impacts of COVID-19 on sexual risk behaviors, safe injection practices, and access to HIV services among key populations in Zambia: Findings from a rapid qualitative formative assessment	Parmley LE, Nkumbula T, Chilukutu L, Chelu L, Mulemfwe C, Hanunka B, Mwale J, Neal J, Handema R, Kasonde P, Mutale K, Sakala HM, Lahuerta M. Impacts of COVID-19 on sexual risk behaviors, safe injection practices, and access to HIV services among key populations in Zambia: Findings from a rapid qualitative formative assessment. PLoS One. 2023;18(8):e0289007.	Not about specialist addiction services	
Information-seeking behaviours in Australian sexual minority men engaged in chemsex	Demant D, Carroll JA, Saliba B, Bourne A. Information-seeking behaviours in Australian sexual minority men engaged in chemsex. Addict Behav Rep. 2021;16:100399.	Not about specialist addiction services	
Issues in psychotherapy with lesbians and gay men: A survey of psychologists	Garnets L, Hancock KA, Cochran SD, Goodchilds J, Peplau LA. Issues in psychotherapy with lesbians and gay men. A survey of psychologists. Am Psychol. 1991;46(9):964-72.	Not about specialist addiction services	
Issues in the treatment of lesbian and gay men with chronic mental illness	Hellman RE. Issues in the treatment of lesbian women and gay men with chronic mental illness. Psychiatr Serv. 1996;47(10):1093-8.	Not about specialist addiction services	
Lesbian uses of and satisfaction with mental health services: results from Boston Lesbian Health Project	Sorensen L, Roberts SJ. Lesbian uses of and satisfaction with mental health services: results from Boston Lesbian Health Project. J Homosex. 1997;33(1):35-49.	Not about specialist addiction services	
Lesbian, Gay, Bisexual, Sexual-Orientation Questioning Adolescents Seeking Mental Health Services: Risk Factors, Worries, and Desire to Talk About Them	Cniro D, Surko M, Bhandarkar K, Helfgott N, Peake K & Epstein I. Lesbian, Gay, Bisexual, Sexual-Orientation Questioning Adolescents Seeking Mental Health Services: Risk Factors, Worries, and Desire to Talk About Them. Social Work in Mental Health. 2005;3(3):213–234.	Not about specialist addiction services	
Lessons on social and health disparities from older lesbians with alcoholism and the role of interventions to promote culturally competent services	Rowan NL & Giunta N. Lessons on social and health disparities from older lesbians with alcoholism and the role of interventions to promote culturally competent services. Journal of Human Behavior in the Social Environment. 2015;26(2):210–216.	Not about specialist addiction services	
Links between Childhood Abuse, Insidious Trauma, and Methamphetamine Use across the Lifespan among Gay, Bisexual, and Other Men Who Have Sex with Men: A Qualitative Analysis	Berlin GW, Fulcher K, Taylor K, Nguyen T, Montiel A, Moore D, Hull M, Lachowsky NJ. Links Between Childhood Abuse, Insidious Trauma, and Methamphetamine Use Across the Lifespan Among Gay, Bisexual, and Other Men Who Have Sex with Men: A Qualitative Analysis. J Homosex. 2023;70(13):3192-3212.	Not about specialist addiction services	Paper describes 'support services' rather than specific addiction services
Mental Health Problems and Use of Services of Lesbians: Results of the Boston Lesbian Health Project II	Roberts SJ, Grindel CG, Patsdaughter CA, Reardon K & Tarmina MS. Mental Health Problems and Use of Services of Lesbians: Results of the Boston Lesbian Health Project II. Journal of Gay & Lesbian Social Services. 2004;17(4):1–16.	Not about specialist addiction services	
Mental Health Service Utilization Among Young Black Gay, Bisexual, and Other Men Who Have Sex with Men in HIV Care: A Retrospective Cohort Study	Hussen SA, Camp DM, Wondmeneh SB, Doraivelu K, Holbrook N, Moore SJ, Colasanti JA, Ali MK, Farber EW. Mental Health Service Utilization Among Young Black Gay, Bisexual, and Other Men Who Have Sex with Men in HIV Care: A Retrospective Cohort Study. AIDS Patient Care STDS. 2021;35(1):9-14.	Not about specialist addiction services	Aggregates findings for all mental health referrals, unable to disaggregate specialist addiction services
Missed opportunities for healthcare providers to discuss HIV preexposure prophylaxis with people who inject drugs	Vincent W, McFarland W. Missed opportunities for healthcare providers to discuss HIV preexposure prophylaxis with people who inject drugs. Int J Drug Policy. 2022;110:103873.	Not about specialist addiction services	

Motivations for opioid and stimulant use among drug using black sexual minority men: A life course perspective	Dangerfield DT, Heidari O, Cooper J, Allen S, Lucas GM. Motivations for opioid and stimulant use among drug using black sexual minority men: A life course perspective. Drug Alcohol Depend. 2020;215:108224.	Not about specialist addiction services	
Multivariate Patterns of Lifetime Adverse Experiences and Substance Use: Implications for Counseling Services for Lesbian and Bisexual Women	Tubman JG, Moore C, Lee J, Shapiro AJ. Multivariate Patterns of Lifetime Adverse Experiences and Substance Use: Implications for Counseling Services for Lesbian and Bisexual Women. Journal of LGBTQ Issues in Counseling. 2024;18(4):343–362.	Not about specialist addiction services	
Nursing and Health Care Preferences Among Opioid and Stimulant Using Black Sexual Minority Men: An Exploratory Study	Dangerfield DT, Cooper J, Heidari O, Allen S, Winder TJA, Lucas GM. Nursing and Health Care Preferences Among Opioid and Stimulant Using Black Sexual Minority Men: An Exploratory Study. J Assoc Nurses AIDS Care. 2021;32(5):e30-e39.	Not about specialist addiction services	
Older, gay men's navigation of mental health and substance use challenges: A qualitative exploration	Handlovsky I, Wonsiak T, Zakher B, Ferlatte O, Kia H, Oliffe JL. Older, gay men's navigation of mental health and substance use challenges: A qualitative exploration. SSM - Qualitative Research in Health. 2024;6:100484.	Not about specialist addiction services	
Perceived unmet substance use and mental health care needs of acute care patients who use drugs: A cross-sectional analysis using the Behavioral Model for Vulnerable Populations	Kosteniuk B, Salvalaggio G, Wild TC, Gelberg L, Hyshka E. Perceived unmet substance use and mental health care needs of acute care patients who use drugs: A cross-sectional analysis using the Behavioral Model for Vulnerable Populations. Drug Alcohol Rev. 2022;41(4):830-840.	Not about specialist addiction services	Unable to disaggregate substance use services and mental health services
Perspectives on Pain, Engagement in HIV Care, and Behavioral Interventions for Chronic Pain Among Older Sexual Minority Men Living with HIV and Chronic Pain: A Qualitative Analysis	McKetchnie SM, Beaugard C, Taylor SW, O'Cleirigh C. Perspectives on Pain, Engagement in HIV Care, and Behavioral Interventions for Chronic Pain Among Older Sexual Minority Men Living with HIV and Chronic Pain: A Qualitative Analysis. Pain Med. 2021;22(3):577-584.	Not about specialist addiction services	
Perspectives on unhealthy alcohol use among men who have sex with men prescribed HIV pre-exposure prophylaxis: A qualitative study	Strong SH, Oldfield BJ, van den Berg JJ, Cole CA, Biegacki E, Ogbuagu O, Virata M, Chan PA, Edelman EJ. Perspectives on unhealthy alcohol use among men who have sex with men prescribed HIV pre-exposure prophylaxis: A qualitative study. Prev Med Rep. 2023;37:102553.	Not about specialist addiction services	Treatment and support to reduce alcohol use conceptualised generally, not clear if talking about specialist addictions services or wider support/therapy/counselling for alcohol problems
Pre-Empting Stigma and Complicating Trauma: Narratives of Gay and Bisexual Men who Inject Drugs in Australia	Schroeder SE, Treloar C, Bourne A, Stoové M, Doyle J, Hellard M, Pedrana A. Pre-Empting Stigma and Complicating Trauma: Narratives of Gay and Bisexual Men who Inject Drugs in Australia. Qual Health Res. 2023;33(8-9):688-700.	Not about specialist addiction services	
Problematic recreational drug use: is there a role for outpatient sexual health clinics in identifying those not already engaged with treatment services?	Lovett C, Yamamoto T, Hunter L, White J, Dargan PI, Wood DM. Problematic recreational drug use: is there a role for outpatient sexual health clinics in identifying those not already engaged with treatment services? Sex Health. 2015;12(6):501-5.	Not about specialist addiction services	
ReACH2Gether: Iterative Development of a Couples-Based Intervention to Reduce Alcohol use Among Sexual Minority Men Living with HIV and Their Partners	Gamarel KE, Durst A, Zelaya DG, van den Berg JJ, Souza T, Johnson MO, Wu E, Monti PM, Kahler CW. ReACH2Gether: Iterative Development of a Couples-Based Intervention to Reduce Alcohol use Among Sexual Minority Men Living with HIV and Their Partners. AIDS Behav. 2024;28(4):1244-1256.	Not about specialist addiction services	
Scaling Up and Out HIV Prevention and Behavioral Health Services to Latino Sexual Minority Men in South Florida: Multi-Level Implementation Barriers, Facilitators, and Strategies	Harkness A, Satyanarayana S, Mayo D, Smith-Alvarez R, Rogers BG, Prado G, Safren SA. Scaling Up and Out HIV Prevention and Behavioral Health Services to Latino Sexual Minority Men in South Florida: Multi-Level Implementation Barriers, Facilitators, and Strategies. AIDS Patient Care STDS. 2021;35(5):167-179.	Not about specialist addiction services	No disaggregation of HIV/PrEP/sexual health/substance services

Service provision and barriers to care for men who have sex with men engaging in chemsex and sexualised drug use in England	Hibbert MP, Germain JS, Brett CE, Van Hout MC, Hope VD, Porcellato LA. Service provision and barriers to care for men who have sex with men engaging in chemsex and sexualised drug use in England. Int J Drug Policy. 2021;92:103090.	Not about specialist addiction services	Focuses on sexual health services rather than specialist addiction services
Sex, drugs and techno - a qualitative study on finding the balance between risk, safety and pleasure among men who have sex with men engaging in recreational and sexualised drug use	Dennermalm N, Scarlett J, Thomsen S, Persson KI, Alvesson HM. Sex, drugs and techno - a qualitative study on finding the balance between risk, safety and pleasure among men who have sex with men engaging in recreational and sexualised drug use. BMC Public Health. 2021;21(1):863.	Not about specialist addiction services	
Sexual, addiction and mental health care needs among men who have sex with men practicing chemsex – a cross-sectional study in the Netherlands	Evers YJ, Hoebe CJPA, Dukers-Muijrers NHTM, Kampman CJG, Kuizenga-Wessel S, Shilue D, Bakker NCM, Schamp SMAA, Van Buel H, Van Der Meijden WCJPM, Van Liere GAFS. Sexual, addiction and mental health care needs among men who have sex with men practicing chemsex - a cross-sectional study in the Netherlands. Prev Med Rep. 2020;18:101074.	Not about specialist addiction services	
Sexual behavior, sexual identity, and substance abuse among low-income bisexual and non-gay-identifying African American men who have sex with men	Harawa NT, Williams JK, Ramamurthi HC, Manago C, Avina S, Jones M. Sexual behavior, sexual identity, and substance abuse among low-income bisexual and non-gay-identifying African American men who have sex with men. Arch Sex Behav. 2008;37(5):748-62.	Not about specialist addiction services	
Sexual risk and sexual healthcare utilization profiles among Black sexual minority men in the U.S. Deep South	Heidari O, Dangerfield DT & Hickson DA. Sexual risk and sexual healthcare utilization profiles among Black sexual minority men in the U.S. Deep South. AIDS Care. 2020;32(12):1602–1609.	Not about specialist addiction services	
Sexualized drug use and specialist service experience among MSM attending urban and rural sexual health clinics in England and Scotland	Kennedy R, Murira J, Foster K, Heinsbroek E, Keane F, Pal N, Chalmers L, Sinka K. Sexualized drug use and specialist service experience among MSM attending urban and rural sexual health clinics in England and Scotland. Int J STD AIDS. 2021;32(14):1338-1346.	Not about specialist addiction services	
Stigma and help-seeking: The interplay of substance use and gender and sexual minority identity	Benz MB, Palm Reed K, Bishop LS. Stigma and help-seeking: The interplay of substance use and gender and sexual minority identity. Addict Behav. 2019;97:63-69.	Not about specialist addiction services	Not able to disaggregate specialist addiction services
Substance use among sexual minorities in the US - Linked to inequalities and unmet need for mental health treatment? Results from the National Survey on Drug Use and Health (NSDUH)	Rosner B, Neicun J, Yang JC, Roman-Urrestarazu A. Substance use among sexual minorities in the US - Linked to inequalities and unmet need for mental health treatment? Results from the National Survey on Drug Use and Health (NSDUH). J Psychiatr Res. 2021;135:107-118.	Not about specialist addiction services	
Support, care and peer support for gay and bi men engaging in chemsex	Nagington M, King S. Support, care and peer support for gay and bi men engaging in chemsex. Health Soc Care Community. 2022;30(6):e6396-e6403.	Not about specialist addiction services	Paper focuses on chemsex support via peers/sexual health clinics/inpatient admissions
The Experience of Chemsex and Accessing Medical Care for Gay, Bisexual and Other MSM in Russia: An Interpretive Phenomenological Analysis	Lunchenkov N, Rinne-Wolf S, Hughes T, Dwyer B. The Experience of Chemsex and Accessing Medical Care for Gay, Bisexual and Other MSM in Russia: An Interpretive Phenomenological Analysis. J Sex Res. 2025;62(3):360-366.	Not about specialist addiction services	
The Impacts of Intersecting Stigmas on Health and Housing Experiences of Queer Women Sex Workers in Vancouver, Canada	Lyons T, Krüsi A, Edgar E, Machat S, Kerr T, Shannon K. The Impacts of Intersecting Stigmas on Health and Housing Experiences of Queer Women Sex Workers in Vancouver, Canada. J Homosex. 2021;68(6):957-972.	Not about specialist addiction services	
The management of methamphetamine use in sexual settings among men who have sex with men in Malaysia	Lim SH, Akbar M, Wickersham JA, Kamarulzaman A, Altice FL. The management of methamphetamine use in sexual settings among men who have sex with men in Malaysia. Int J Drug Policy. 2018;55:256-262.	Not about specialist addiction services	

The Need and Desire for Mental Health and Substance Use-Related Services Among Clients of Publicly Funded Sexually Transmitted Infection Clinics in Vancouver, Canada	Salway T, Ferlatte O, Shoveller J, Purdie A, Grennan T, Tan DHS, Consolacion T, Rich AJ, Dove N, Samji H, Scott K, Blackwell E, Mirau D, Holgerson N, Wong J, Gilbert M. The Need and Desire for Mental Health and Substance Use-Related Services Among Clients of Publicly Funded Sexually Transmitted Infection Clinics in Vancouver, Canada. J Public Health Manag Pract. 2019;25(3):E1-E10.	Not about specialist addiction services	Paper aggregates 'mental health and substance use needs' for participants in sexual health context - not able to disaggregate specialist addiction services
The relationship between sexual and gender stigma and difficulty accessing primary and mental healthcare services among LGBTQI+ populations in Thailand: Findings from a national survey	Moallef S, Salway T, Phanuphak N, Kivioja K, Pongruengphant S & Hayashi K. The relationship between sexual and gender stigma and difficulty accessing primary and mental healthcare services among LGBTQI+ populations in Thailand: Findings from a national survey. Int J Ment Health Addiction. 2022;20:3244–3261.	Not about specialist addiction services	
Use of Primary Care and Emergency Departments for Substance Use Treatment: The Rural and Urban Divide	Albright N, Dyar C, Morgan E. Use of Primary Care and Emergency Departments for Substance Use Treatment: The Rural and Urban Divide. Subst Use Misuse. 2024;59(2):300-305.	Not about specialist addiction services	
Using social support for LGBTQ clients with mental illness to be out of the closet, in treatment, and in the community	Klein E. Using social support for LGBTQ clients with mental illness to be out of the closet, in treatment, and in the community. Journal of Gay & Lesbian Social Services. 2017;29(3):221–232.	Not about specialist addiction services	
Utilization of cessation resources among HIV-positive and HIV-negative men who smoke and who have sex with men in Chengdu, China	Berg CJ, Nehl EJ, Wang X, Ding Y, He N, Wong FY. Utilization of cessation resources among HIV-positive and HIV-negative men who smoke and who have sex with men in Chengdu, China. Nicotine Tob Res. 2014;16(10):1283-8.	Not about specialist addiction services	
Utilization of mental health services in relation to the intention to reduce chemsex behavior among clients from an integrated sexual health services center in Taiwan	Hung YR, Chuang TT, Chen TW, Chung AC, Wu MT, Hsu ST, Ko NY, Strong C. Utilization of mental health services in relation to the intention to reduce chemsex behavior among clients from an integrated sexual health services center in Taiwan. Harm Reduct J. 2023;20(1):52.	Not about specialist addiction services	Paper focuses on factors associated with joining chemsex group at specialist chemsex centre rather than specialist addiction services
A comparison of predictors of treatment drop-out of women seeking drug and alcohol treatment in a specialist women's and two traditional mixed-sex treatment services	Copeland J, Hall W. A comparison of predictors of treatment drop-out of women seeking drug and alcohol treatment in a specialist women's and two traditional mixed-sex treatment services. Br J Addict. 1992;87(6):883-90.	Not about barriers/facilitators	
A gay-identified alcohol treatment program: A follow-up study	Driscoll R. A gay-identified alcohol treatment program: a follow-up study. J Homosex. 1982;7(4):71-80.	Not about barriers/facilitators	
Acceptance and commitment therapy approach for problematic chemsex among men who have sex with men	Strika-Bruneau L, Karila L, Amirouche A, Fauvel B, Benyamina A. Acceptance and Commitment Therapy Approach for Problematic Chemsex Among Men Who Have Sex With Men. Cognitive and Behavioral Practice. 2024;31(4):451-468.	Not about barriers/facilitators	
Alcohol and other drug use, club drug dependence and treatment seeking among lesbian, gay and bisexual young people in Sydney	Lea T, Reynolds R, de Wit J. Alcohol and other drug use, club drug dependence and treatment seeking among lesbian, gay and bisexual young people in Sydney. Drug Alcohol Rev. 2013;32(3):303-11.	Not about barriers/facilitators	
Alcohol Use Disorder Treatment in Sexually and Gender Diverse Patients: A Retrospective Cohort Study	McDowell MJ, King DS, Gitin S, Miller AS, Batchelder AW, Busch AB, Greenfield SF, Huskamp HA, Keuroghlian AS. Alcohol Use Disorder Treatment in Sexually and Gender Diverse Patients: A Retrospective Cohort Study. J Clin Psychiatry. 2023;84(5):23m14812.	Not about barriers/facilitators	
Antisocial personality disorder predicts methamphetamine treatment outcomes in homeless, substance-dependent men who have sex with men	Fletcher JB, Reback CJ. Antisocial personality disorder predicts methamphetamine treatment outcomes in homeless, substance-dependent men who have sex with men. J Subst Abuse Treat. 2013;45(3):266-72.	Not about barriers/facilitators	

Are Specialized LGBT Program Components Helpful for Gay and Bisexual Men in Substance Abuse Treatment?	Senreich E. Are specialized LGBT program components helpful for gay and bisexual men in substance abuse treatment? Subst Use Misuse. 2010;45(7-8):1077-96.	Not about barriers/facilitators	More about treatment outcomes than barriers
Asking about sexual orientation during assessment for drug and alcohol concerns: a pilot study	Barbara AM & Chaim G. Asking About Sexual Orientation During Assessment for Drug and Alcohol Concerns: A Pilot Study. Journal of Social Work Practice in the Addictions. 2004;4(4):89–109.	Not about barriers/facilitators	
Assessing the feasibility of SMART Recovery for sexual and gender minority individuals: Results from a feasibility and preliminary outcomes trial	McGeough BL, Cohen NL & Greenwood E. Assessing the feasibility of SMART Recovery for sexual and gender minority individuals: Results from a feasibility and preliminary outcomes trial. Sexual and Gender Diversity in Social Services. 2023;36(3):437–466.	Not about barriers/facilitators	
Availability of Specific Programs and Medications for Addiction Treatment to Vulnerable Populations: Results from the Addiction Treatment Locator, Assessment, and Standards (ATLAS) Survey	Oldfield BJ, Chen K, Joudrey PJ, Biegacki ET, Fiellin DA. Availability of Specific Programs and Medications for Addiction Treatment to Vulnerable Populations: Results from the Addiction Treatment Locator, Assessment, and Standards (ATLAS) Survey. J Addict Med. 2023;17(4):477-480.	Not about barriers/facilitators	
Characteristics of lesbian, gay, bisexual, and transgender individuals entering substance abuse treatment	Cochran BN, Cauce AM. Characteristics of lesbian, gay, bisexual, and transgender individuals entering substance abuse treatment. J Subst Abuse Treat. 2006;30(2):135-46.	Not about barriers/facilitators	
Characteristics of Outpatient and Residential Substance Use Disorder Treatment Facilities with a Tailored LGBT Program	Ware OD, Austin AE, Srivastava A, Dawes HC, Baruah D, Hall WJ. Characteristics of Outpatient and Residential Substance Use Disorder Treatment Facilities with a Tailored LGBT Program. Subst Abuse. 2023;17:11782218231181274.	Not about barriers/facilitators	
Comparison of Government and Non-Government Alcohol and Other Drug (AOD) Treatment Service Delivery for the Lesbian, Gay, Bisexual, and Transgender (LGBT) Community	Mullens AB, Fischer J, Stewart M, Kenny K, Garvey S, Debattista J. Comparison of Government and Non-Government Alcohol and Other Drug (AOD) Treatment Service Delivery for the Lesbian, Gay, Bisexual, and Transgender (LGBT) Community. Subst Use Misuse. 2017;52(8):1027-1038.	Not about barriers/facilitators	Paper focuses on staff attitudes - not specifically conceptualised as barriers
Crystal methamphetamine use subgroups and associated addiction care access and overdose risk in a Canadian urban setting	Brooks O, Bach P, Dong H, Milloy MJ, Fairbairn N, Kerr T, Hayashi K. Crystal methamphetamine use subgroups and associated addiction care access and overdose risk in a Canadian urban setting. Drug Alcohol Depend. 2022;232:109274.	Not about barriers/facilitators	
Demographic Predictors of Dropping Out of Treatment (DOT) in Substance Use Disorder Treatment	Hanauer M, Sielbeck-Mathes K, Banks B, Mitori J, Reuveny A. Demographic Predictors of Dropping Out of Treatment (DOT) in Substance Use Disorder Treatment. Subst Use Misuse. 2021;56(8):1155-1160.	Not about barriers/facilitators	
Differences in assessment and treatment approaches for homosexual clients	MacEwan I. Differences in assessment and treatment approaches for homosexual clients. Drug and Alcohol Review. 1994;13(1):57–62.	Not about barriers/facilitators	Although paper discusses treatment differences between heterosexual and homosexual patients, not specifically conceptualised or elaborated as barriers/facilitators to access
Do specialized services exist for LGBT individuals seeking treatment for substance misuse? A study of available treatment programs	Cochran BN, Peavy KM, Robohm JS. Do specialized services exist for LGBT individuals seeking treatment for substance misuse? A study of available treatment programs. Subst Use Misuse. 2007;42(1):161-76.	Not about barriers/facilitators	
Does sexuality matter? A cross-sectional study of drug use, social injecting, and access to injection-specific care among men who inject drugs in Melbourne, Australia	Schroeder SE, Wilkinson AL, O'Keefe D, Bourne A, Doyle JS, Hellard M, Dietze P and Pedrana A. Does sexuality matter? A cross-sectional study of drug use, social injecting, and access to injection-specific care among men who inject drugs in Melbourne, Australia. Harm Reduction Journal. 2023;20:9.	Not about barriers/facilitators	

Drink goal difficulty effect on outcomes in moderation-based alcohol treatment for sexual minority men	Levak S, Kuerbis AN, Morgenstern J. Drink goal difficulty effect on outcomes in moderation-based alcohol treatment for sexual minority men. J Subst Abuse Treat. 2020;112:1-9.	Not about barriers/facilitators	Paper is more about treatment outcomes than barriers to access
Effects of Sexual Orientation and Gender on Perceived Need for Treatment by Persons With and Without Mental Disorders	Grella CE, Cochran SD, Greenwell L, Mays VM. Effects of sexual orientation and gender on perceived need for treatment by persons with and without mental disorders. Psychiatr Serv. 2011;62(4):404-10.	Not about barriers/facilitators	
Evaluation of an SMS Based Alcohol Intervention for Same Sex Attracted Women: A Randomized Controlled Trial to Examine Feasibility, Acceptability, and Efficacy	Bush R, Staiger PK, McNeill IM, Brown R, Orellana L, Lubman D, McNair R. Evaluation of an SMS Based Alcohol Intervention for Same Sex Attracted Women: A Randomized Controlled Trial to Examine Feasibility, Acceptability, and Efficacy. Subst Use Misuse. 2024;59(8):1157-1166.	Not about barriers/facilitators	
Factors Associated with Exchange Sex Among Cisgender Persons Who Inject Drugs: Women and MSM-23 US Cities, 2018	Rushmore J, Buchacz K, Broz D, Agnew-Brune CB, Jones MLJ, Cha S; NHBS Study Group. Factors Associated with Exchange Sex Among Cisgender Persons Who Inject Drugs: Women and MSM-23 U.S. Cities, 2018. AIDS Behav. 2023;27(1):51-64.	Not about barriers/facilitators	
Gay, Lesbian, Bisexual, and Transgendered People and Chemical Dependency: Exploring Successful Treatment	Rowan NL, Faul AC. Gay, Lesbian, Bisexual, and Transgendered People and Chemical Dependency: Exploring Successful Treatment. Journal of Gay & Lesbian Social Services. 2011;23(1):107–130.	Not about barriers/facilitators	
Governing beyond the closet: Remaking stigma, identity, and sexual behavior in a post-disciplinary rehab	Aleksanyan J. Governing beyond the closet: Remaking stigma, identity, and sexual behavior in a post-disciplinary rehab. Ethnography. 2020;23(4):516-538.	Not about barriers/facilitators	Ethnography including historical analysis, not specifically conceptualised as barriers to access
Health care utilization among young adult injection drug users in Harlem, New York	Cronquist A, Edwards V, Galea S, Latka M, Vlahov D. Health care utilization among young adult injection drug users in Harlem, New York. J Subst Abuse. 2001;13(1-2):17-27.	Not about barriers/facilitators	
Influence of gender, sexual orientation, and need on treatment utilization for substance use and mental disorders: findings from the California Quality of Life Survey	Grella CE, Greenwell L, Mays VM, Cochran SD. Influence of gender, sexual orientation, and need on treatment utilization for substance use and mental disorders: findings from the California Quality of Life Survey. BMC Psychiatry. 2009;9:52.	Not about barriers/facilitators	
Injection and Sexual Behavior Profiles among People Who Inject Drugs in Miami, Florida	Chueng TA, Tookes HE, McLaughlin M, Arcaro-Vinas AM, Serota DP, Bartholomew TS. Injection and Sexual Behavior Profiles among People Who Inject Drugs in Miami, Florida. Subst Use Misuse. 2022;57(9):1374-1382.	Not about barriers/facilitators	
Lesbian, Gay, and Bisexual Clients' Experiences in Treatment for Addiction	Matthews CR, Selvidge MM. Lesbian, gay, and bisexual clients' experiences in treatment for addiction. J Lesbian Stud. 2005;9(3):79-90.	Not about barriers/facilitators	More about factors associated with success of treatment than access to treatment itself
Longitudinal changes in alcohol and drug use among men seen at a gay-specific substance abuse treatment agency	Paul JP, Barrett DC, Crosby GM, Stall RD. Longitudinal changes in alcohol and drug use among men seen at a gay-specific substance abuse treatment agency. J Stud Alcohol. 1996;57(5):475-85.	Not about barriers/facilitators	
Meeting the Needs of Lesbian, Gay, and Bisexual Clients in Substance Abuse Treatment	Flentje A, Livingston NA, Sorensen JL. Meeting the Needs of Lesbian, Gay, and Bisexual Clients in Substance Abuse Treatment. Counselor (Deerfield Beach). 2016;17(3):54-59.	Not about barriers/facilitators	
Mental and Physical Health Needs of Lesbian, Gay, and Bisexual Clients in Substance Abuse Treatment	Flentje A, Livingston NA, Roley J, Sorensen JL. Mental and Physical Health Needs of Lesbian, Gay, and Bisexual Clients in Substance Abuse Treatment. J Subst Abuse Treat. 2015;58:78-83.	Not about barriers/facilitators	

Navigating drug use, cessation, and recovery: a retrospective case notes review among sexual minority men at a community-based service in Singapore	Wah TH, Ong AJX, Naidu KNC, Hanafi S, Tan K, Tan A, Ong TJJ, Ong E, Ho DWS, Subramaniam M, See MY, Tan RKJ. Navigating drug use, cessation, and recovery: a retrospective case notes review among sexual minority men at a community-based service in Singapore. Subst Abuse Treat Prev Policy. 2024;19(1):23.	Not about barriers/facilitators	
Pilot randomized controlled trial of an integrative intervention with methamphetamine-using men who have sex with men	Carrico AW, Gómez W, Siever MD, Discepola MV, Dilworth SE, Moskowitz JT. Pilot randomized controlled trial of an integrative intervention with methamphetamine-using men who have sex with men. Arch Sex Behav. 2015;44(7):1861-7.	Not about barriers/facilitators	
Predictors of moderated drinking in a primarily alcohol-dependent sample of men who have sex with men	Kuerbis A, Morgenstern J, Hail L. Predictors of moderated drinking in a primarily alcohol-dependent sample of men who have sex with men. Psychol Addict Behav. 2012;26(3):484-95.	Not about barriers/facilitators	
Preparing social workers for practice with LGBT populations affected by substance use: perceptions from students, alumni, and service providers	Dentato MP, Kelly BL, Lloyd MR & Busch N. Preparing Social Workers for Practice with LGBT Populations Affected by Substance Use: Perceptions from Students, Alumni, and Service Providers. Social Work Education. 2017;37(3):294–314.	Not about barriers/facilitators	
Queer quit: a pilot study of a smoking cessation programme tailored to gay men	Dickson-Spillmann M, Sullivan R, Zahno B, Schaub MP. Queer quit: a pilot study of a smoking cessation programme tailored to gay men. BMC Public Health. 2014;14:126.	Not about barriers/facilitators	
Reports of alcohol consumption and alcohol-related problems among homosexual, bisexual and heterosexual respondents: Results from the 2000 National Alcohol Survey	Drabble L, Midanik LT, Trocki K. Reports of alcohol consumption and alcohol-related problems among homosexual, bisexual and heterosexual respondents: results from the 2000 National Alcohol Survey. J Stud Alcohol. 2005;66(1):111-20.	Not about barriers/facilitators	
Service Provider Interpretations of Childhood Sexual Experiences Among Sexual Minority Men	Benoit E, Downing MJ, Brown D, Coe L. Service provider interpretations of childhood sexual experiences among sexual minority men. Psychol Serv. 2023;20(Suppl 1):94-102.	Not about barriers/facilitators	
Sexual orientation and substance abuse treatment utilization in the United States: results from a national survey	McCabe SE, West BT, Hughes TL, Boyd CJ. Sexual orientation and substance abuse treatment utilization in the United States: results from a national survey. J Subst Abuse Treat. 2013;44(1):4-12.	Not about barriers/facilitators	
Sexuality and Gender Identity Inequities in Substance Use Disorder and Its Treatment among American Indian, Alaska Native, and Native Hawaiian College Students	Qeadan F, Egbert J, Barbeau WA, Madden EF, Venner KL, English K. Sexuality and Gender Identity Inequities in Substance Use Disorder and Its Treatment among American Indian, Alaska Native, and Native Hawaiian College Students. Subst Use Misuse. 2022;57(14):2085-2093.	Not about barriers/facilitators	
Simulating the experience of searching for LGBTQ-specific opioid use disorder treatment in the United States	Paschen-Wolff MM, Velasquez R, Aydinoglo N, Campbell ANC. Simulating the experience of searching for LGBTQ-specific opioid use disorder treatment in the United States. J Subst Abuse Treat. 2022;140:108828.	Not about barriers/facilitators	
Social and health service use and treatment outcomes for sexual minorities in a national sample of substance abuse treatment programs	Hardesty M, Cao D, Shin HC, Andrews CM, & Marsh J. Social and Health Service Use and Treatment Outcomes for Sexual Minorities in a National Sample of Substance Abuse Treatment Programs. Journal of Gay & Lesbian Social Services. 2012; 24(2):97–118.	Not about barriers/facilitators	
Substance Use, Mental Health, and Service Access among Bisexual Adults in Australia	Loi B, Lea T, & Howard J. Substance Use, Mental Health, and Service Access among Bisexual Adults in Australia. Journal of Bisexuality. 2017;17(4):400–417.	Not about barriers/facilitators	
Substance Use among Lesbian, Gay, and Bisexual Clients Entering Substance Abuse Treatment: Comparisons to Heterosexual Clients	Flentje A, Heck NC, Sorensen JL. Substance use among lesbian, gay, and bisexual clients entering substance abuse treatment: Comparisons to heterosexual clients. J Consult Clin Psychol. 2015;83(2):325-34.	Not about barriers/facilitators	

Substance-related problems and treatment among men who have sex with men in comparison to other men in Chicago	Mackesy-Amiti ME, Fendrich M, Johnson TP. Substance-related problems and treatment among men who have sex with men in comparison to other men in Chicago. J Subst Abuse Treat. 2009;36(2):227-33.	Not about barriers/facilitators	
The 3-Year Course of Multiple Substance Use Disorders in the United States: A National Longitudinal Study	McCabe SE, West BT. The 3-Year Course of Multiple Substance Use Disorders in the United States: A National Longitudinal Study. J Clin Psychiatry. 2017;78(5):e537-e544.	Not about barriers/facilitators	
The availability of LGBT-specific mental health and substance abuse treatment in the United States	Williams ND, Fish JN. The availability of LGBT-specific mental health and substance abuse treatment in the United States. Health Serv Res. 2020;55(6):932-943.	Not about barriers/facilitators	Looking at predictors of services offering specialised LGBT programs
The Availability of Sexual and Gender Minority (SGM) Specific Substance Use Services	Ji CG, Cochran B. The Availability of Sexual and Gender Minority (SGM) Specific Substance Use Services. Subst Use Misuse. 2022;57(14):2126-2133.	Not about barriers/facilitators	
To what extent are key services offered in treatment programs for special populations?	Olmstead T, Sindelar JL. To what extent are key services offered in treatment programs for special populations? J Subst Abuse Treat. 2004;27(1):9-15.	Not about barriers/facilitators	
Treatment counselor's attitudes about lesbian, gay, bisexual, and transgendered clients: Urban vs. rural settings	Eliason MJ, Hughes T. Treatment counselor's attitudes about lesbian, gay, bisexual, and transgendered clients: urban vs. rural settings. Subst Use Misuse. 2004;39(4):625-44.	Not about barriers/facilitators	
Unmet mental health and substance abuse treatment needs of sexual minority elders	Jessup MA, Dibble SL. Unmet mental health and substance abuse treatment needs of sexual minority elders. J Homosex. 2012;59(5):656-74.	Not about barriers/facilitators	
Women Who Use Drugs and Have Sex with Women in a Canadian Setting: Barriers to Treatment Enrollment and Exposure to Violence and Homelessness	Lyons T, Shannon K, Richardson L, Simo A, Wood E, Kerr T. Women Who Use Drugs and Have Sex with Women in a Canadian Setting: Barriers to Treatment Enrollment and Exposure to Violence and Homelessness. Arch Sex Behav. 2016;45(6):1403-10.	Not about barriers/facilitators	
Rapid assessment of drug use and sexual HIV risk patterns among vulnerable drug-using populations in Cape Town, Durban and Pretoria, South Africa	Parry C, Petersen P, Carney T, Dewing S, Needle R. Rapid assessment of drug use and sexual HIV risk patterns among vulnerable drug-using populations in Cape Town, Durban and Pretoria, South Africa. SAHARA J. 2008;5(3):113-9.	Other	Duplicate paper - this paper appears to report on the same data about 78 MSM as in the other included paper by the same authors

Supplementary Table 7: Summary Table of Results

**Service-Related Determinants of Access**	**Barriers**
**Approachability**	APPROPRIATE SERVICES NOT EXISTING<br>• Addiction services being set up for alcohol or non-chemsex drug users rather than for chemsex[88,91,94]<br>• No available inpatient facilities for methamphetamine detoxification, with methamphetamine-using MSM deliberately consuming excessive alcohol to be eligible for detox admission[68]<br>• Services lacking specific expertise around LGBTQ+ addiction issues[67,68,74,81,86–88,91,93,94,100]<br><br>SERVICES NOT WORKING TOGETHER<br>• Fragmentation of services, patients being 'cycled' between services or 'knocking on several doors'[81,90,100]<br><br>SERVICES NOT BEING PUBLICISED<br>• Services failing to provide information about how drug services work and how they can help[89,95]
**Acceptability**	HARM AND DISCRIMINATION

	• Explicit harassment, discrimination, bullying, threats, violence, or abuse towards SM people within services, including patients being denied treatment due to their sexual identity[43,48,54,57,67,69,73,75,80,104]<br>• Staff failing to protect SM patients from hostility, overt homophobia, attacks, sexual violence, or abuse from non-SM patients in treatment settings[43,48,69,75,80,81]<br>• Explicit negative or stigmatising attitudes of service staff towards SM patients[46,48,57,58,69,75,80,85,86,91,94,105]<br>• Disclosure of SM status whilst accessing or already in treatment negatively affecting subsequent service provision[48,54,67,69,75,80]<br>• For chemsex participants, stigma around accessing needle exchanges and services (because they are associated with opiate drug users)[88]<br><br>UNMET TRAINING NEEDS<br>• Inadequate training/clinical supervision of staff around LGBTQ+ issues, or staff holding inaccurate information/myths/stereotypes about LGBTQ+ people[46,48,68,69,74,80,81,91,104]<br>• Services lacking insight into the unique needs of SM mothers[48,73,93]<br><br>INAPPROPRIATE SERVICE PROCESSES<br>• Heteronormative assumptions made on intake forms and during treatment[48,69,73,80,93]<br>• Drug services having a 'poor reputation'[66]<br>• Services not including the partners of gay/lesbian people in 'family' programmes[73]<br>• Services focusing on abstinence-only approaches[46,67]<br>• Organisational tensions between non-specialist and specialist LGBT programmes within the same service[53]
**Availability and Accommodation**	INCONVENIENT TIMES AND PLACES<br>• Inconvenient opening times (e.g. only 9am-5pm)[42,66,88,90]<br>• Geographical unavailability of services (e.g. outside main city areas)[56,66,78,94]<br>• No clear overall trend of the effect of rurality vs. urbanicity on unmet alcohol and substance use disorder treatment need[62]<br><br>INADEQUATE SERVICE PROVISION<br>• Problems with childcare provision at services[48,86]<br>• Long waiting lists (e.g. six months for a GBL/GHB detox)[66,101]<br>• Low numbers of SM patients leading to isolation within treatment programmes, or restricted provisions of SM-only services[53,54]<br>• Finite/limited resources of services evident (e.g. not giving naloxone to non-injecting drug users)[79]<br>• Staff feeling unable to accommodate the specific needs of SM patients[54]<br>• The type of treatment SM patients wanted not being offered[49]
**Affordability**	INADEQUATE FUNDING OF SERVICES<br>• Low cost or free addiction treatment programmes not being available in certain regions[102]<br>• Under-funded services being unable to pay for additional staff, rooms, or specialist training[53]<br><br>WIDER POLICY AND ECONOMIC FACTORS<br>• Health insurance not covering the cost of addiction treatment[49]<br>• Lack of economic resources preventing access to appropriate addiction-related medical services[46]
**Appropriateness**	LACKING LGBTQ+ CULTURAL COMPETENCE<br>• Service staff being negative/awkward about, or lacking knowledge about, the topic of sexuality and sexual practices[48,54,57,69,74,81,86,91,93,94]<br>• Missed opportunities by services and staff to address or explore important LGBTQ+ issues (such as trauma, coming out, sexualised drug use)[48,54,57,67,69,80,81,91,94,101]<br>• Staff stating that bisexuality did not exist[80]<br>• Staff not understanding the topic of sexual identity and motherhood[93]<br>• Services not acknowledging or addressing intersectionality (e.g. race/ethnicity and sexuality)[48]<br><br>INAPPROPRIATE TREATMENT

	• An inappropriate under-focus on sexuality (e.g. deflecting the topic even when patients explicitly linked substance use to the stresses associated with being SM)[48,54,69,80,86,94]<br>• An inappropriate over-focus on sexuality, making patients feel misunderstood or "different" (e.g. insisting homosexuality must be the cause of addiction problems)[48,54,69,80]<br>• Staff 'outing' patients without consent and breaching confidentiality[69,80]<br>• Instructing SM patients to hide their sexuality for the sake of non-SM patients in groups[48,54]<br>• Paternalistic, confrontational, or coercive consultation styles[48,69]<br>• Silencing patients who brought up chemsex-related topics[95]<br>• Staff believing that sexuality-conversion therapy was effective or practicing conversion therapy to try and make patients heterosexual[54,80]<br>• Services only targeting a middle-class clientele rather than patients with higher needs[43]

**Service-Related Determinants of Access**	**Facilitators**
**Approachability**	CREATING SPECIFIC SERVICES<br>• Creation or provision of SM-specific services or treatment pathways, including specific needs assessments[53,54,67,69,73,76,78,86,88,93,94,99]<br>• Creation of visibly LGBTQ+ safe/friendly environments (e.g. having LGBTQ+ positive waiting-room literature, or displaying Pride flags or rainbow stickers)[54,69,76,85,91,93]<br><br>SERVICES WORKING TOGETHER<br>• Holistic 'one-stop shop' services which could address addiction, sexual health, and mental health concerns in an integrated way[81]<br>• Partnership working (e.g. between addiction, sexual health, and emergency services, criminal justice agencies, housing providers, and LGBTQ+ organisations)[82,88,100]<br>• Accessing specialist drug services within sexual health clinics (e.g. because they were trusted as gay-friendly)[87,89]<br><br>IMPROVING REFERRAL INTO AND BETWEEN SERVICES<br>• Clear referral pathways into addiction services (e.g. following crisis hospitalisation, from sexual health services, and from public assistance services)[43,88,100,101]<br>• Provision of referral pathway options beyond the criminal justice system[88]<br>• Addiction service staff having knowledge of other local support services to facilitate onward referrals[76,81,91]<br>• Training medical staff (particularly in sexual health and HIV care) in identification and referral of chemsex participants into services[88]<br><br>PUBLICITY AND INFORMATION<br>• Publicity around drug support services directed at MSM[89]<br>• For existing services, outreach to the SM community to facilitate access (e.g. via smartphone hook-up apps, pop-up services, discussion groups, social networks, gay club nights/fetish venues, and commercial sex areas)[43,50,51,88,89,100]<br>• Improved information on pharmacotherapy for alcohol use disorder (AUD) using the language of harm reduction[50]
**Acceptability**	POSITIVE STAFF ATTITUDES AND BEHAVIOURS<br>• Positive, caring, affirmative, or non-judgemental attitudes from service providers (e.g. staff empathising with the difficulties of homophobia, taking time to ensure patients felt comfortable, being able to openly discuss gay sexuality, or acting as LGBTQ+ allies)[54,69,73,75,76,81,82,85,87,89,90,94,100,102]<br>• Service providers having 'cultural humility' or 'horizontal approaches', involving critical self-reflection and positioning the patient as the expert in their lives[69,81,90]<br>• Staff at needle exchange programmes providing informal referrals to less LGBT-hostile service environments, or acting as sources of solidarity[53,69]<br>• Staff explicitly letting patients know that their service is LGB-positive at their first contact[85]<br><br>INCLUSIVE SERVICE PROCESSES AND POLICIES<br>• Staff training (including for non-clinical staff) and policies about SM patients[54,69,73,93]<br>• Intake forms/processes using inclusive, open-ended language regarding sexuality and gender, and allowing non-disclosure of SM status[69,93]<br>• Particularly in countries with sanctions around drug use or SM status, services that can be used anonymously[96]

	LGBTQ+ INVOLVEMENT IN SERVICE DESIGN AND DELIVERY<br>• Seeing an openly LGBTQ+ healthcare professional, managerial and organisational support of diversity within drug services, and services recruiting LGBTQ+ staff[47,53,54,69,73,74,76,82,91,94]<br>• Inclusion of LGBTQ+ people in service development (e.g. formulating non-discrimination or staff vetting policies)[69]<br>• Positive LGBTQ+ community feedback enhancing the reputability of services[94]
**Availability and Accommodation**	CONVENIENT TIMES AND PLACES<br>• Establishment of evening and weekend drug clinics, offering alcohol treatment groups in non-clinical settings, and improved service provision in city centres[55,88]<br>• Locating services strategically in LGBTQ+ community areas or areas of high need (e.g. a needle exchange in the gay village, drop-in recovery services near where alcohol is sold)[55,88,89].<br>• For existing services, providing gender neutral bathrooms[53]<br><br>SERVICE DESIGN PREFERENCES<br>• For methamphetamine-using MSM, preferred drug service design characteristics include the availability of one-to-one support in addition to counsellor-led group counselling; long-term, open-ended support; and support located close to home[82]<br>• Having a separate treatment facility or separate unit/area within a service for SM patients[76]<br><br>MULTIPLE SERVICES WORKING TOGETHER<br>• For MSM, high access to substance abuse treatment programmes is typically correlated with high access to HIV risk reduction and education; medical care; mental health services; and a high education level[102]
**Affordability**	• Drop-in community recovery centres offering cheap or sliding scale payments[55]
**Appropriateness**	LGBTQ+ COMPETENT TREATMENT<br>• Professionals having specific knowledge, education, and ‘cultural competence’ about issues relevant to SM healthcare (e.g. methamphetamine use, coming out challenges, societal homophobia, links between drug use and sexual practices)[46,53,54,57,69,73,76,78,81,82,85,94]<br>• Service providers acknowledging sexuality as an important component of identity whilst not assuming it is the cause of a patient’s problems[69,73,76,93]<br>• Providers “meeting the client where they are at” (e.g. reflecting patients’ own language and identity labels, or offering flexibly tailored treatment)[45,69,76]<br><br>POSITIVE, APPROPRIATE TREATMENT<br>• Open, non-judgemental questioning about gender and sexual preference during intake processes for new patients [53,54,69,73,93]<br>• Matching patients to therapists with their preferred sexual and/or gender identity[53]<br>• Staff using a warm, hopeful consultation style which preserves the patient's decision-making power[48,76,81]<br>• Approaches which are trauma-informed and sex-positive[57,69]<br>• Provision of relevant harm reduction supplies (e.g. clean needles)[69,82,100]<br><br>HOLISTIC APPROACHES<br>• Holistic, intersectional, or ‘whole person’ approaches to treatment (e.g. incorporating sexuality, substance abuse, housing and social support, subsistence needs, mental health, culture, and spirituality)[48,54,57,69,73,76,81,82,90]<br>• Services being inclusive of HIV education and advocacy[57]<br>• Services having the ability to link patients to wider support programmes (e.g. a SM women’s group)[53,81]

**Patient-Related Determinants of Access**	**Barriers**

**Ability to Perceive**	NOT KNOWING ABOUT SERVICES<br>• Not knowing that addiction treatment was available/existed[42,50,66,79,94,97]<br>• Not knowing where or how to access services[49,66,79,98]<br><br>NOT WANTING SERVICES<br>• Ambivalence around drug/alcohol use or reluctance to change[42,47,50,58,67,98,101]<br>• Not seeing drug/alcohol use as a problem or serious problem[42,79,89,94,98,101]<br>• Normalisation of drug/alcohol use among SM people/peers[101]<br><br>NOT KNOWING IF SERVICES ARE RIGHT<br>• Not being sure if they would be eligible for treatment[66]<br>• Feeling the type of treatment they wanted was not offered from services[49]<br>• Not believing addiction treatment was appropriate to their needs[48,79]<br>• For lesbians, a sense of being 'different and isolated' leading to a feeling that conventional healthcare is not designed for their needs[48]<br><br>NOT BELIEVING SERVICES COULD HELP<br>• Negative beliefs around addiction or its treatment (e.g. feeling 'I should be strong enough to handle this alone', not believing that 'alcoholism' is a treatable condition, or feeling that GBL/GHB users were treated inadequately by services)[42,66,97,98,101]<br>• Concerns about other drug users, or rehabilitation itself, triggering relapse[67,79,98]<br><br>DRUG USE PREVENTING SERVICE ACCESS<br>• For MSM, greater frequency of methamphetamine use, and greater frequency of sexualised use, is associated with an increased perceived difficulty of accessing support[84]
**Ability to Seek**	FEAR OF NEGATIVE OR PUNITIVE EXPERIENCES<br>• Fears or embarrassment about discussing addiction or treatment[42,66,73]<br>• Apprehension about discussing sexualised drug or alcohol use[75,79,87,89]<br>• Worrying about involuntary admission to hospital if services are accessed[42]<br>• For lesbians, fears that children will be taken into care if they enter treatment services[48]<br>• Fear of incarceration/criminal sanctions for drug use or gay sex, or being reported by staff to drug law enforcement agencies [95,96]<br>• Not accessing services due to fears of legal ramifications[94]<br><br>PREVIOUS NEGATIVE EXPERIENCES<br>• Previous unsuccessful attempts to access or utilise treatment[42,98,101]<br><br>CONCERNS ABOUT STIGMA<br>• Fears of others' opinions on learning about a person's addiction problems[42,73]<br>• Fear of stigma due to SM status, alcohol/drug use (particularly injecting), or addiction treatment itself [48,50,56,69,70,73,75,76,79,85,86,94–96]<br>• Concerns around being given pathologising labels (e.g. 'alcoholic') compounding the social stigma already experienced [45,48,86]<br><br>NEEDING COMMITMENT<br>• For services that are difficult to physically access, an increased commitment is needed to attend[56]<br><br>SPECIFIC 'MINORITY-WITHIN-A-MINORITY' FACTORS<br>• For Black non-gay identified men who have sex with men and women, not seeking treatment due to cultural constructions of masculinity[45]<br>• For Black MSM, barriers to methamphetamine treatment are a racialised problem: caused by exposure to poverty, inadequate education, and few resources compared with white MSM[68]

	• For gay/lesbian-specific treatment units, a focus on white men can mean that they are perceived as less welcoming for women and non-white people[76]<br>• Women experiencing lifetime high risk drug use were more likely than moderate or low risk users to report that they had wanted but had not received professional support[44]<br>• Compared with gay men, bisexual men were reported to have both a decreased[63,65] and increased[83] likelihood of drug treatment utilisation, and an increased likelihood of alcohol treatment utilisation[71]<br>• Compared with a matched cohort of heterosexual men, SM men ≥50 years old had a lower rate of inpatient drug treatment utilisation, and compared with heterosexual women, SM women ≥50 years old had a higher rate of inpatient drug treatment utilisation[72]<br>• Black bisexual women had higher odds of reporting a treatment gap for both specialty drug, and alcohol services (compared with Black bisexual men, gay men, or lesbian women), and Black gay men had worse odds of experiencing a drug specialty treatment gap than Black bisexual men[59]<br>• Latina SM women were less likely to access substance use treatment services compared with white SM women[77]<br>• Compared with SM men, SM women with probable AUD were less likely to have this diagnosed by a professional, and less likely to attend substance use-related appointments[60]
**Ability to Reach**	LACKING APPROPRIATE FAMILY OR SOCIAL SUPPORT<br>• Black lesbian and bisexual women have less social support to help with accessing alcohol treatment than for heterosexual women[52]<br>• For MSM, family members forcing them enter treatment may create resistance to the idea[67]<br><br>TRANSPORT DIFFICULTIES<br>• A car may be required to access services, or patients may need to pay others to drive them [56]
**Ability to Pay**	INDIVIDUAL FINANCIAL FACTORS<br>• Being unable to afford medical bills/pay for addiction services, or concerns about the potential costs[42,46,56,66]<br>• For medications for AUD, concerns around cost to benefits ratio of medications[50]<br><br>WIDER POLICY FACTORS<br>• Problems with obtaining health insurance, which may be dependent on employment[56,78]
**Ability to Engage**	IDENTITY CONCEALMENT AND ALIENATION<br>• Patients feeling the need to present in a heteronormative manner[80]<br>• Patients feeling the need to censor/not disclose their sexuality[75,85,94]<br>• Treatment being unsuccessful or less successful if someone feels unable to disclose their sexuality or sexual practices[75,85,94]<br>• Feelings of isolation or alienation (i.e. as a minority within predominantly heterosexual) services or therapeutic groups[67,75,76]<br><br>FEAR OR MISTRUST OF HEALTHCARE PROVIDERS<br>• Mistrust in healthcare providers (e.g. patients feeling that healthcare providers have negative/pathologising attitudes or inadequate knowledge, or they have to educate their healthcare providers about LGBTQ+ related issues)[48,69,85,94]<br>• Patients fearing homophobia, shaming, rejection, or abuse based on their sexual identity within treatment[54,69,75,76,86,94]<br>• Difficulties discussing sexual practices or identity in therapeutic relationships, or fearing that staff members cannot 'relate' to SM patients [54,69,75,85,89,94]<br><br>INDIVIDUAL MOTIVATION FACTORS<br>• Patients not wanting to engage with services, or wanting to continue alcohol/drug use[42]<br>• Difficulty staying sober because of being unable to enjoy sex without drugs[46]<br>• For medication for AUD, concerns around the burden of treatment, and fears that it might negatively impact enjoyment of alcohol[50,97]<br><br>PRACTICAL AND CONFIDENTIALITY CONCERNS<br>• Not having time to attend a service[66]<br>• Difficulty in keeping appointments during periods of heavy substance use[56]<br>• Concerns with confidentiality when accessing services[66,73,94]

Patient-Related Determinants of Access	Facilitators
**Ability to Perceive**	KNOWING ABOUT SERVICES<br>• Knowledge of addiction support services and treatment options (e.g. pharmacological treatment of AUD)[50,101]<br><br>FINDING INFORMATION ABOUT SERVICES<br>• Peer advocates and LGBTQ+ networks helping patients to understand their addiction problems and promoting treatment[43,58,67]<br><br>HAVING MOTIVATION TO CHANGE<br>• For chemsex participants, motivation to access services may occur when they perceive it to be detrimental, or to avoid related financial, housing, or employment crises[94]
**Ability to Seek**	*No reported facilitators*
**Ability to Reach**	LGBTQ+ PEERS AND NETWORKS FACILITATING SERVICE ENTRY<br>• Signposting and referral to services through LGBTQ+ community contacts, networks, peer advocates, family and friends[43,58,67,101]<br>• For MSM, use of substance abuse treatment programmes is positively associated with connection to the gay community[102]<br><br>HAVING FAMILY SUPPORT<br>• Family support viewed as important for successful treatment[97]<br>• Gay/lesbian people being able to bring 'whoever you want to be considered family' into family meetings as part of treatment[73]<br><br>CRIMINAL SYSTEM INVOLVEMENT<br>• SM individuals with a history of criminal legal system involvement are more likely to utilise drug treatment than SM individuals without this history[64]
**Ability to Pay**	FLEXIBILITY IN PAYING FOR SERVICES<br>• Patients using private services (paid for directly or by insurance) even when free public healthcare was available, because of faster access or needing expertise not available in the public health sector[81]<br>• State addiction treatment funding being able to be used to access an LGBTQ-specific treatment centre[78]<br><br>NOMINAL FEES<br>• Providing AUD treatment for a nominal fee to allow for more buy-in with the idea of treatment[97]<br><br>HAVING SIMILAR PEERS<br>• For MSM, the importance of other participants in drug treatment being in similar financial situations to themselves[82]
**Ability to Engage**	HAVING SPECIFIC MINORITY-WITHIN-MINORITY SERVICES<br>• For SM women, having specialist women-only services is associated with greater likelihood of completing treatment[92]<br>• Having specialist female-only or lesbian-only groups allows SM women to discuss specific relevant issues (e.g. sexual harassment and gender roles)[55,86]<br><br>PEER SUPPORT AND LGBTQ+ COMMUNITY CONNECTION<br>• Peer support around GHB/GBL use for MSM[101]<br>• Building opportunities for socialising, self-expression, community connection, and mutual support (particularly with other SM people) within treatment[67,73,75,76,82]<br>• The importance of SM role models who were in recovery from addiction themselves; both other patients and staff[73,94]<br>• Having other treatment participants who identify as SM or LGBTQ+ [69,73,75,76,82]<br><br>ENCOURAGING AUTHENTICITY, FOREGROUNDING AUTONOMY<br>• For gay and lesbian patients, being able to 'work though shame to self-acceptance'[73]

	• Focus of treatment being use reduction or harm reduction rather than abstinence, or allowing patients to set their own goals[55,67,69,82,97]<br>• Being able to be honest and open about the specific context of sexuality and addiction[73,75,76]<br><br>NON-CLINICAL SETTINGS<br>• For individuals with mild or moderate alcohol problems, they may be more likely to engage in a group in a non-clinical setting[55]<br><br>SPECIFIC RECOVERY FACILITATORS<br>• Both SM and heterosexual participants reported that their top addiction recovery facilitators were friends, community and network; family and children; and 12-step programmes such as Alcoholics Anonymous. More SM than heterosexual participants cited spirituality, education, and loss (e.g. of identity, loved ones, agency) as facilitators to recovery[61]

Supplementary Table 8: PRISMA Checklist

Section and Topic	Item #	Checklist item	Location where item is reported (page number)
**TITLE**			
Title	1	Identify the report as a systematic review.	1
**ABSTRACT**			
Abstract	2	See the PRISMA 2020 for Abstracts checklist.	1
**INTRODUCTION**			
Rationale	3	Describe the rationale for the review in the context of existing knowledge.	2
Objectives	4	Provide an explicit statement of the objective(s) or question(s) the review addresses.	2
**METHODS**			
Eligibility criteria	5	Specify the inclusion and exclusion criteria for the review and how studies were grouped for the syntheses.	3, OSM
Information sources	6	Specify all databases, registers, websites, organisations, reference lists and other sources searched or consulted to identify studies. Specify the date when each source was last searched or consulted.	3
Search strategy	7	Present the full search strategies for all databases, registers and websites, including any filters and limits used.	OSM
Selection process	8	Specify the methods used to decide whether a study met the inclusion criteria of the review, including how many reviewers screened each record and each report retrieved, whether they worked independently, and if applicable, details of automation tools used in the process.	4
Data collection process	9	Specify the methods used to collect data from reports, including how many reviewers collected data from each report, whether they worked independently, any processes for obtaining or confirming data from study investigators, and if applicable, details of automation tools used in the process.	4
Data items	10a	List and define all outcomes for which data were sought. Specify whether all results that were compatible with each outcome domain in	4, OSM

Section and Topic	Item #	Checklist item	Location where item is reported (page number)
		each study were sought (e.g. for all measures, time points, analyses), and if not, the methods used to decide which results to collect.	
	10b	List and define all other variables for which data were sought (e.g. participant and intervention characteristics, funding sources). Describe any assumptions made about any missing or unclear information.	4, OSM
Study risk of bias assessment	11	Specify the methods used to assess risk of bias in the included studies, including details of the tool(s) used, how many reviewers assessed each study and whether they worked independently, and if applicable, details of automation tools used in the process.	4
Effect measures	12	Specify for each outcome the effect measure(s) (e.g. risk ratio, mean difference) used in the synthesis or presentation of results.	N/A
Synthesis methods	13a	Describe the processes used to decide which studies were eligible for each synthesis (e.g. tabulating the study intervention characteristics and comparing against the planned groups for each synthesis (item #5)).	4
	13b	Describe any methods required to prepare the data for presentation or synthesis, such as handling of missing summary statistics, or data conversions.	4
	13c	Describe any methods used to tabulate or visually display results of individual studies and syntheses.	N/A
	13d	Describe any methods used to synthesize results and provide a rationale for the choice(s). If meta-analysis was performed, describe the model(s), method(s) to identify the presence and extent of statistical heterogeneity, and software package(s) used.	4
	13e	Describe any methods used to explore possible causes of heterogeneity among study results (e.g. subgroup analysis, meta-regression).	N/A
	13f	Describe any sensitivity analyses conducted to assess robustness of the synthesized results.	N/A
Reporting bias assessment	14	Describe any methods used to assess risk of bias due to missing results in a synthesis (arising from reporting biases).	4
Certainty assessment	15	Describe any methods used to assess certainty (or confidence) in the body of evidence for an outcome.	4
**RESULTS**			
Study selection	16a	Describe the results of the search and selection process, from the number of records identified in the search to the number of studies included in the review, ideally using a flow diagram.	4
	16b	Cite studies that might appear to meet the inclusion criteria, but which were excluded, and explain why they were excluded.	OSM
Study characteristics	17	Cite each included study and present its characteristics.	5
Risk of bias in studies	18	Present assessments of risk of bias for each included study.	5, OSM
Results of	19	For all outcomes, present, for each study: (a) summary statistics for each group (where appropriate) and (b) an effect estimate and its	5-12

Section and Topic	Item #	Checklist item	Location where item is reported (page number)
individual studies		precision (e.g. confidence/credible interval), ideally using structured tables or plots.	
Results of syntheses	20a	For each synthesis, briefly summarise the characteristics and risk of bias among contributing studies.	5
	20b	Present results of all statistical syntheses conducted. If meta-analysis was done, present for each the summary estimate and its precision (e.g. confidence/credible interval) and measures of statistical heterogeneity. If comparing groups, describe the direction of the effect.	N/A
	20c	Present results of all investigations of possible causes of heterogeneity among study results.	N/A
	20d	Present results of all sensitivity analyses conducted to assess the robustness of the synthesized results.	N/A
Reporting biases	21	Present assessments of risk of bias due to missing results (arising from reporting biases) for each synthesis assessed.	5, OSM
Certainty of evidence	22	Present assessments of certainty (or confidence) in the body of evidence for each outcome assessed.	5, OSM
**DISCUSSION**			
Discussion	23a	Provide a general interpretation of the results in the context of other evidence.	11-12
	23b	Discuss any limitations of the evidence included in the review.	12
	23c	Discuss any limitations of the review processes used.	12
	23d	Discuss implications of the results for practice, policy, and future research.	13
**OTHER INFORMATION**			
Registration and protocol	24a	Provide registration information for the review, including register name and registration number, or state that the review was not registered.	3
	24b	Indicate where the review protocol can be accessed, or state that a protocol was not prepared.	3
	24c	Describe and explain any amendments to information provided at registration or in the protocol.	N/A
Support	25	Describe sources of financial or non-financial support for the review, and the role of the funders or sponsors in the review.	13
Competing interests	26	Declare any competing interests of review authors.	14
Availability of data, code and other materials	27	Report which of the following are publicly available and where they can be found: template data collection forms; data extracted from included studies; data used for all analyses; analytic code; any other materials used in the review.	OSM
